# Supplementary material for: The HSV-1 ICP22 protein selectively impairs histone repositioning upon Pol II transcription downstream of genes
Source: Nat Commun. 2023 Jul 31;14:4591. doi: 10.1038/s41467-023-40217-w (PMC10390501; doi:10.1038/s41467-023-40217-w)
Supplement: Supplementary file 1 — Supplementary Information [file 41467_2023_40217_MOESM1_ESM.pdf]

a

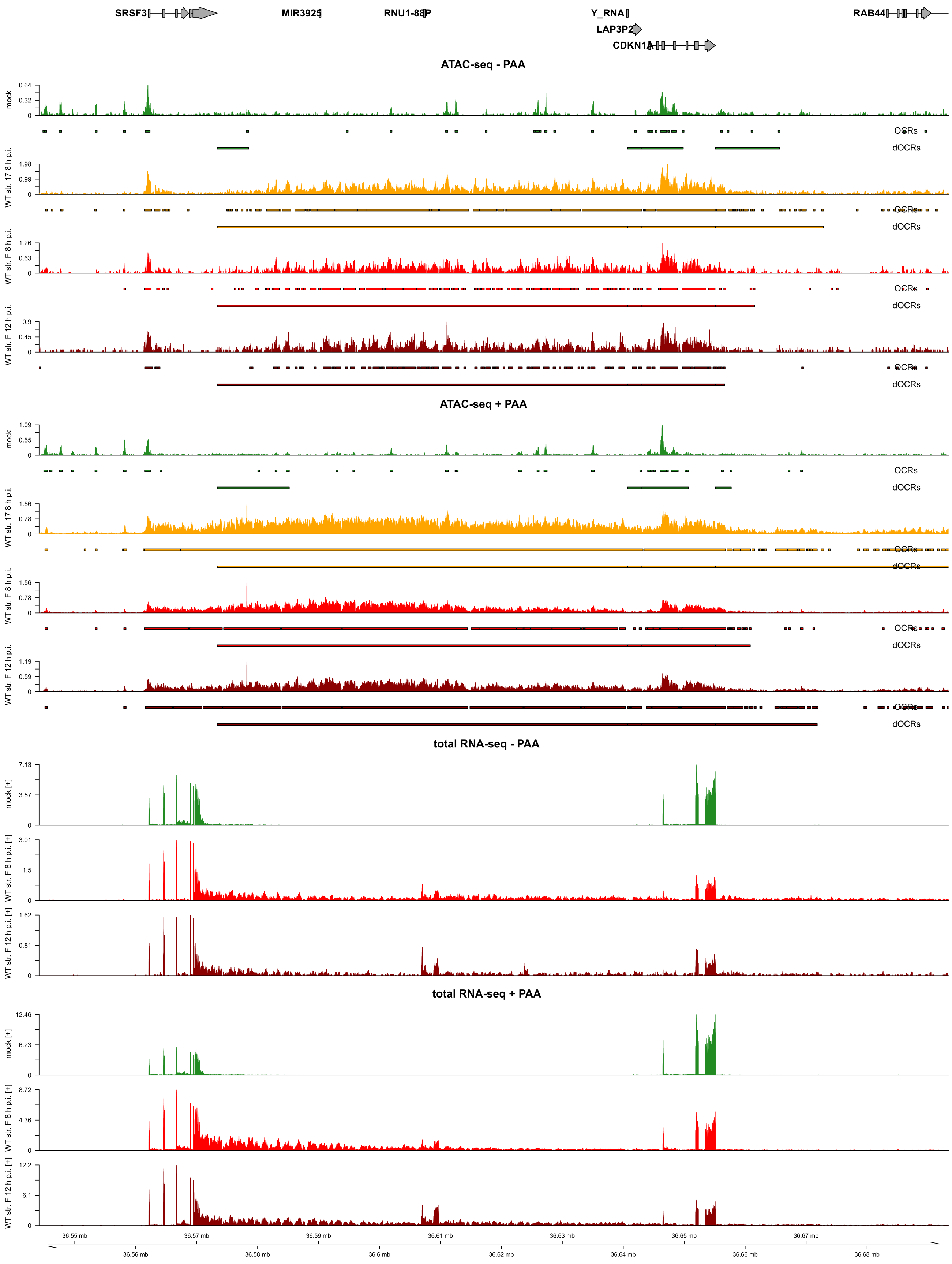
SRSF3
MIR3925
RNU1-88P
Y_RNA
LAP3P2
CDKN1A
RAB44
ATAC-seq - PAA
mock
WT str. 17 8 h p.i.
WT str. F 8 h p.i.
WT str. F 12 h p.i.
OCRs
dOCRs
ATAC-seq + PAA
total RNA-seq - PAA
mock [+]
WT str. F 8 h p.i. [+]
WT str. F 12 h p.i. [+]
total RNA-seq + PAA
36.55 mb
36.56 mb
36.57 mb
36.58 mb
36.59 mb
36.6 mb
36.61 mb
36.62 mb
36.63 mb
36.64 mb
36.65 mb
36.66 mb
36.67 mb
36.68 mb

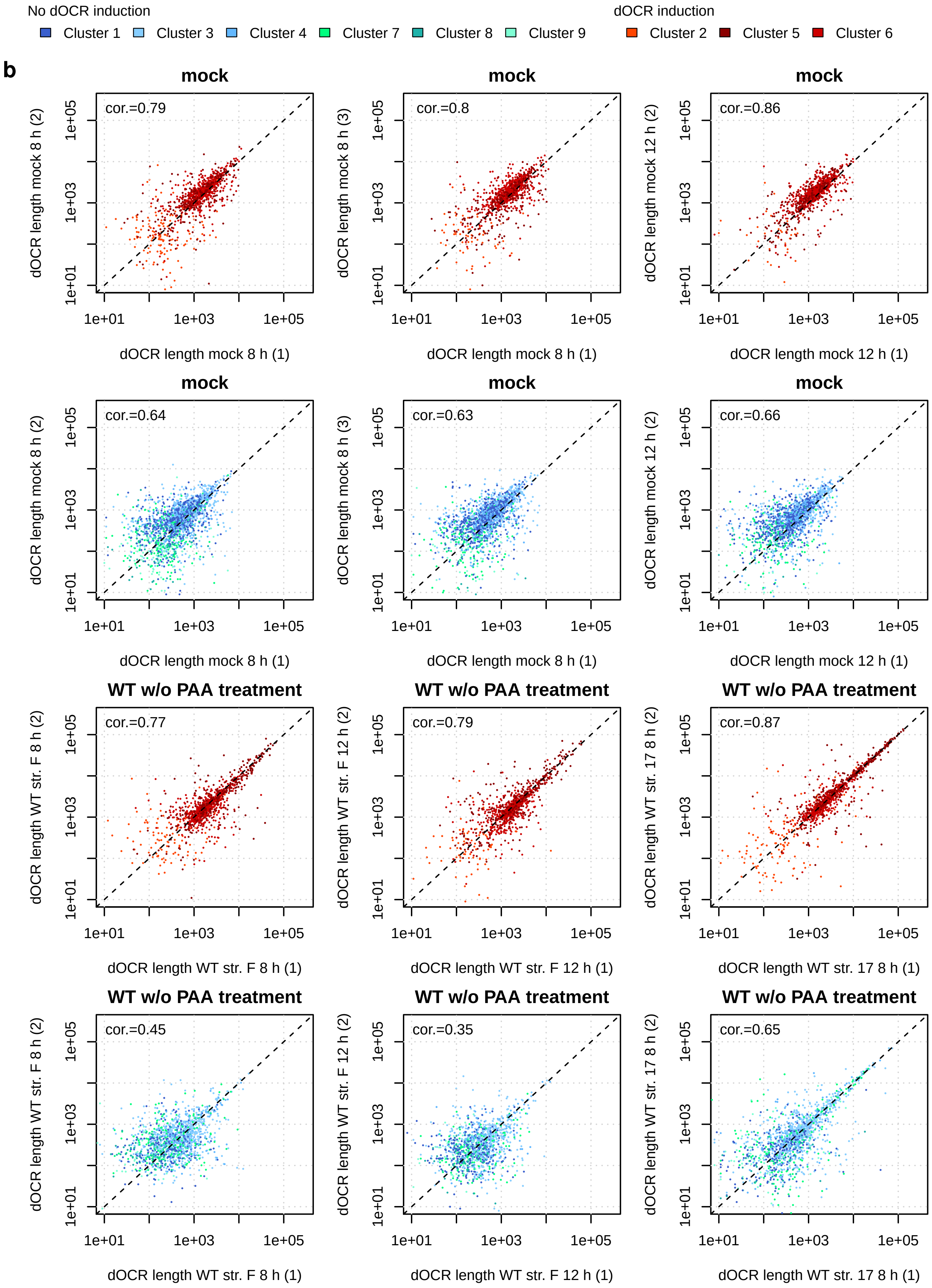
No dOCR induction
Cluster 1
Cluster 3
Cluster 4
Cluster 7
Cluster 8
Cluster 9
dOCR induction
Cluster 2
Cluster 5
Cluster 6
b
mock
cor.=0.79
dOCR length mock 8 h (2)
dOCR length mock 8 h (1)
mock
cor.=0.8
dOCR length mock 8 h (3)
dOCR length mock 8 h (1)
mock
cor.=0.86
dOCR length mock 12 h (2)
dOCR length mock 12 h (1)
mock
cor.=0.64
dOCR length mock 8 h (2)
dOCR length mock 8 h (1)
mock
cor.=0.63
dOCR length mock 8 h (3)
dOCR length mock 8 h (1)
mock
cor.=0.66
dOCR length mock 12 h (2)
dOCR length mock 12 h (1)
WT w/o PAA treatment
cor.=0.77
dOCR length WT str. F 8 h (2)
dOCR length WT str. F 8 h (1)
WT w/o PAA treatment
cor.=0.79
dOCR length WT str. F 12 h (2)
dOCR length WT str. F 12 h (1)
WT w/o PAA treatment
cor.=0.87
dOCR length WT str. 17 8 h (2)
dOCR length WT str. 17 8 h (1)
WT w/o PAA treatment
cor.=0.45
dOCR length WT str. F 8 h (2)
dOCR length WT str. F 8 h (1)
WT w/o PAA treatment
cor.=0.35
dOCR length WT str. F 12 h (2)
dOCR length WT str. F 12 h (1)
WT w/o PAA treatment
cor.=0.65
dOCR length WT str. 17 8 h (2)
dOCR length WT str. 17 8 h (1)
1e+01
1e+03
1e+05

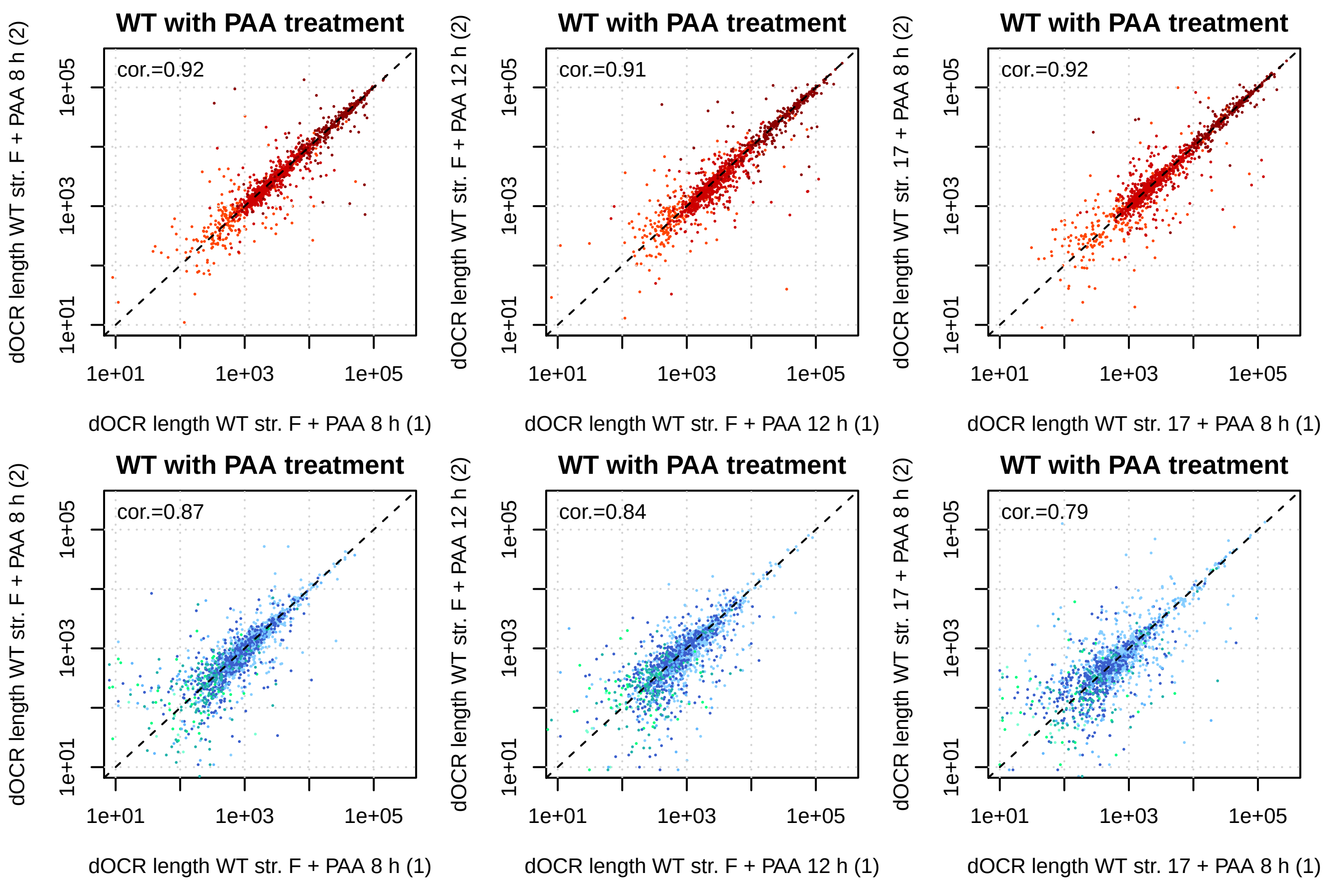
WT with PAA treatment
cor.=0.92
dOCR length WT str. F + PAA 8 h (2)
dOCR length WT str. F + PAA 8 h (1)
WT with PAA treatment
cor.=0.91
dOCR length WT str. F + PAA 12 h (2)
dOCR length WT str. F + PAA 12 h (1)
WT with PAA treatment
cor.=0.92
dOCR length WT str. 17 + PAA 8 h (2)
dOCR length WT str. 17 + PAA 8 h (1)
WT with PAA treatment
cor.=0.87
dOCR length WT str. F + PAA 8 h (2)
dOCR length WT str. F + PAA 8 h (1)
WT with PAA treatment
cor.=0.84
dOCR length WT str. F + PAA 12 h (2)
dOCR length WT str. F + PAA 12 h (1)
WT with PAA treatment
cor.=0.79
dOCR length WT str. 17 + PAA 8 h (2)
dOCR length WT str. 17 + PAA 8 h (1)
1e+01
1e+03
1e+05

c

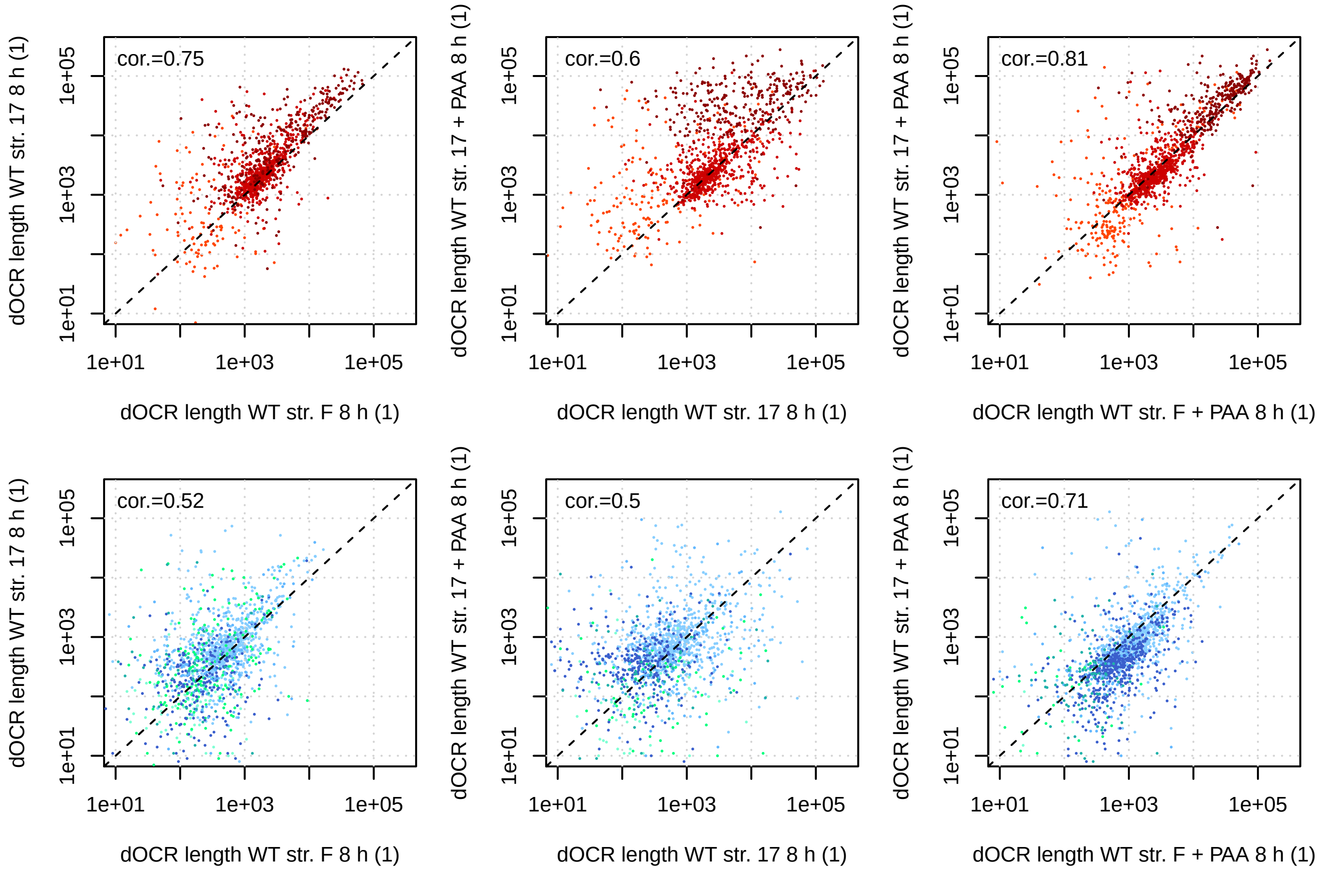
cor.=0.75
dOCR length WT str. 17 8 h (1)
dOCR length WT str. F 8 h (1)
cor.=0.6
dOCR length WT str. 17 + PAA 8 h (1)
dOCR length WT str. 17 8 h (1)
cor.=0.81
dOCR length WT str. 17 + PAA 8 h (1)
dOCR length WT str. F + PAA 8 h (1)
cor.=0.52
dOCR length WT str. 17 8 h (1)
dOCR length WT str. F 8 h (1)
cor.=0.5
dOCR length WT str. 17 + PAA 8 h (1)
dOCR length WT str. 17 8 h (1)
cor.=0.71
dOCR length WT str. 17 + PAA 8 h (1)
dOCR length WT str. F + PAA 8 h (1)
1e+01
1e+03
1e+05

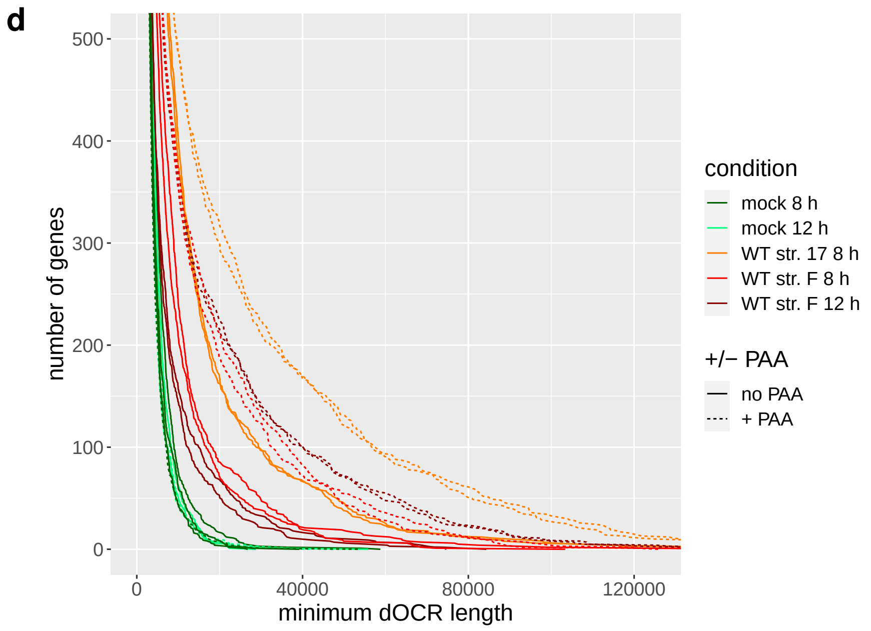

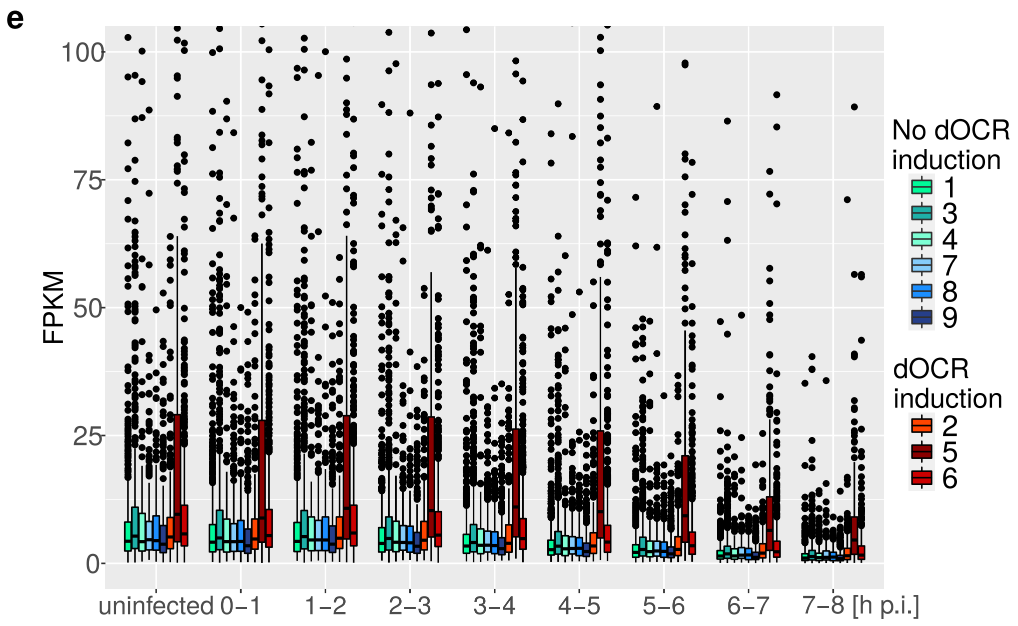

Sup. Fig. 1: (a) ATAC-seq and total RNA-seq data (if performed in parallel to ATAC-seq) for mock, WT strain 17 (no parallel total RNA-seq) and WT strain F infection without and with PAA treatment for an example gene with strong read-through and dOCR induction (SRSF3). The top eight tracks show ATAC-seq read coverage (non-strand-specific) and the bottom six tracks show matching total RNA-seq data (strand-specific). Read coverage was normalized to total number of mapped human reads and averaged between replicates. Rectangles below each ATAC-seq track show open chromatin regions (OCRs) identified with F-Seq as well as the dOCR regions calculated from OCRs as described in Methods. OCRs and dOCRs are shown only for the first replicate for simplification. Gene annotation is indicated at the top. Boxes represent exons and lines introns and direction is indicated by arrowheads. Genomic coordinates are shown on the bottom. **(b,c)** Scatter plots comparing dOCR lengths between **(b)** replicates of the same condition and **(c)** samples from different conditions. Scatter plots are shown above each other separately for genes with dOCR induction (clusters 2, 5, and 6 from Fig. 1b) and genes without dOCR induction (remaining clusters, color coding for clusters shown on top of **(b)**). Equal values are indicated by the dashed diagonal line and genes are colored according to the 9 clusters (n = 609, 290, 851, 176, 305, 701, 367, 289, and 574 genes for clusters 1-9, respectively) in Fig. 1b. Spearman rank correlation between samples on the x- and y-axis is shown in the top left corner of each panel. **(d)** Number of genes (y-axis) for which dOCRs reach a length greater than the value indicated on the x-axis in mock and HSV-1 WT

strain 17 and strain F infection after down-sampling to approximately the same number of reads mapped to the human genome. For each condition, this figure includes all analyzed genes without read-in transcription with a dOCR length > 0 for that particular condition. The y-axis was furthermore limited to 500 to highlight the difference in the number of genes with long dOCRs between mock and HSV-1 infection. **(e)** Boxplots showing the distribution of gene expression (gene FPKM) for genes in the 9 clusters (n = 609, 290, 851, 176, 305, 701, 367, 289, and 574 genes for clusters 1-9, respectively) in Fig. 1b. Gene FPKM values were calculated from our published 4sU-seq time-course for the first 8 h of WT strain 17 infection (Rutkowski *et al.*, Nature communications, 2015, 6:7126). Bounds of boxes are the first and third quartiles for each condition. The center (median) is shown by the horizontal line in the box. Whiskers extend to 1.5 times the inter-quartile range. Outliers are shown as small circles and minimum and maximum values are lowest and highest circles, respectively. Statistical significance for median FPKM values being higher for each cluster compared to genes in all other clusters was determined using one-sided Wilcoxon rank sum tests. Resulting p-values are given below:

cl.	h p.i.								
	uninf.	0-1	1-2	2-3	3-4	4-5	5-6	6-7	7-8
1	1	1	1	1	1	1	1	1	1
2	0.56	0.49	0.64	0.64	0.49	0.26	0.25	0.054	0.031
3	0.025	0.03	0.053	0.084	0.16	0.25	0.32	0.45	0.62
4	0.94	0.94	0.95	0.92	0.96	0.95	0.95	0.99	0.99
5	2.7×10^{-24}	1.6×10^{-25}	1.4×10^{-31}	6.7×10^{-43}	3.9×10^{-55}	2.8×10^{-65}	5.5×10^{-69}	1.1×10^{-68}	8.3×10^{-63}
6	5.4×10^{-7}	8×10^{-7}	6×10^{-8}	4.8×10^{-10}	4.1×10^{-10}	1.6×10^{-11}	4.8×10^{-12}	9.1×10^{-11}	1.1×10^{-8}
7	0.98	0.98	0.99	0.98	0.98	0.98	0.96	0.94	0.83
8	0.97	0.97	0.98	0.99	1	1	1	1	0.99
9	1	1	1	1	1	1	1	1	1

Both Cluster 5 (strong induction of dOCRs) and Cluster 6 are characterized by significantly higher FPKM values than remaining genes already in uninfected cells and throughout infection ($p < 0.0005$).

a
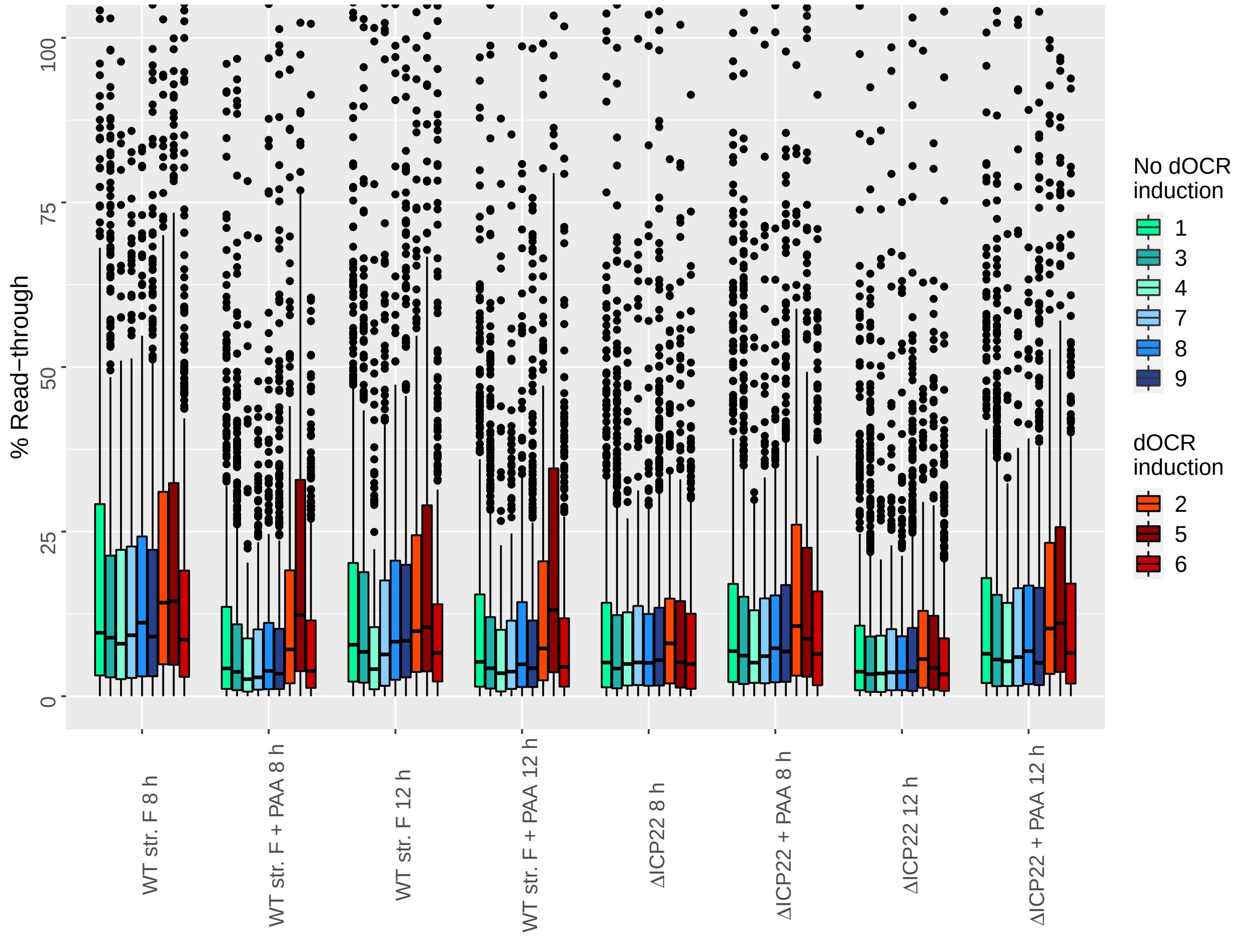
% Read−through
0
25
50
75
100
WT str. F 8 h
WT str. F + PAA 8 h
WT str. F 12 h
WT str. F + PAA 12 h
ΔICP22 8 h
ΔICP22 + PAA 8 h
ΔICP22 12 h
ΔICP22 + PAA 12 h
No dOCR induction
1
3
4
7
8
9
dOCR induction
2
5
6

b
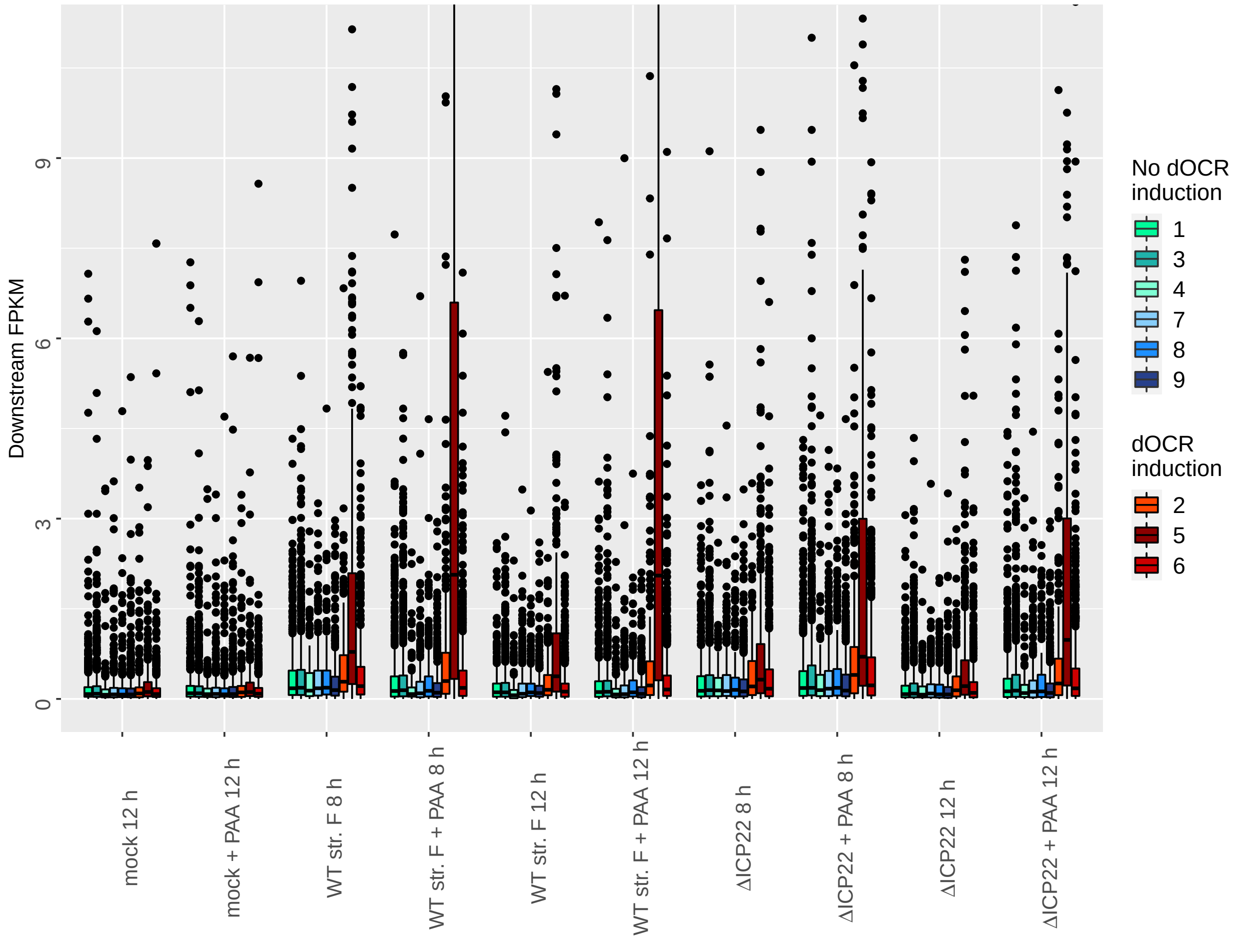
Downstream FPKM
0
3
6
9
mock 12 h
mock + PAA 12 h
WT str. F 8 h
WT str. F + PAA 8 h
WT str. F 12 h
WT str. F + PAA 12 h
ΔICP22 8 h
ΔICP22 + PAA 8 h
ΔICP22 12 h
ΔICP22 + PAA 12 h
No dOCR induction
1
3
4
7
8
9
dOCR induction
2
5
6

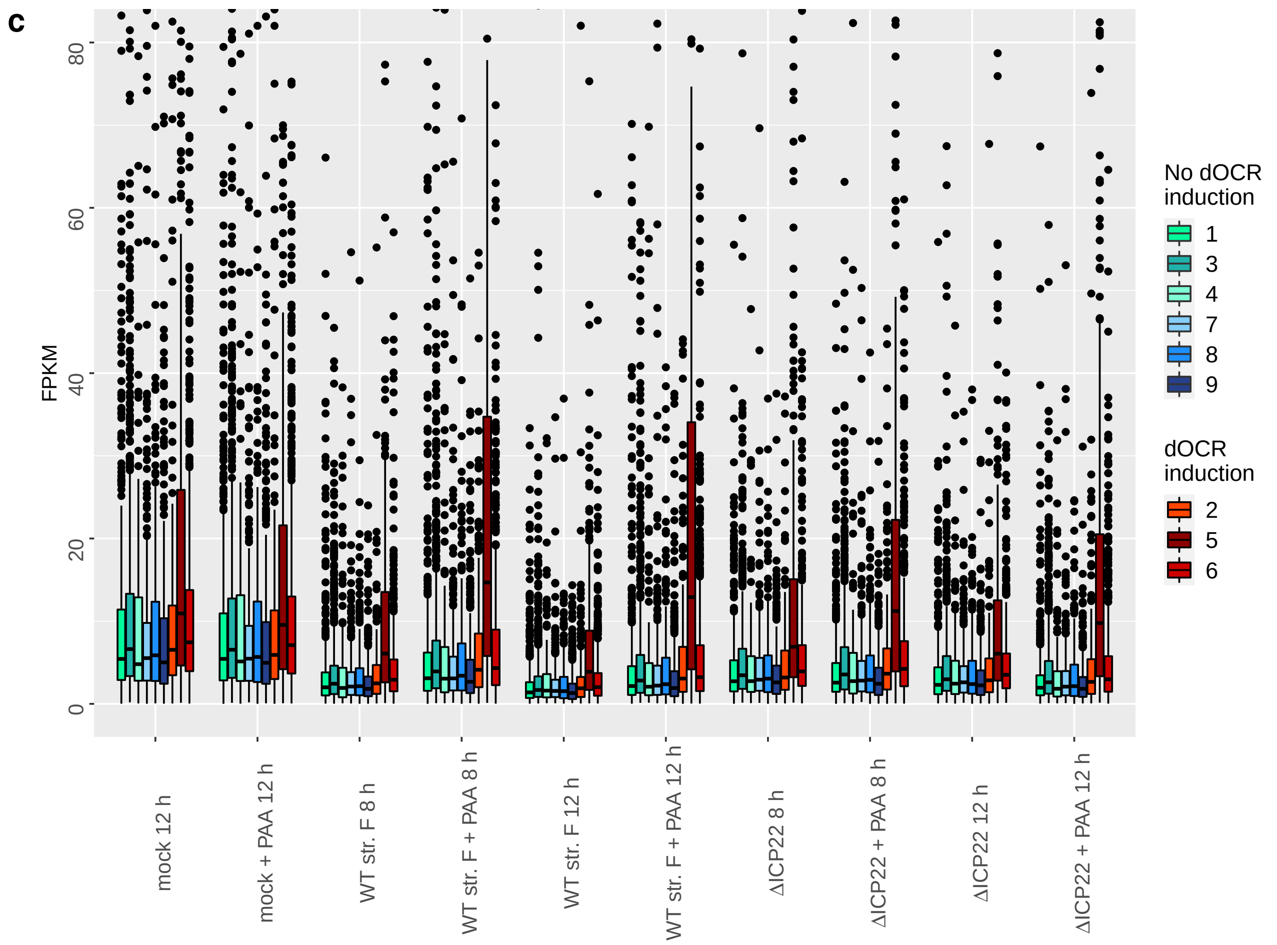

Sup. Fig. 2: Boxplots showing the distribution of **(a)** the percentage of read-through transcription, **(b)** downstream FPKM and **(c)** gene expression (FPKM) for genes in the 9 clusters (n = 609, 290, 851, 176, 305, 701, 367, 289, and 574 genes for clusters 1-9, respectively) in Fig. 1b in total RNA-seq data from the parallel RNA- and ATAC-seq experiments for mock, WT strain F and ΔICP22 infection at 8 and 12 h p.i. $\pm$ PAA treatment. Bounds of boxes are the first and third quartiles for each condition. The center (median) is shown by the horizontal line in the box. Whiskers extend to 1.5 times the inter-quartile range. Outliers are shown as small circles and minimum and maximum values are lowest and highest circles, respectively. Read-through was calculated as described in Methods with mock 12 h p.i. used as reference. Statistical significance for median values being higher for each cluster compared to genes in all other clusters was determined using one-sided Wilcoxon rank sum tests. Resulting p-values are given in the following:

(a)

Cl.	mock 12 h	mock + PAA 12 h	WT str. F + 8 h	WT str. F + PAA 8 h	WT str. F + 12 h	WT str. F + PAA 12 h	ΔICP22 8 h	ΔICP22 + PAA 8 h	ΔICP22 12 h	ΔICP22 + PAA 12 h
1	0.0066	0.024	0.19	0.41	0.24	0.33	0.31	0.39	0.35	0.47
2	0.24	0.052	0.00012	2.2×10^{-6}	0.00081	2.3×10^{-5}	0.0004	2.5×10^{-7}	0.00079	2×10^{-5}
3	0.36	0.62	0.97	1	0.92	1	1	0.99	0.99	1
4	0.92	0.69	0.94	1	1	1	0.64	0.95	0.76	0.95
5	0.98	0.99	1.7×10^{-6}	3.7×10^{-27}	1×10^{-6}	5.2×10^{-24}	0.34	6.3×10^{-5}	0.011	7.4×10^{-10}
6	1	1	1	0.9	1	0.93	0.95	0.97	0.93	0.6
7	0.046	0.05	0.67	1	0.97	1	0.32	0.9	0.53	0.95
8	0.55	0.42	0.32	0.61	0.2	0.4	0.35	0.4	0.54	0.6
9	0.075	0.046	0.84	0.99	0.024	0.96	0.12	0.54	0.38	0.96

(b)

Cl.	mock 12 h	mock + PAA 12 h	WT str. F + 8 h	WT str. F + PAA 8 h	WT str. F + 12 h	WT str. F + PAA 12 h	ΔICP22 8 h	ΔICP22 + PAA 8 h	ΔICP22 12 h	ΔICP22 + PAA 12 h
1	0.11	0.23	0.99	1	0.96	1	0.98	1	0.99	1
2	0.22	0.12	1.9×10^{-5}	3.2×10^{-8}	4.7×10^{-5}	3.1×10^{-8}	0.00076	8.3×10^{-8}	0.00044	2×10^{-6}
3	0.073	0.21	0.96	0.98	0.95	0.99	0.92	0.94	0.9	0.97
4	0.96	0.9	1	1	1	1	0.89	1	0.95	1
5	0.0001	0.00011	3.4×10^{-47}	6.9×10^{-81}	6.5×10^{-40}	7.1×10^{-85}	6.5×10^{-16}	1.1×10^{-40}	1.7×10^{-20}	4.1×10^{-58}
6	0.83	0.9	0.39	0.018	0.66	0.0089	0.14	0.032	0.16	0.0057
7	0.9	0.79	0.97	1	1	1	0.78	1	0.86	1
8	0.95	0.86	0.85	0.94	0.72	0.92	0.91	0.96	0.94	0.96
9	0.98	0.96	1	1	1	1	1	1	1	1

(c)

Cl.	mock 12 h	mock + PAA 12 h	WT str. F + 8 h	WT str. F + PAA 8 h	WT str. F + 12 h	WT str. F + PAA 12 h	ΔICP22 8 h	ΔICP22 + PAA 8 h	ΔICP22 12 h	ΔICP22 + PAA 12 h
1	1	0.99	1	1	1	1	1	1	1	1
2	0.58	0.79	0.36	0.17	0.16	0.055	0.6	0.31	0.46	0.18
3	0.041	0.013	0.42	0.25	0.5	0.34	0.04	0.032	0.061	0.11
4	0.83	0.9	0.97	0.98	0.65	0.98	0.94	0.99	0.9	1
5	1.5×10^{-13}	2.9×10^{-12}	7.4×10^{-41}	2.8×10^{-62}	2.6×10^{-31}	6.8×10^{-66}	8.5×10^{-31}	1×10^{-51}	4.6×10^{-29}	2.2×10^{-57}
6	5.3×10^{-5}	0.00014	1.2×10^{-6}	5.2×10^{-5}	3.7×10^{-5}	5.4×10^{-5}	2.8×10^{-7}	1.4×10^{-9}	3.2×10^{-6}	3.5×10^{-6}
7	1	1	0.99	1	0.9	1	0.99	1	0.98	1
8	0.87	0.85	0.96	0.97	0.95	0.99	0.98	0.99	0.98	0.98
9	1	1	1	1	1	1	1	1	1	1

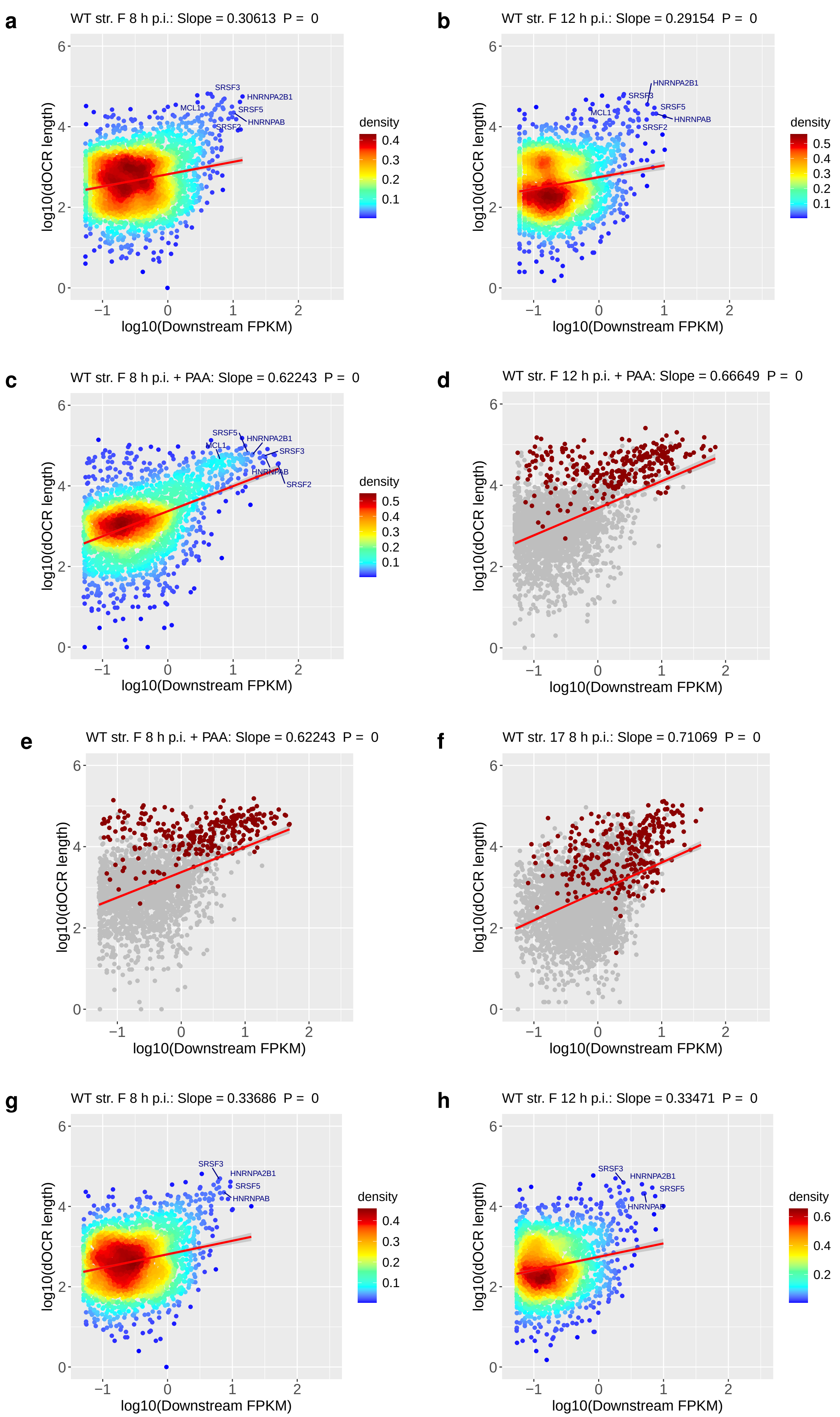
a
WT str. F 8 h p.i.: Slope = 0.30613 P = 0
SRSF3
HNRNPA2B1
MCL1
SRSF5
HNRNPAB
SRSF2
density
log10(dOCR length)
log10(Downstream FPKM)
b
WT str. F 12 h p.i.: Slope = 0.29154 P = 0
HNRNPA2B1
SRSF3
MCL1
SRSF5
HNRNPAB
SRSF2
density
log10(dOCR length)
log10(Downstream FPKM)
c
WT str. F 8 h p.i. + PAA: Slope = 0.62243 P = 0
SRSF5
HNRNPA2B1
MCL1
SRSF3
HNRNPAB
SRSF2
density
log10(dOCR length)
log10(Downstream FPKM)
d
WT str. F 12 h p.i. + PAA: Slope = 0.66649 P = 0
log10(dOCR length)
log10(Downstream FPKM)
e
WT str. F 8 h p.i. + PAA: Slope = 0.62243 P = 0
log10(dOCR length)
log10(Downstream FPKM)
f
WT str. 17 8 h p.i.: Slope = 0.71069 P = 0
log10(dOCR length)
log10(Downstream FPKM)
g
WT str. F 8 h p.i.: Slope = 0.33686 P = 0
SRSF3
HNRNPA2B1
SRSF5
HNRNPAB
density
log10(dOCR length)
log10(Downstream FPKM)
h
WT str. F 12 h p.i.: Slope = 0.33471 P = 0
SRSF3
HNRNPA2B1
SRSF5
HNRNPAB
density
log10(dOCR length)
log10(Downstream FPKM)

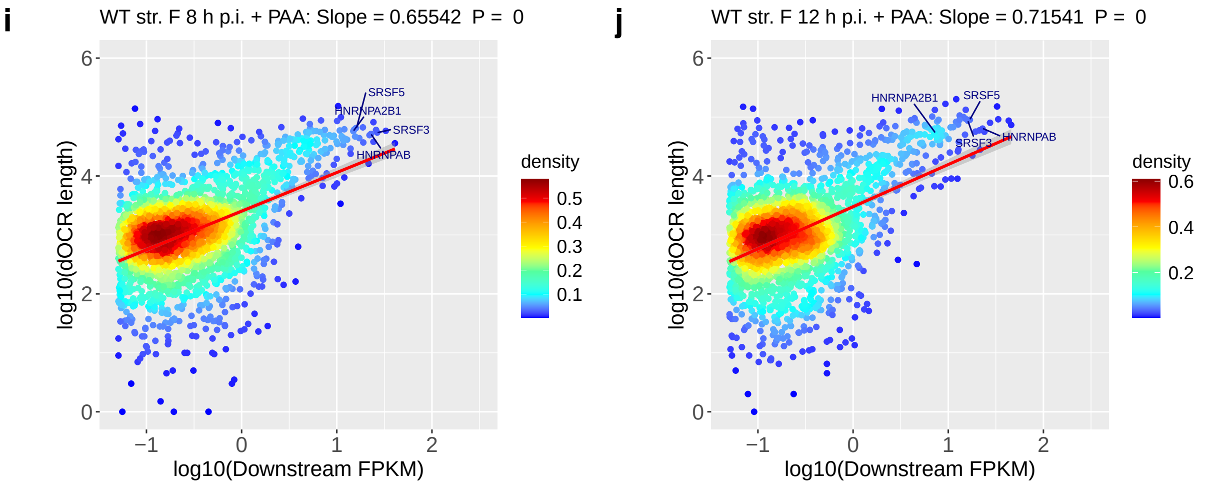

Sup. Fig. 3: (a-c) Scatter plots correlating downstream FPKM in total RNA against dOCR length (average of two replicates) for all analyzed genes with a downstream FPKM $\geq$ 0.05 for WT strain F $\pm$ PAA. Scatter plots for 12 h p.i. WT strain F infection + PAA are shown in Fig. 2a. The red line indicates a linear fit of log10(dOCR length) against log10(downstream FPKM). Colors indicate density of points from high (red) to low (blue). The slope of the fit and p-values for the slope of the linear regression estimate being $\neq 0$ (two-sided test) were calculated using the *lm* function in R and are indicated on top of each figure. The error bands around the red line indicate the 95% confidence level interval for predictions from the *lm* linear model. Example genes with strong induction of dOCRs in HSV-1 infection are highlighted. **(d-f)** Scatter plots correlating downstream FPKM in total RNA **(d,e)** or 4sU-RNA **(f)** against dOCR length (average of two replicates) with genes from Cluster 5 colored red. All other analyzed genes are colored gray. **(g-j)** Scatter plots as in **(a-c)** and Fig. 2a with downstream FPKM calculated in 10 kb windows downstream of genes instead of 5 kb windows.

a RNA-seq WT str. F 8 h p.i. + PAA

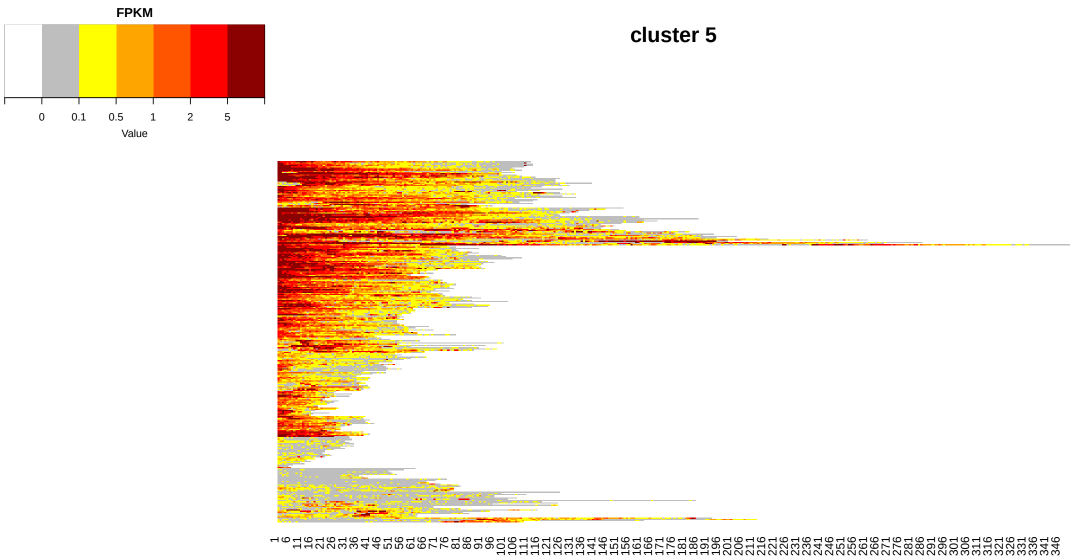

b RNA-seq WT str. F 12 h p.i. + PAA

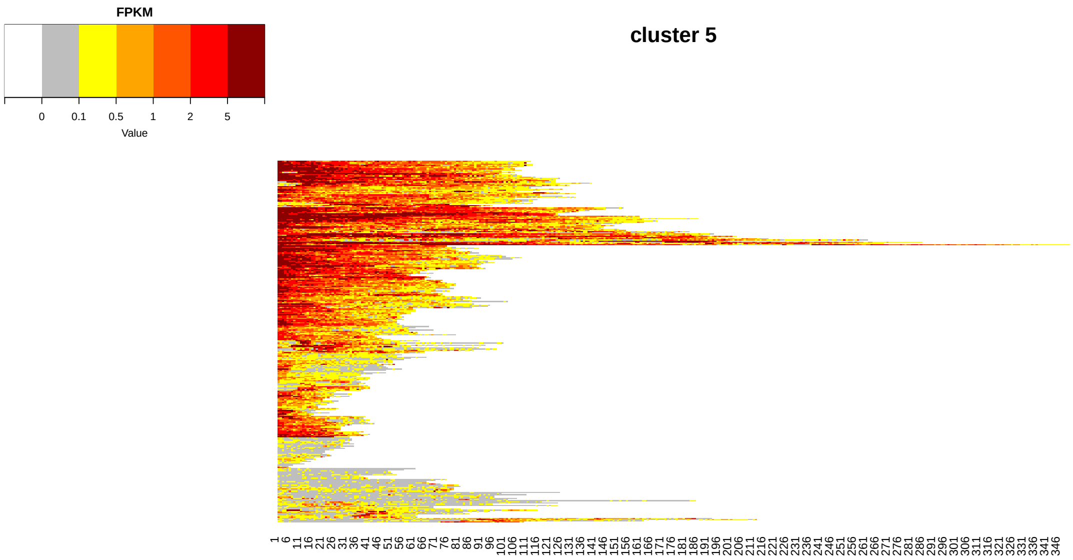

c RNA-seq WT str. F 8 h p.i.

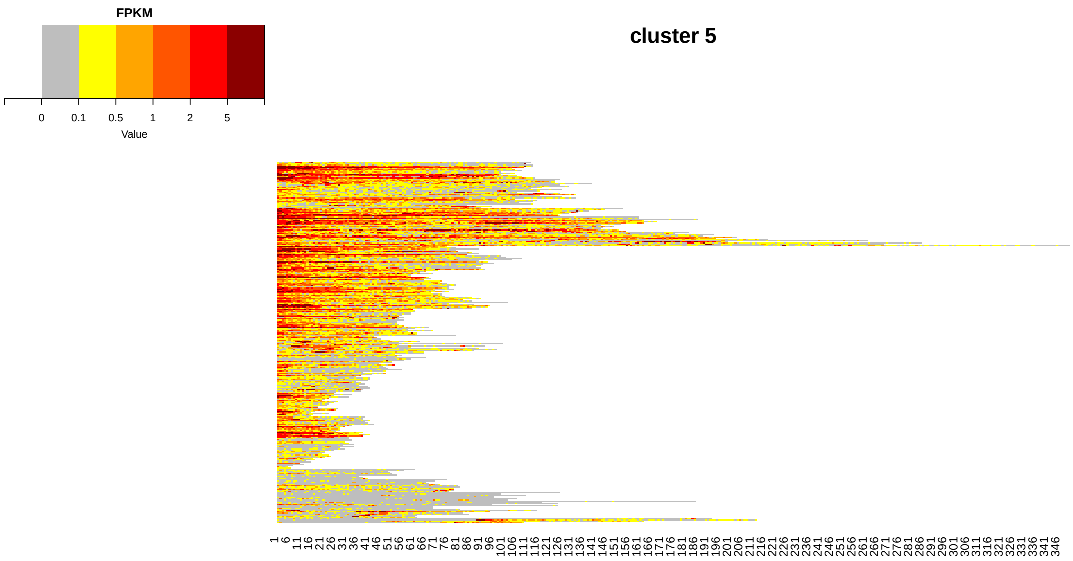

d RNA-seq WT str. F 12 h p.i.

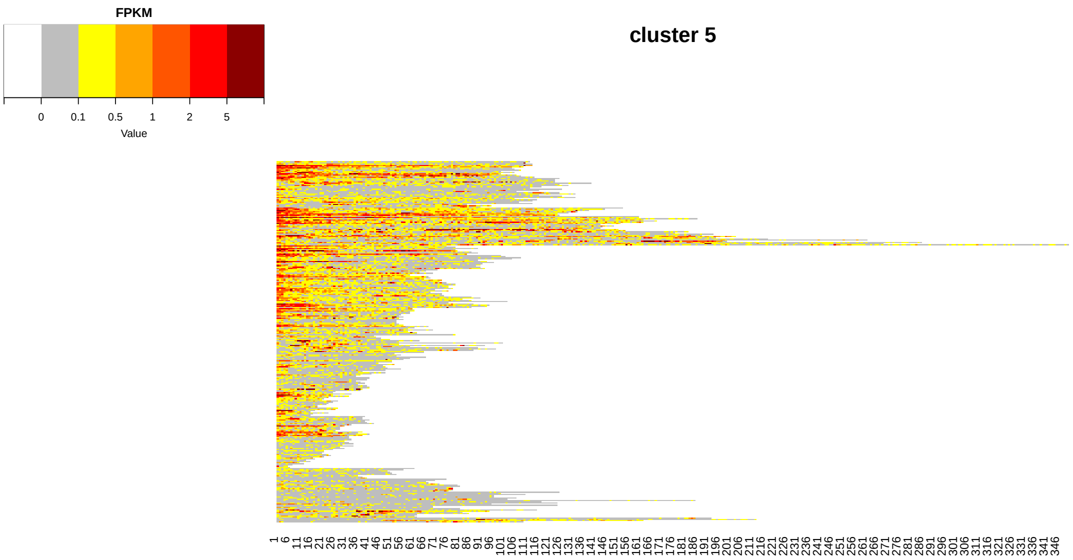

e RNA-seq mock

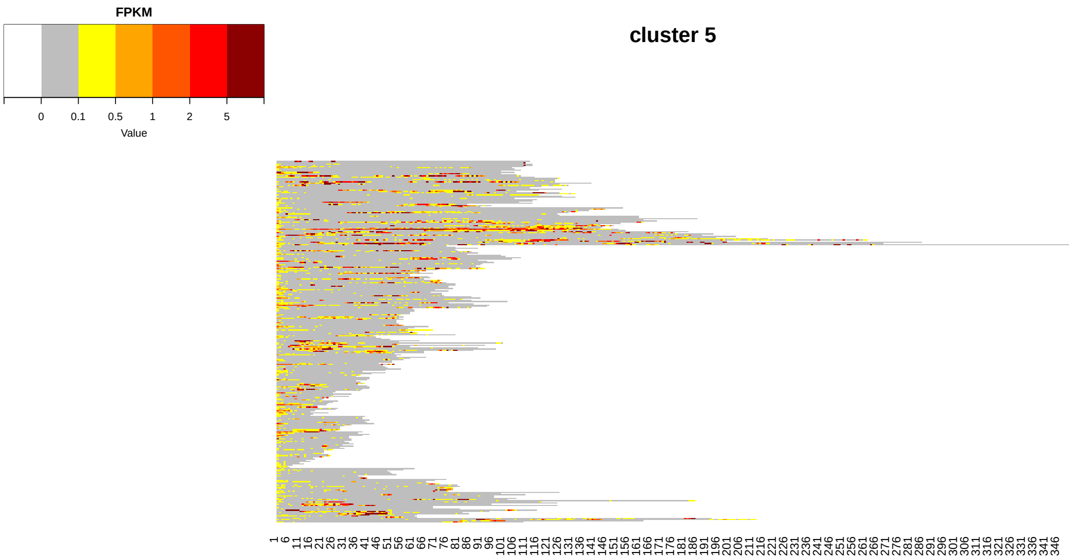

f ATAC-seq WT str. F 12 h p.i. + PAA

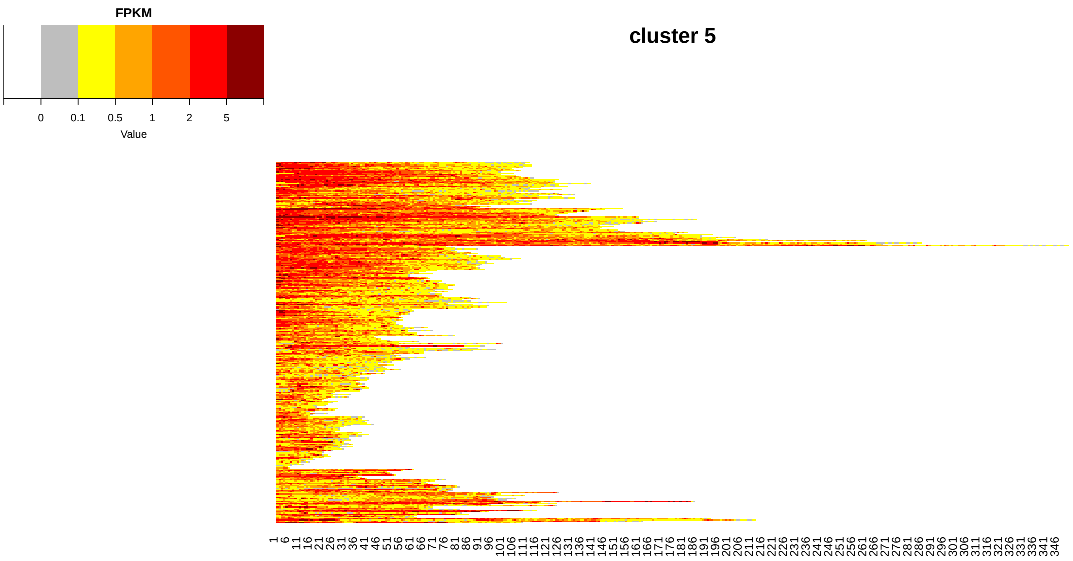

Sup. Fig. 4: Figures show RNA-seq **(a-e)** and ATAC-seq read coverage **(f)** on dOCR regions that were determined for Cluster 5 genes in WT strain F 12 h p.i. infection with PAA treatment. Panels **(a-e)** show RNA-seq coverage in infection with **(a)** WT strain F 8 h p.i. with PAA treatment, **(b)** WT strain F 12 h p.i. with PAA treatment, **(c)** WT strain F 8 h p.i. , **(d)** WT strain F 12 h p.i. and **(e)** mock. Panel **(f)** shows ATAC-seq read coverage in WT strain F 12 h p.i. infection with PAA treatment. Each row in each figure corresponds to one gene in Cluster 5 with dOCR length > 0 in WT strain F 12 h p.i. infection with PAA treatment. Numbers at the bottom indicate the genomic distance from the gene 3'end. dOCR regions for a gene are colored according to FPKM values for 1kb windows as shown in the legend, while regions downstream of dOCRs for each gene are white. Genes were clustered according to Euclidean distances on ATAC-seq FPKM values in 1kb windows on dOCR regions for WT strain F 12 h p.i. infection with PAA treatment using Ward's clustering criterion and have the same order in each subfigure.

a RNA-seq WT str. F 8 h p.i. + PAA

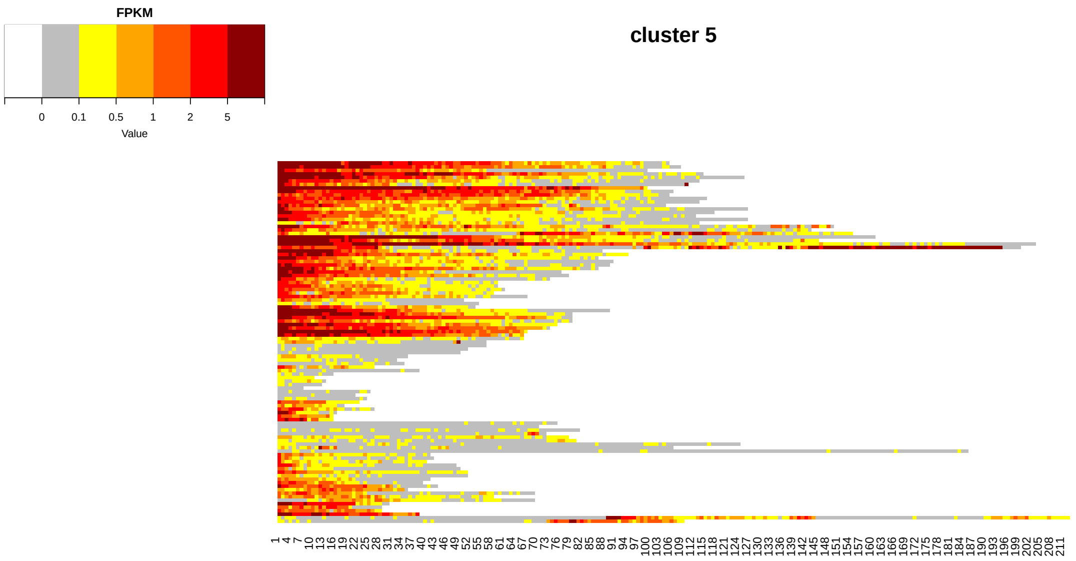

b RNA-seq WT str. F 12 h p.i. + PAA

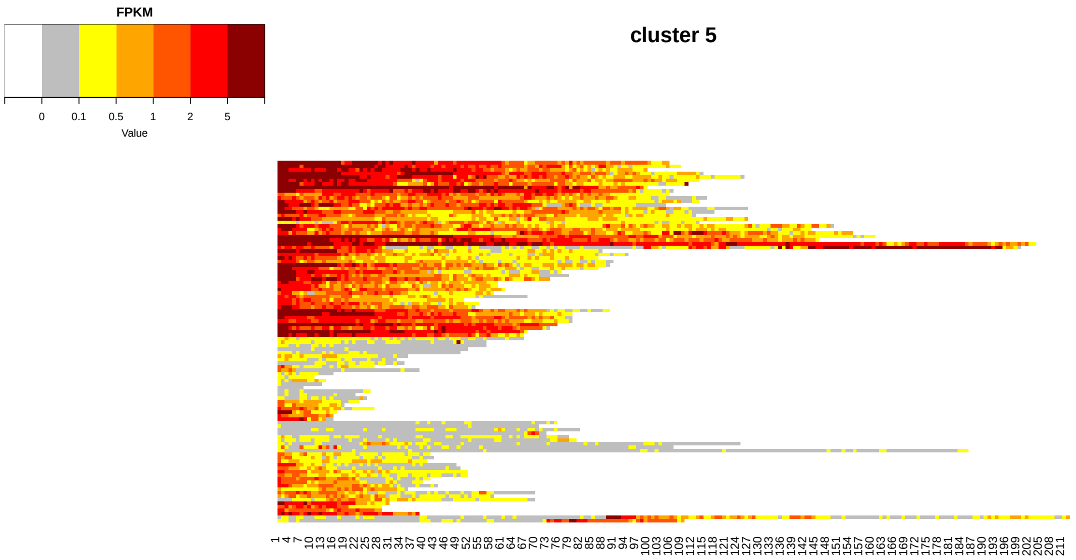

c RNA-seq WT str. F 8 h p.i.

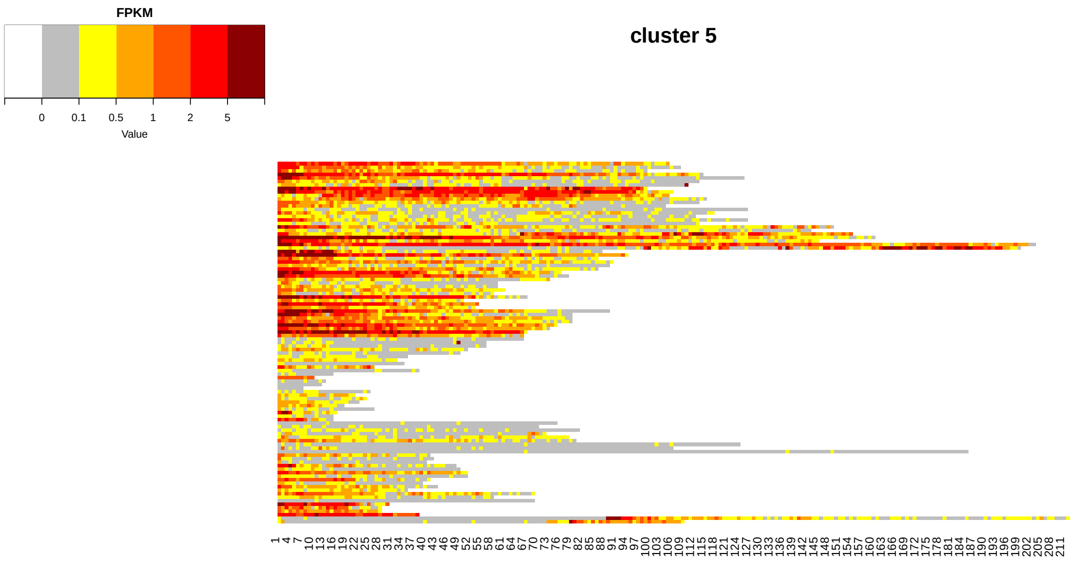

d RNA-seq WT str. F 12 h p.i.

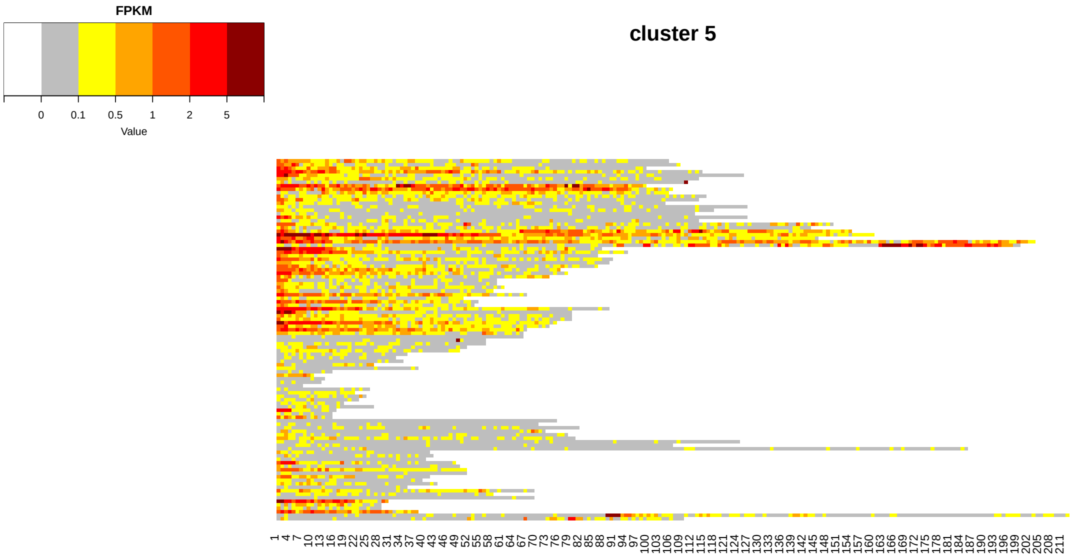

e RNA-seq mock

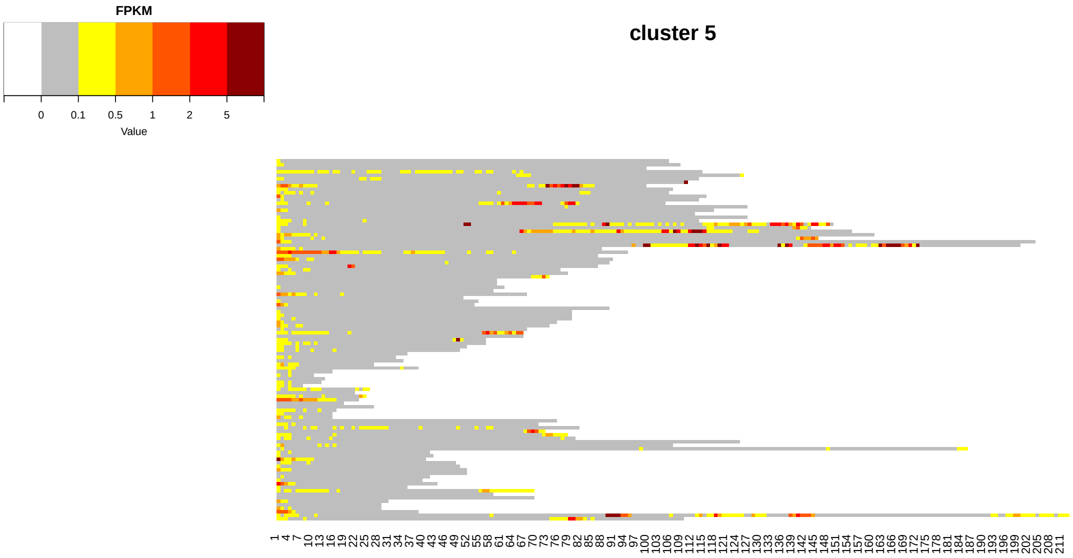

f ATAC-seq WT str. F 12 h p.i. + PAA

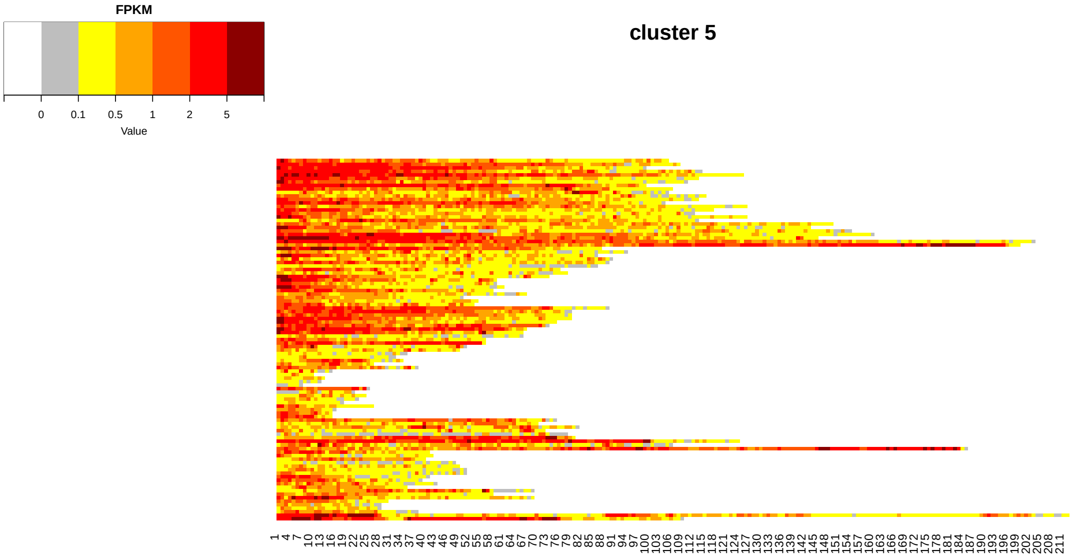

Sup. Fig. 5: Figures as in Sup. Fig. 4 restricted to genes without known protein-coding or lincRNA genes within 50kb downstream of their 3'end (=103 genes in Cluster 5) according to the Ensembl annotation (see Methods). Panels **(a-e)** show RNA-seq coverage in infection with **(a)** WT strain F 8 h p.i. with PAA treatment, **(b)** WT strain F 12 h p.i. with PAA treatment, **(c)** WT strain F 8 h p.i. , **(d)** WT strain F 12 h p.i. and **(e)** mock. Panel **(f)** shows ATAC-seq read coverage in WT strain F 12 h p.i. infection with PAA treatment.

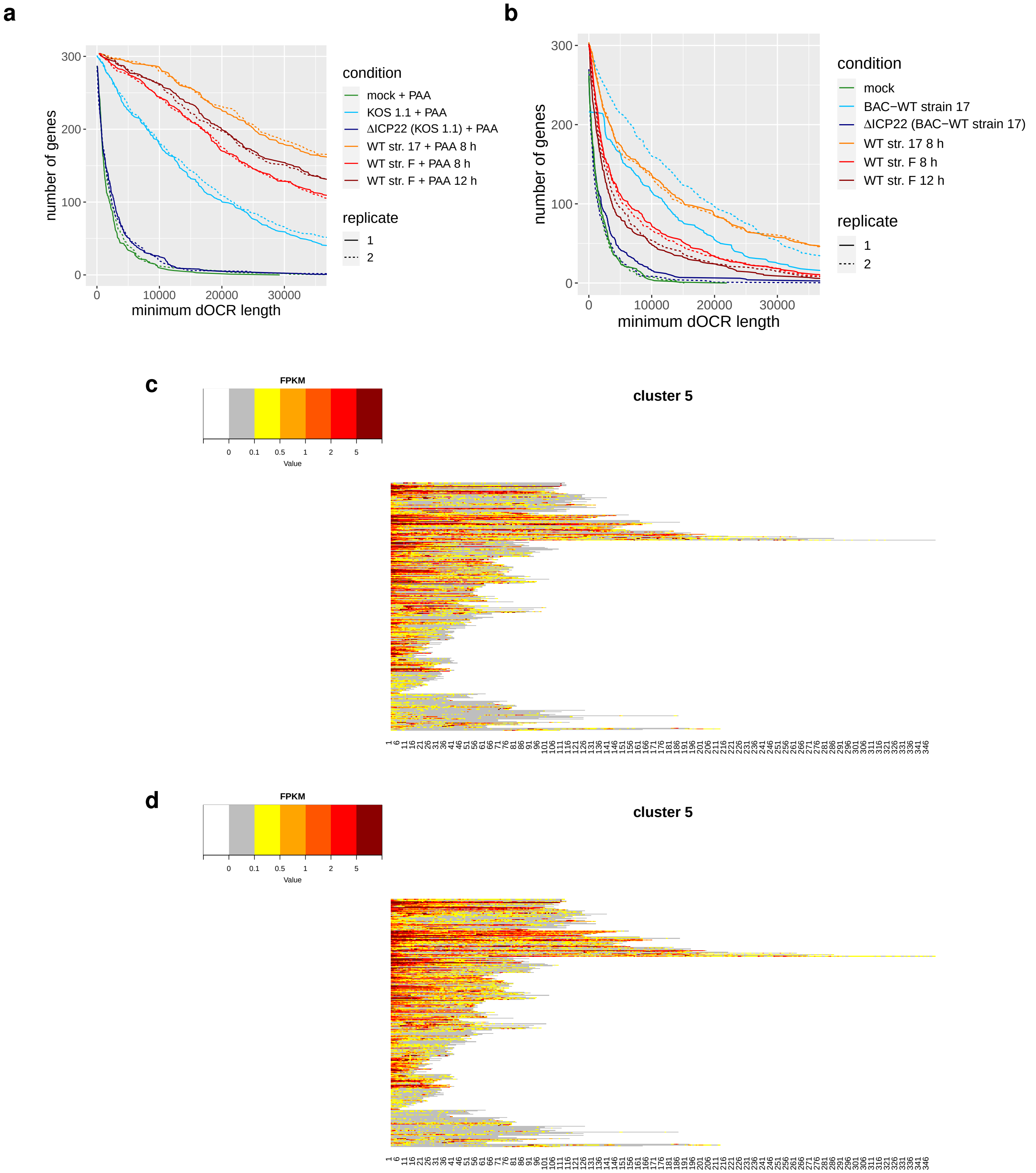

Sup. Fig. 6: (a-b) Number of genes in Cluster 5 (y-axis) for which dOCRs reach a length greater than the value indicated on the x-axis in **(a)** mock, WT strain KOS1.1 infection and infection with a ΔICP22 mutant derived from KOS1.1 (all with PAA treatment) and **(b)** mock, WT strain 17 (BAC-derived) infection and infection with a ΔICP22 mutant derived from BAC-WT strain 17 (no PAA treatment). For each condition, these figures include all Cluster 5 genes with a dOCR length > 0 for that particular condition. For comparison, corresponding curves for WT strain 17 and F infection are also included (with PAA in **(a)** and without PAA in **(b)**). **(c-d)** Figures as in Sup. Fig. 4 showing RNA-seq read coverage in **(c)** 8 h and **(d)** 12 h p.i. ΔICP22 infection + PAA on dOCR regions that were identified for 12 h p.i. WT strain F infection + PAA. For a description of this type of figures see caption to Sup. Fig. 4.

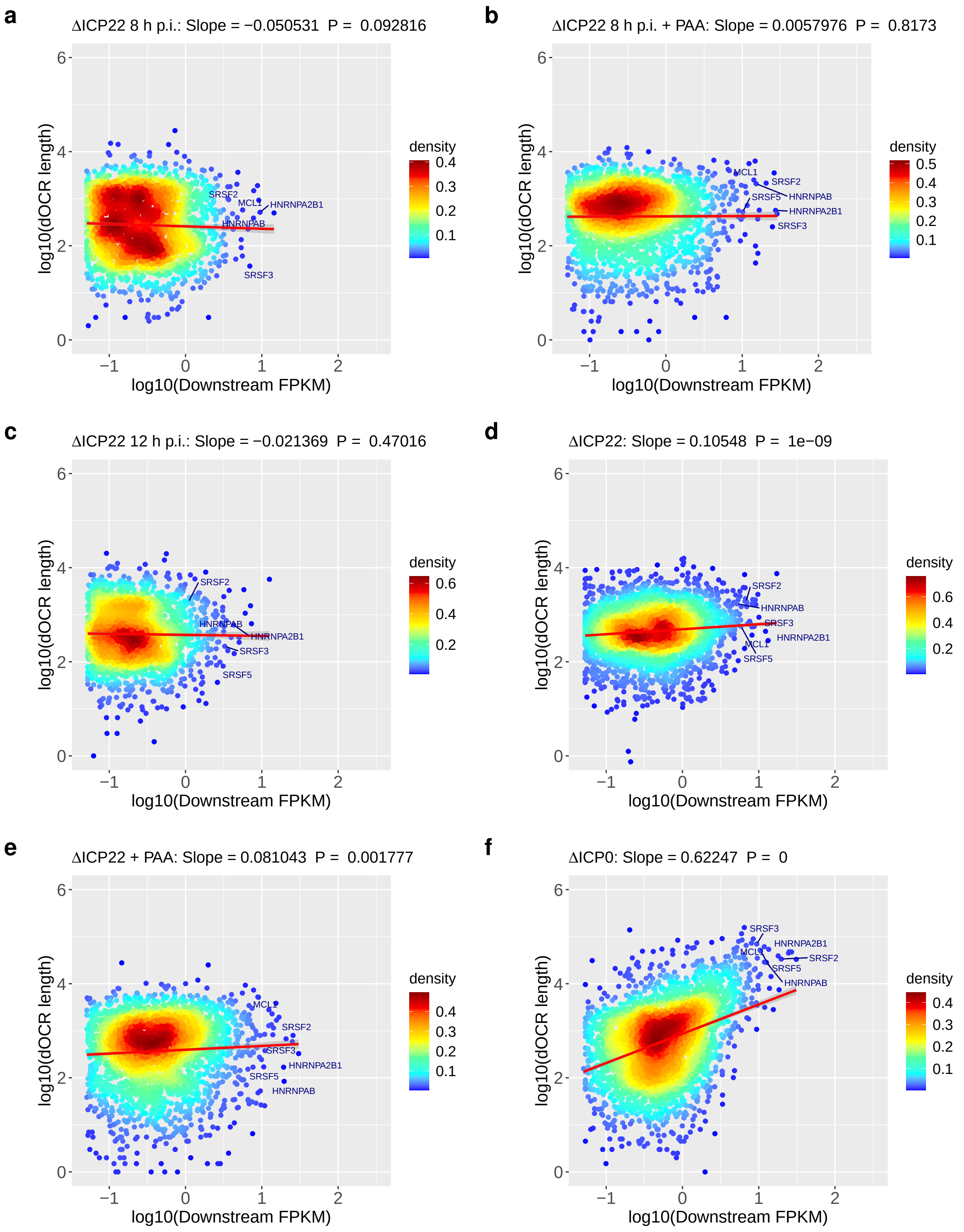
a
ΔICP22 8 h p.i.: Slope = −0.050531 P = 0.092816
log10(dOCR length)
log10(Downstream FPKM)
density
SRSF2
MCL1
HNRNPA2B1
HNRNPAB
SRSF3
b
ΔICP22 8 h p.i. + PAA: Slope = 0.0057976 P = 0.8173
log10(dOCR length)
log10(Downstream FPKM)
density
MCL1
SRSF2
SRSF5
HNRNPAB
HNRNPA2B1
SRSF3
c
ΔICP22 12 h p.i.: Slope = −0.021369 P = 0.47016
log10(dOCR length)
log10(Downstream FPKM)
density
SRSF2
HNRNPAB
HNRNPA2B1
SRSF3
SRSF5
d
ΔICP22: Slope = 0.10548 P = 1e−09
log10(dOCR length)
log10(Downstream FPKM)
density
SRSF2
HNRNPAB
SRSF3
HNRNPA2B1
MCL1
SRSF5
e
ΔICP22 + PAA: Slope = 0.081043 P = 0.001777
log10(dOCR length)
log10(Downstream FPKM)
density
MCL1
SRSF2
SRSF3
HNRNPA2B1
SRSF5
HNRNPAB
f
ΔICP0: Slope = 0.62247 P = 0
log10(dOCR length)
log10(Downstream FPKM)
density
SRSF3
HNRNPA2B1
MCL1
SRSF2
SRSF5
HNRNPAB

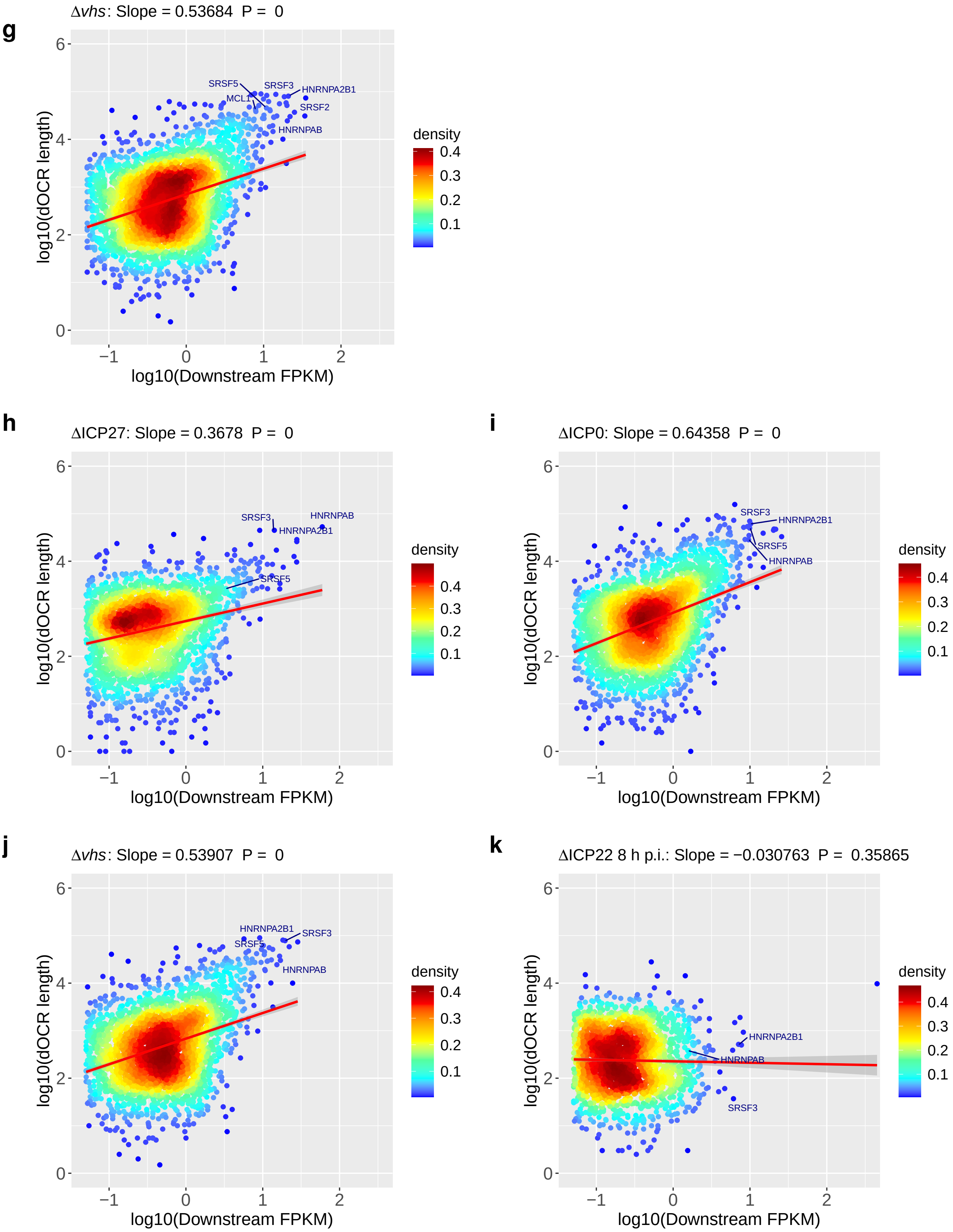
g
Δvhs: Slope = 0.53684 P = 0
SRSF5
SRSF3
HNRNPA2B1
MCL1
SRSF2
HNRNPAB
log10(dOCR length)
log10(Downstream FPKM)
density
0.4
0.3
0.2
0.1
h
ΔICP27: Slope = 0.3678 P = 0
SRSF3
HNRNPAB
HNRNPA2B1
SRSF5
log10(dOCR length)
log10(Downstream FPKM)
density
i
ΔICP0: Slope = 0.64358 P = 0
SRSF3
HNRNPA2B1
SRSF5
HNRNPAB
log10(dOCR length)
log10(Downstream FPKM)
density
j
Δvhs: Slope = 0.53907 P = 0
HNRNPA2B1
SRSF3
SRSF5
HNRNPAB
log10(dOCR length)
log10(Downstream FPKM)
density
k
ΔICP22 8 h p.i.: Slope = −0.030763 P = 0.35865
HNRNPA2B1
HNRNPAB
SRSF3
log10(dOCR length)
log10(Downstream FPKM)
density

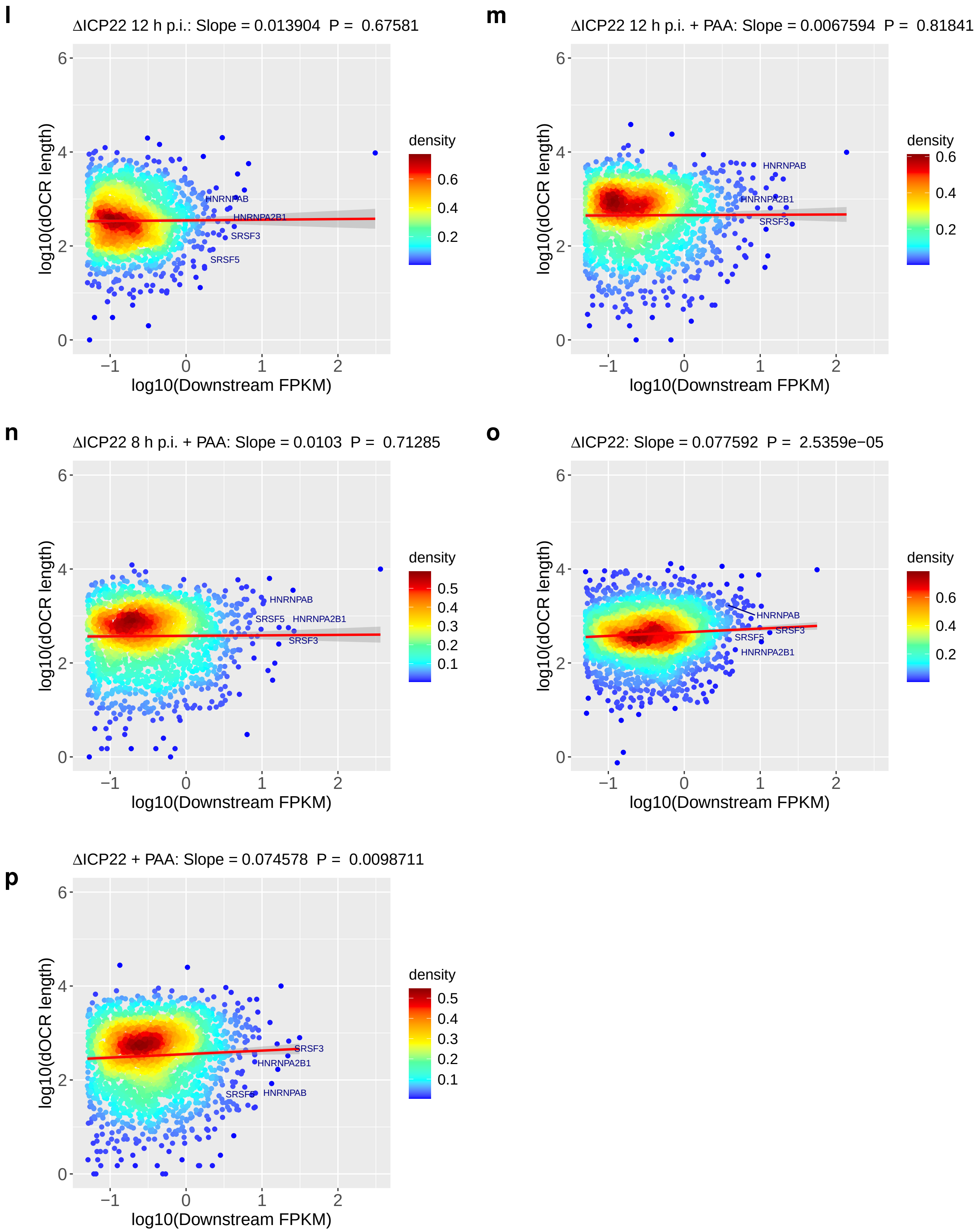

Sup. Fig. 7: (a-g) Scatter plots correlating downstream FPKM in total RNA **(a-c)** or 4sU-RNA **(d-g)** against dOCR length (average of two replicates) for all analyzed genes with a downstream FPKM $\geq$ 0.05. The red line indicates a linear fit of log10(dOCR length) against log10(downstream FPKM). Colors indicate density of points from high (red) to low (blue). The slope of the fit and p-values for the slope of the linear regression estimate being $\neq 0$ (two-sided test) were calculated using the *lm* function in R and are indicated on top of each figure. The error bands around the red line indicate the 95% confidence level interval for predictions from the *lm* linear model. Example genes with strong induction of dOCRs in HSV-1 infection are highlighted. **(h-p)** Scatter plots as in Fig. 2 e,f and **(a-g)** with downstream FPKM calculated in 10 kb windows downstream of genes instead of 5 kb windows.

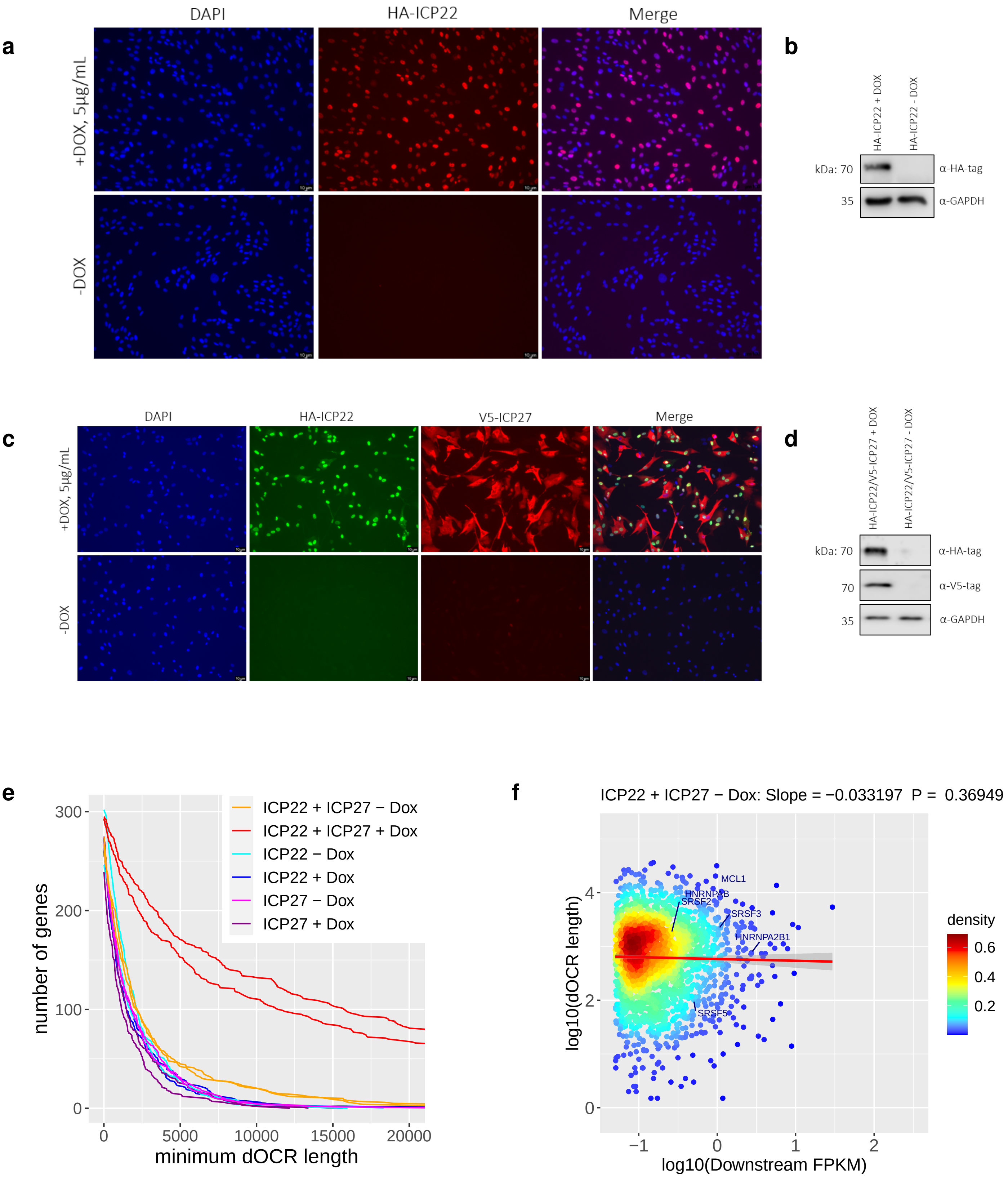
a
DAPI
HA-ICP22
Merge
+DOX, 5µg/mL
-DOX
b
HA-ICP22 + DOX
HA-ICP22 - DOX
kDa: 70
35
α-HA-tag
α-GAPDH
c
DAPI
HA-ICP22
V5-ICP27
Merge
+DOX, 5µg/mL
-DOX
d
HA-ICP22/V5-ICP27 + DOX
HA-ICP22/V5-ICP27 - DOX
kDa: 70
70
35
α-HA-tag
α-V5-tag
α-GAPDH
e
ICP22 + ICP27 − Dox
ICP22 + ICP27 + Dox
ICP22 − Dox
ICP22 + Dox
ICP27 − Dox
ICP27 + Dox
number of genes
minimum dOCR length
0
100
200
300
5000
10000
15000
20000
f
ICP22 + ICP27 − Dox: Slope = −0.033197 P = 0.36949
log10(dOCR length)
log10(Downstream FPKM)
MCL1
HNRNPAB
SRSF2
SRSF3
HNRNPA2B1
SRSF5
density
0.6
0.4
0.2
−1
0
1
2
4

g

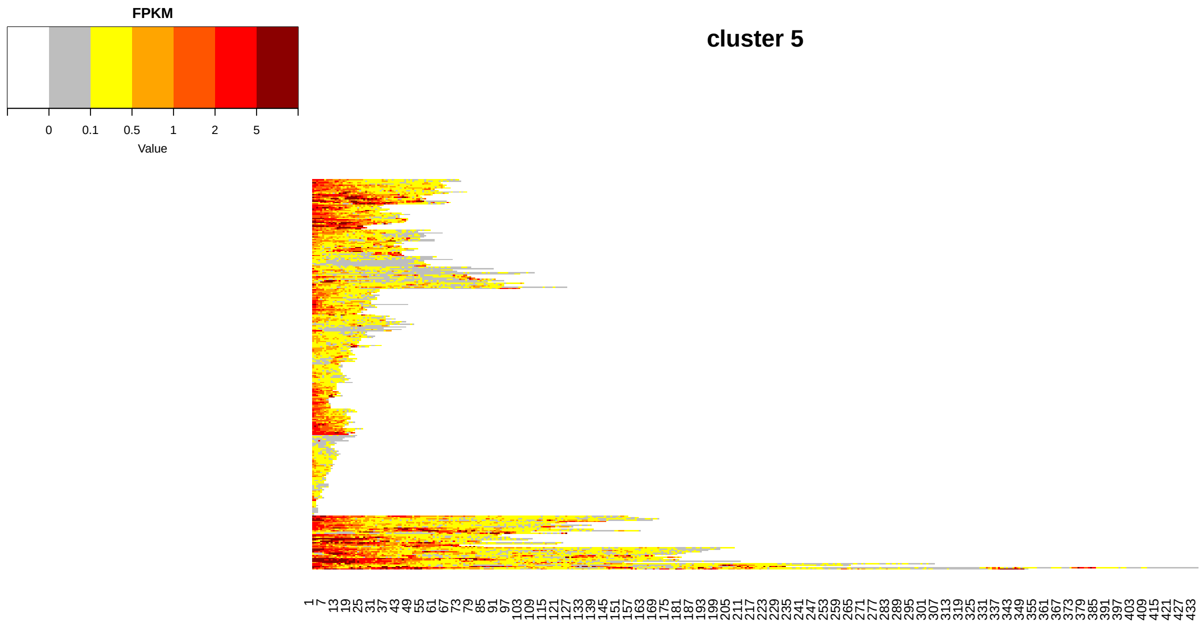

h

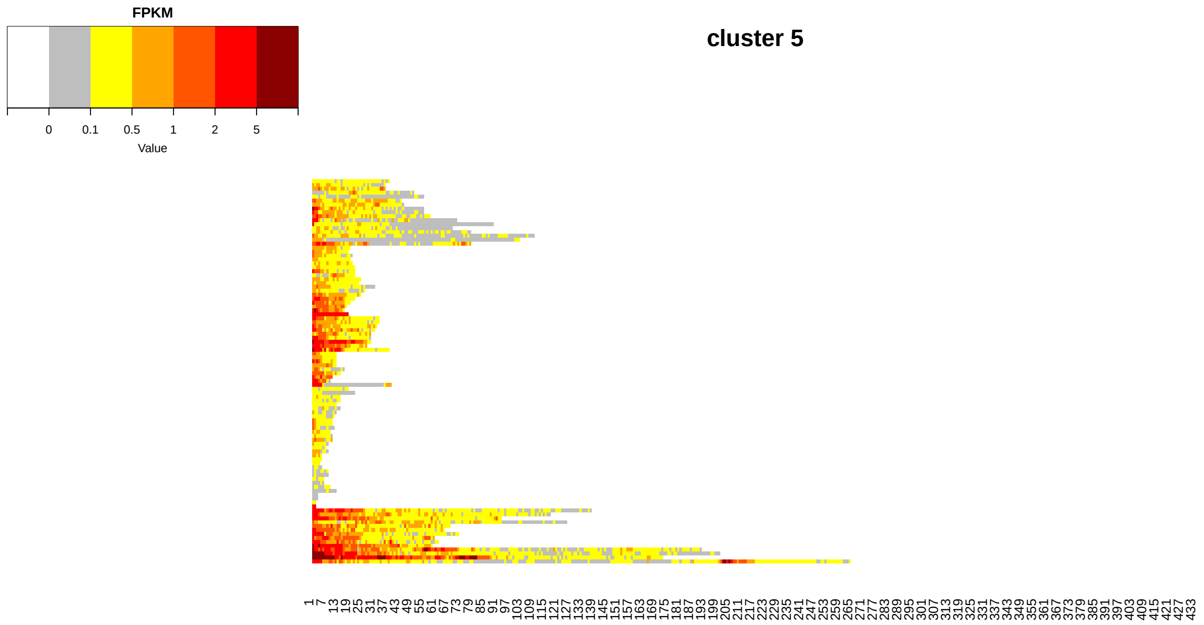

Sup. Fig. 8: (a-b) Generation of polyclonal HA-ICP22 doxycycline-inducible cells. T-HFs transduced with inducible HA-ICP22-expressing lentivirus were seeded in presence or absence of 5μg/mL doxycycline (Dox) and collected 48h post induction. Upon addition of Dox, cells express HA-ICP22. Immunofluorescence and western blot images were obtained from the day of ATAC/Omni-ATAC-seq experiment. **(a)** HA-ICP22 protein is shown in red and DAPI depicts cell nuclei. Mean value of cells expressing HA-ICP22 was calculated from there different fields of the same experiment and is ~88%. **(b)** Total cell lysates were collected 48h post Dox-induction and were probed for HA-tag. GAPDH was used as a loading control. **(c-d)** Generation of polyclonal HA-ICP22+V5-ICP27 Dox-inducible cells. V5-ICP27 Dox-inducible polyclonal cells transduced with inducible HA-ICP22-expressing lentivirus were seeded in presence or absence of 5μg/mL Dox and collected 48h post induction. Upon addition of Dox cells express both HA-ICP22 and V5-ICP27. Immunofluorescence and western blot images were obtained from the day of ATAC/Omni-ATAC-seq experiment. **(c)** HA-ICP22 protein is shown in green, V5-ICP27 is shown in red and DAPI (blue) depicts cell nuclei. Mean value of cells expressing HA-ICP22, V5-ICP27, or HA-ICP22 and V5-ICP27 was calculated from three different fields of the same experiment and is ~80%, 90%, 90% respectively. **(d)** Total cell lysates were collected 48h post Dox-induction and were probed for HA-tag and V5-tag. GAPDH

was used as a loading control. **(a-d)** Source images for immunofluorescence images and for western blot images (without and with ladder and as 'ladder only' scans) are supplied as a Source Data file. Each experiment was repeated independently twice with similar results. **(e)** Number of genes in Cluster 5 (y-axis) for which dOCRs reach a length greater than the value indicated on the x-axis in T-HFs-ICP22/ICP27 cells $\pm$ Dox, T-HFs-ICP22 cells $\pm$ Dox, and T-HFs-ICP27 cells $\pm$ Dox. Results are shown after down-sampling to approximately the same number of reads on the cellular genome. For each condition, this figure include all Cluster 5 genes with a dOCR length > 0 for that particular condition. **(f)** Scatter plot correlating downstream FPKM in total RNA (x-axis) against dOCR length (average of two replicates) for T-HFs-ICP22/ICP27 cells without Dox exposure. Colors indicate density of points from high (red) to low (blue). The slope of the fit and p-values for the slope of the linear regression estimate being $\neq 0$ (two-sided test) were calculated using the *lm* function in R and are indicated on top of each figure. The error bands around the red line indicate the 95% confidence level interval for predictions from the *lm* linear model. Example genes with strong induction of dOCRs in HSV-1 infection are highlighted. **(g-h)** RNA-seq read coverage in T-HFs-ICP22/ICP27 cells with Dox treatment on identified dOCR regions for **(g)** all Cluster 5 genes (=305 genes) and **(h)** Cluster 5 genes without any known protein-coding or lincRNA genes within 50kb downstream of their 3' end (=103 genes). For a description of this type of figures see caption to Sup. Fig. 4.

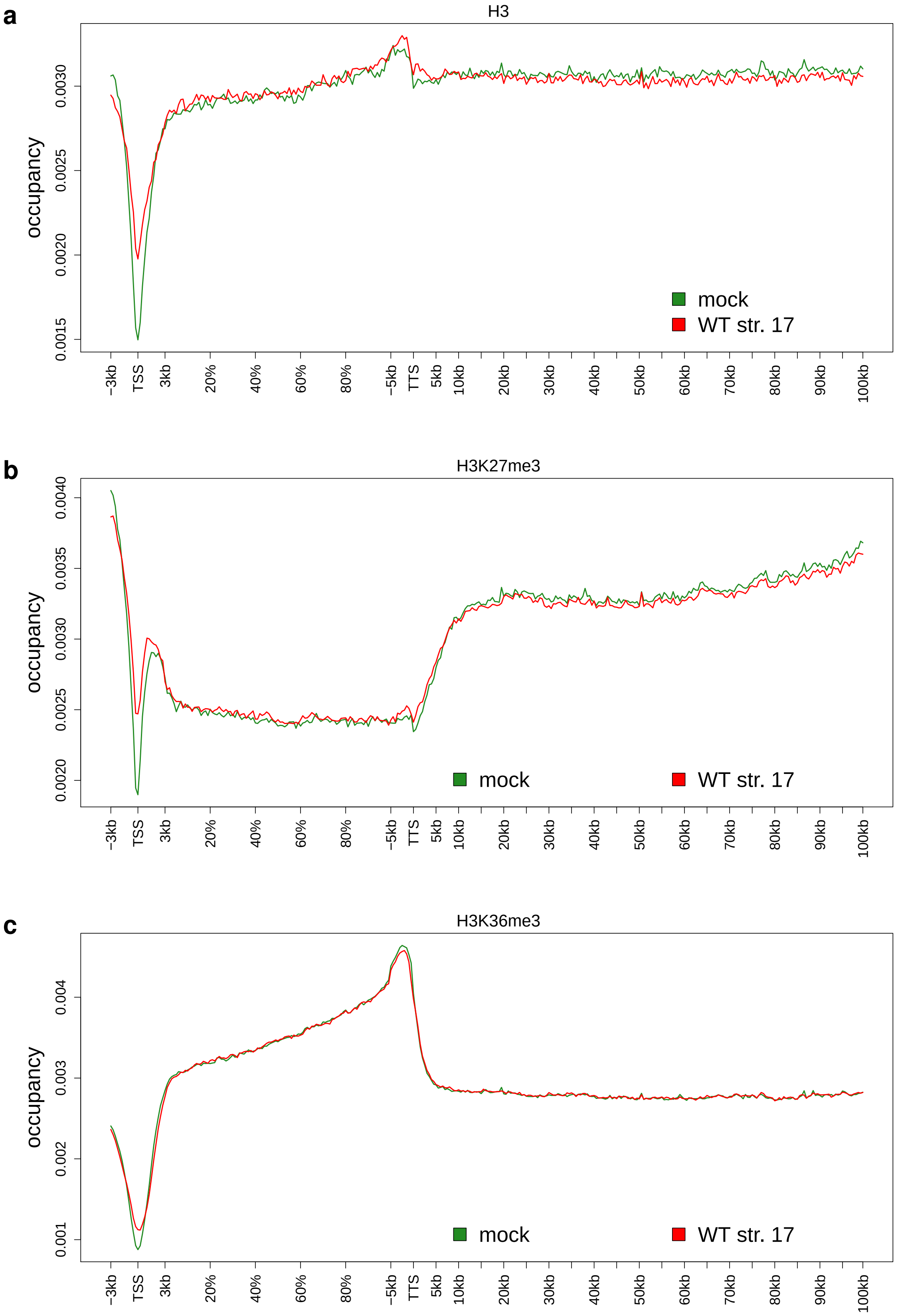
a
H3
occupancy
0.0015
0.0020
0.0025
0.0030
mock
WT str. 17
b
H3K27me3
occupancy
0.0020
0.0025
0.0030
0.0035
0.0040
mock
WT str. 17
c
H3K36me3
occupancy
0.001
0.002
0.003
0.004
mock
WT str. 17
−3kb
TSS
3kb
20%
40%
60%
80%
−5kb
TTS
5kb
10kb
20kb
30kb
40kb
50kb
60kb
70kb
80kb
90kb
100kb

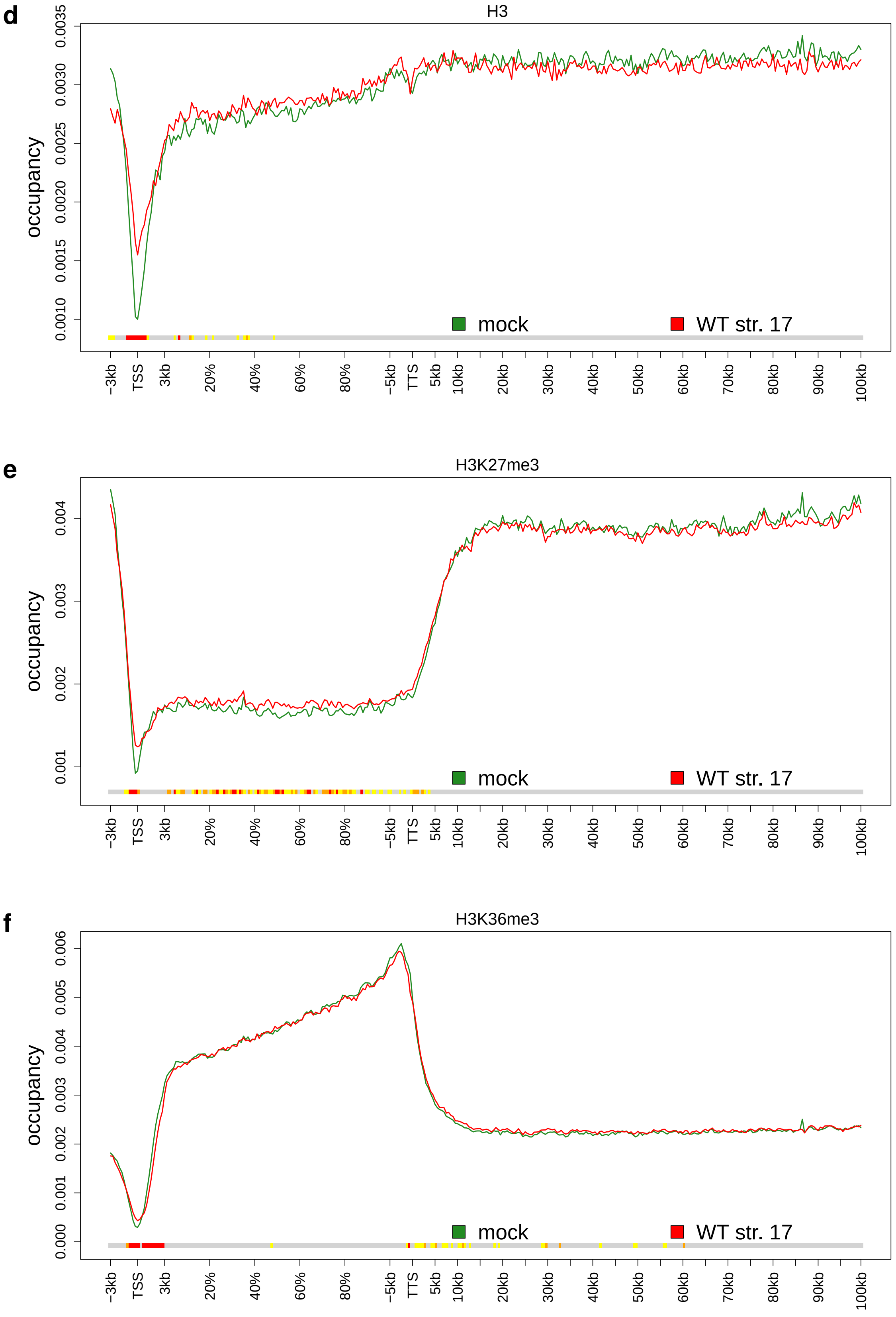

Sup. Fig. 9: Metagene plots showing the occupancy profile of **(a,d)** H3, **(b,e)** H3K27me3 and **(c,f)** H3K36me3 for all annotated protein-coding genes **(a-c)** or genes from clusters without induction of dOCRs in mock and WT strain 17 infection, i.e. all clusters apart from Cluster 2,5 and 6 **(d-f)**. The color track at the bottom of subfigures **(d-f)** indicates the significance of paired two-sided Wilcoxon tests comparing the normalized transcript coverages of genes for each bin between mock and WT infection. P-values are adjusted for multiple testing with the Bonferroni method within each subfigure; color code: red = adj. p-value $\leq 10^{-5}$, orange = adj. p-value $\leq 10^{-3}$, yellow = adj. p-value ≤ 0.05. Exact p-values are included in the source data provided as a Source Data file.

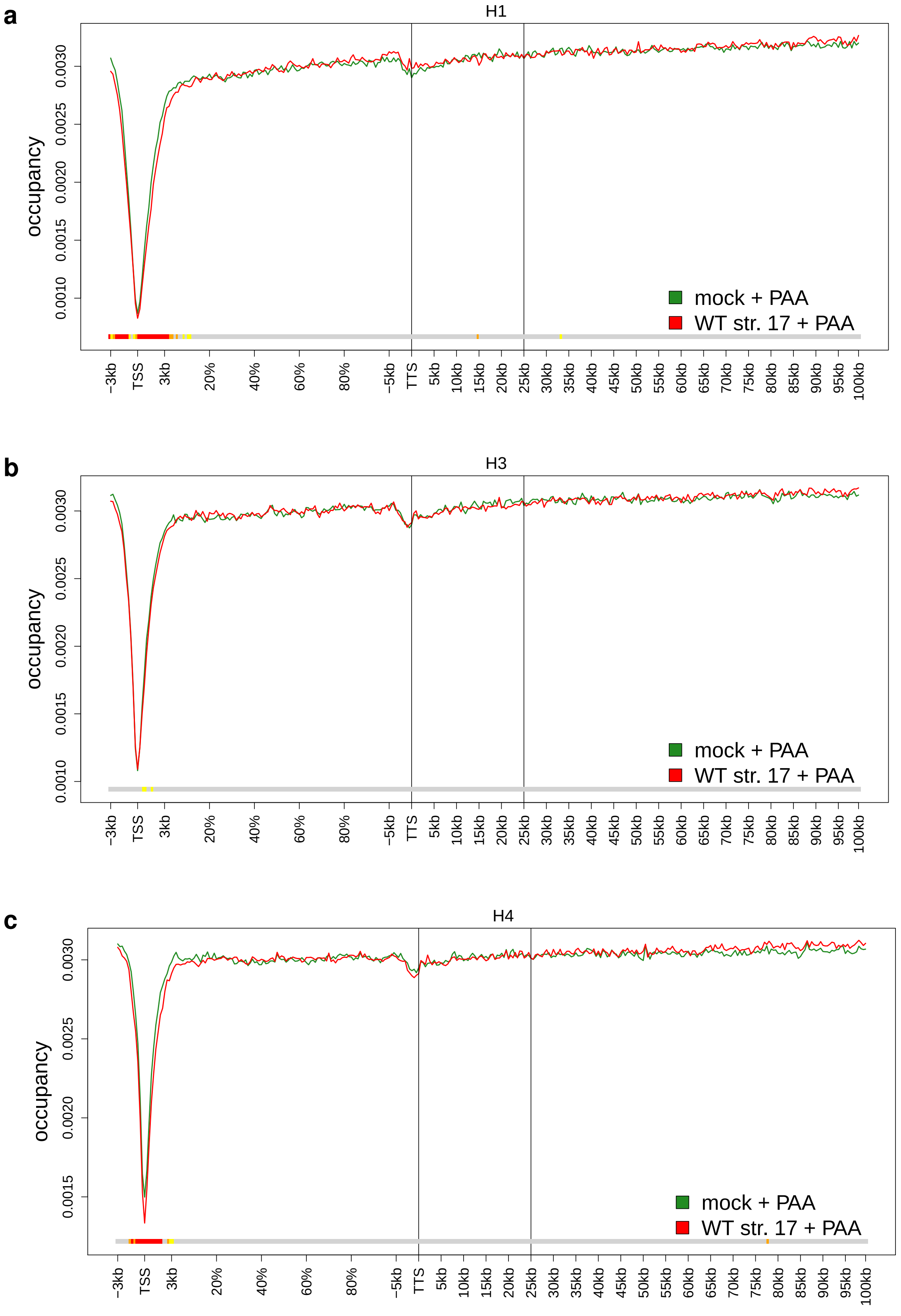
a
H1
occupancy
0.0010
0.0015
0.0020
0.0025
0.0030
mock + PAA
WT str. 17 + PAA
−3kb
TSS
3kb
20%
40%
60%
80%
−5kb
TTS
5kb
10kb
15kb
20kb
25kb
30kb
35kb
40kb
45kb
50kb
55kb
60kb
65kb
70kb
75kb
80kb
85kb
90kb
95kb
100kb
b
H3
c
H4

d

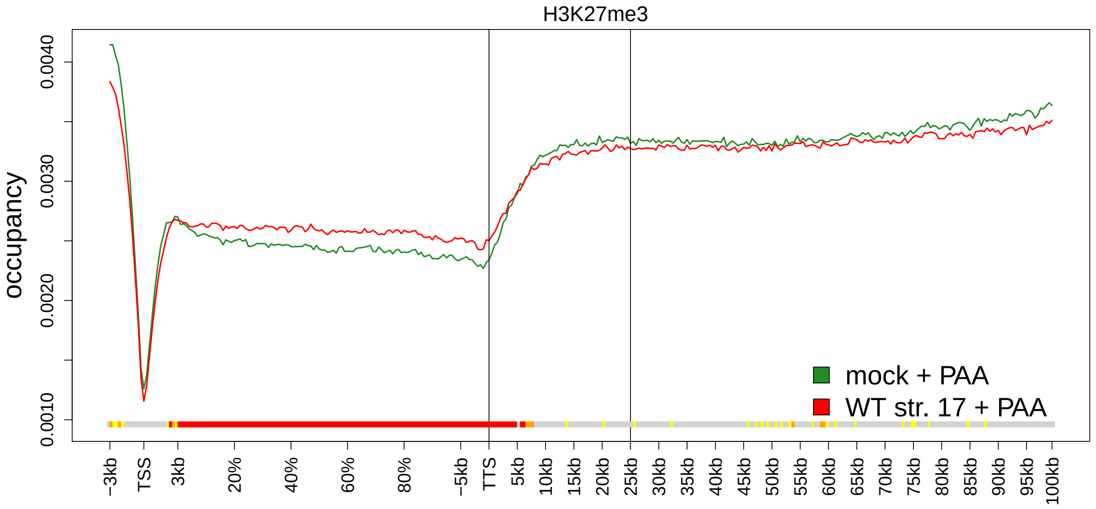

e

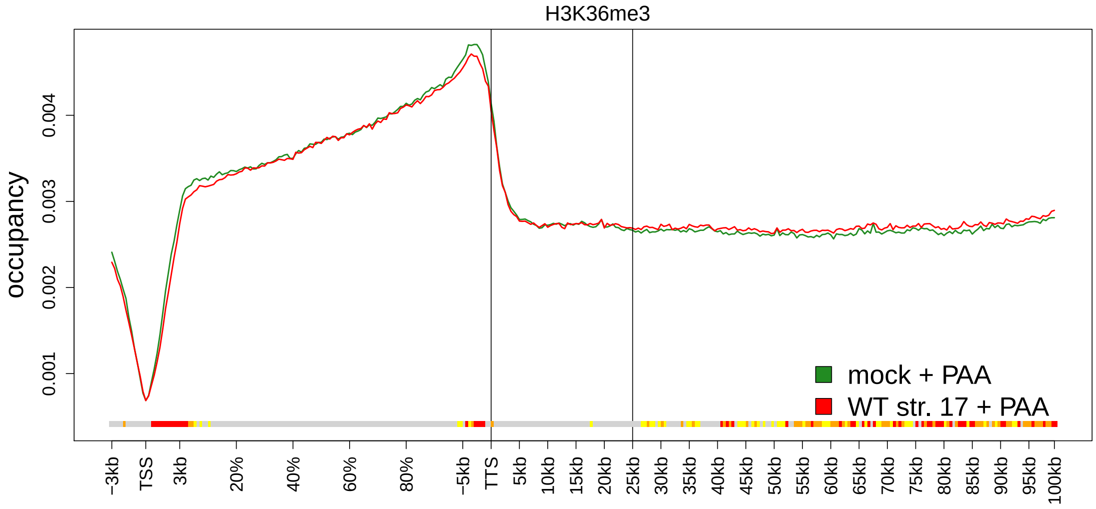

Sup. Fig. 10: (a-e) Metagene plots showing the occupancy profile of **(a)** H1, **(b)** H3, **(c)** H4, **(d)** H3K27me3 and **(e)** H3K36me3 for all annotated protein-coding genes in mock and WT strain 17 infection with PAA treatment. The color track at the bottom of each subfigure indicates the significance of paired two-sided Wilcoxon tests comparing the normalized transcript coverages for each bin between mock and WT infection. P-values are adjusted for multiple testing with the Bonferroni method within each subfigure; color code: red = adj. p-value $\leq 10^{-5}$, orange = adj. p-value $\leq 10^{-3}$, yellow = adj. p-value ≤ 0.05. Exact p-values are included in the source data provided as a Source Data file.

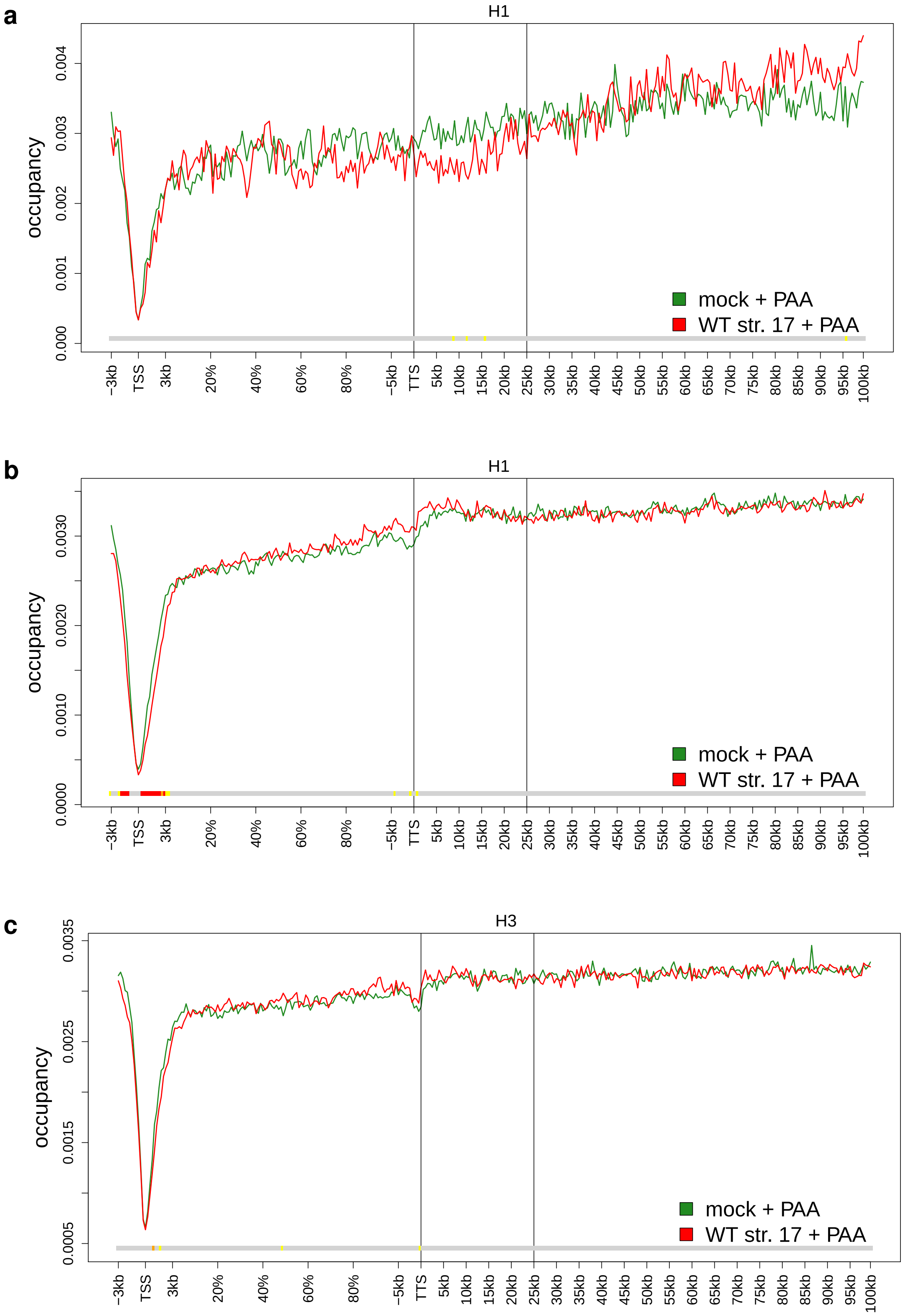
a
H1
occupancy
0.000
0.001
0.002
0.003
0.004
mock + PAA
WT str. 17 + PAA
−3kb
TSS
3kb
20%
40%
60%
80%
−5kb
TTS
5kb
10kb
15kb
20kb
25kb
30kb
35kb
40kb
45kb
50kb
55kb
60kb
65kb
70kb
75kb
80kb
85kb
90kb
95kb
100kb
b
H1
occupancy
0.0000
0.0010
0.0020
0.0030
mock + PAA
WT str. 17 + PAA
c
H3
occupancy
0.0005
0.0015
0.0025
0.0035
mock + PAA
WT str. 17 + PAA

d

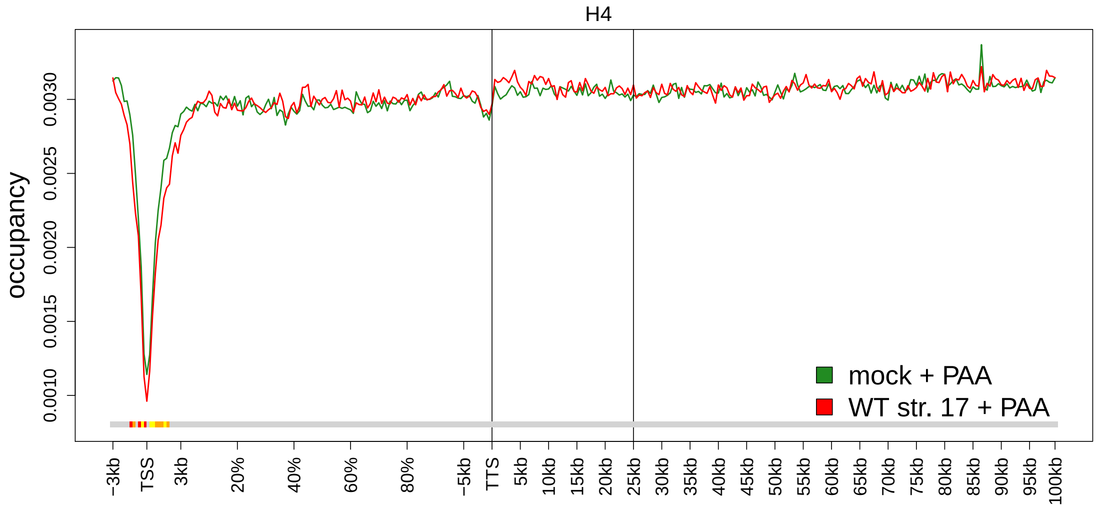
H4
occupancy
0.0010
0.0015
0.0020
0.0025
0.0030
mock + PAA
WT str. 17 + PAA
−3kb
TSS
3kb
20%
40%
60%
80%
−5kb
TTS
5kb
10kb
15kb
20kb
25kb
30kb
35kb
40kb
45kb
50kb
55kb
60kb
65kb
70kb
75kb
80kb
85kb
90kb
95kb
100kb

e

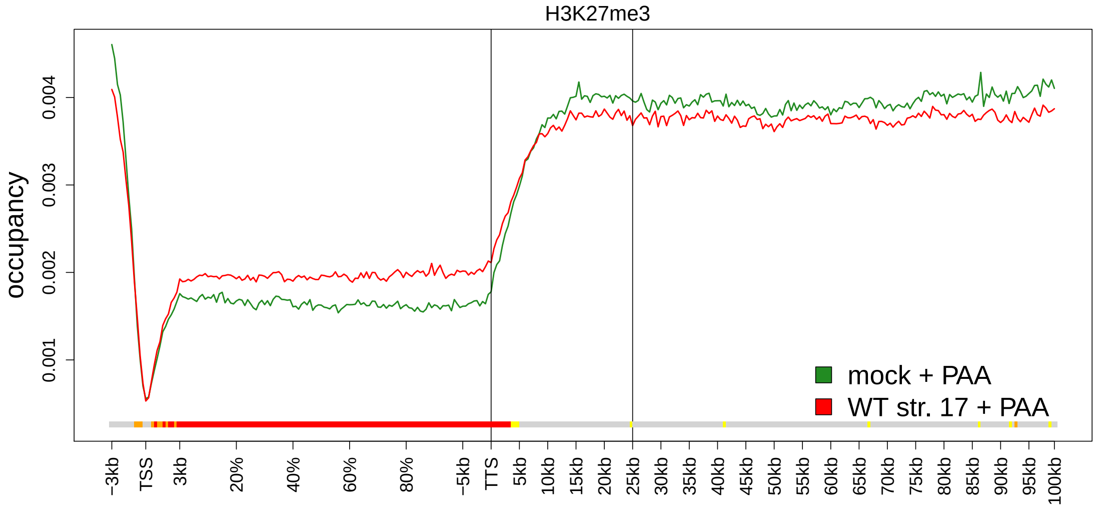
H3K27me3
occupancy
0.001
0.002
0.003
0.004
mock + PAA
WT str. 17 + PAA
−3kb
TSS
3kb
20%
40%
60%
80%
−5kb
TTS
5kb
10kb
15kb
20kb
25kb
30kb
35kb
40kb
45kb
50kb
55kb
60kb
65kb
70kb
75kb
80kb
85kb
90kb
95kb
100kb

f

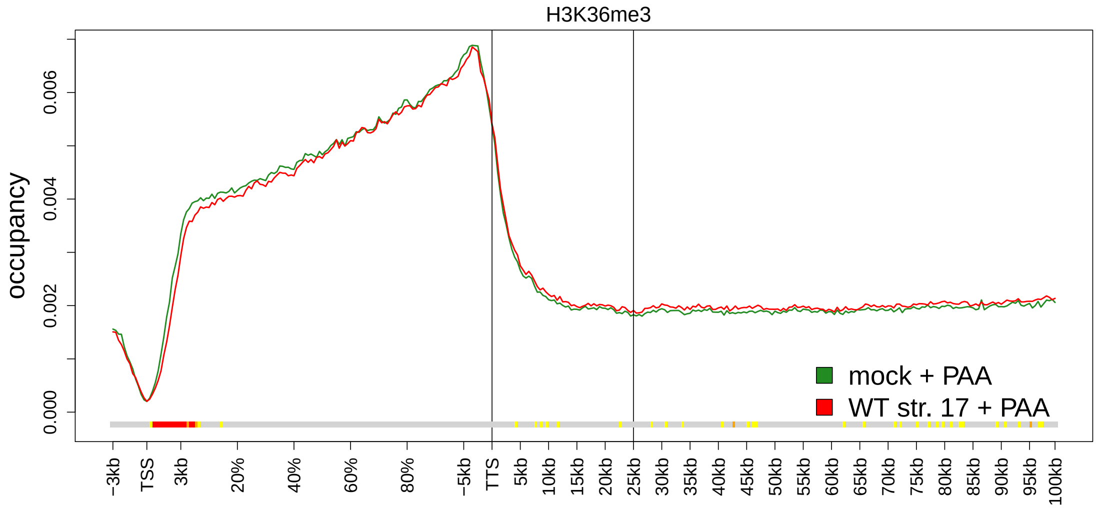
H3K36me3
occupancy
0.000
0.002
0.004
0.006
mock + PAA
WT str. 17 + PAA
−3kb
TSS
3kb
20%
40%
60%
80%
−5kb
TTS
5kb
10kb
15kb
20kb
25kb
30kb
35kb
40kb
45kb
50kb
55kb
60kb
65kb
70kb
75kb
80kb
85kb
90kb
95kb
100kb

g

H1 WT str. 17 + PAA vs. Mock + PAA

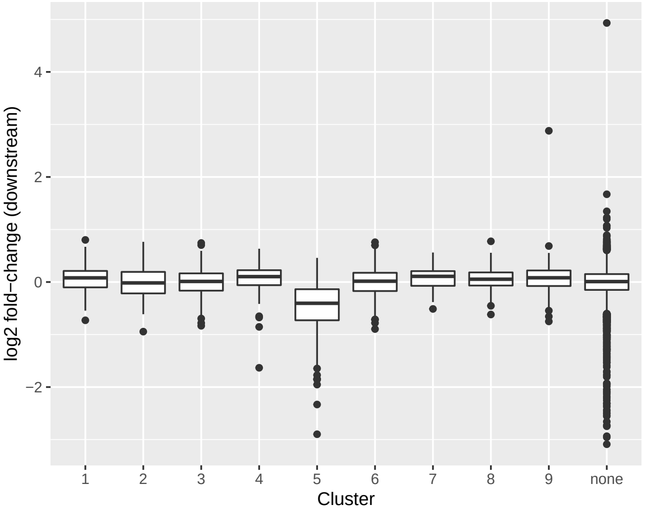

Cluster	1	2	3	4	5	6	7	8	9
p(Cl)	0.2	1	1	0.3	6e−37	1	0.02	1	0.0005
p(All)	0.003	1	1	0.02	4e−34	1	0.0002	1	2e−06

h

H3 WT str. 17 + PAA vs. Mock + PAA

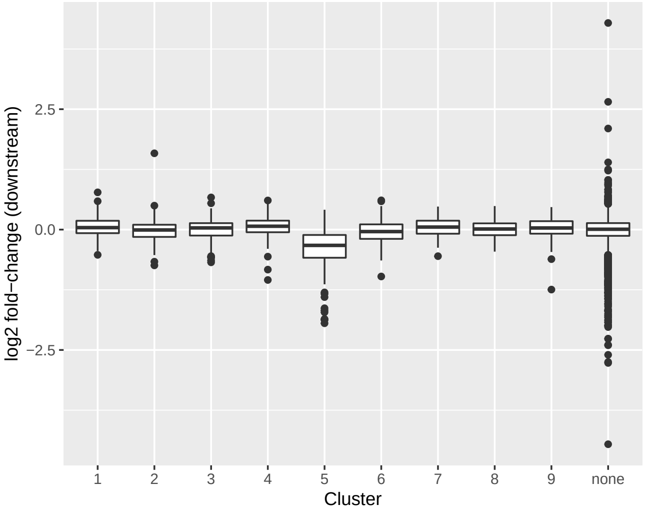

Cluster	1	2	3	4	5	6	7	8	9
p(Cl)	0.0003	1	1	0.3	1e−35	0.009	0.1	1	0.009
p(All)	0.001	1	1	0.3	8e−35	0.01	0.2	1	0.03

i

H1 WT str. 17 + PAA vs. Mock + PAA

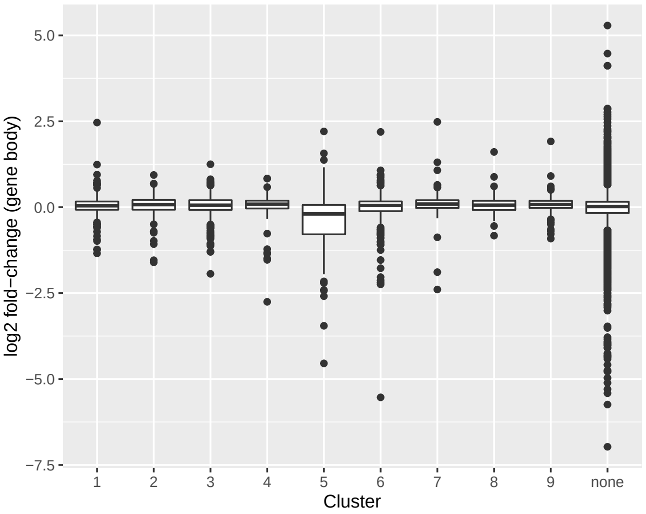

Cluster	1	2	3	4	5	6	7	8	9
p(Cl)	1	1	1	1	3e−15	1	0.4	1	0.3
p(All)	1	1	0.1	0.8	3e−10	1	0.0007	1	7e−05

j

H3 WT str. 17 + PAA vs. Mock + PAA

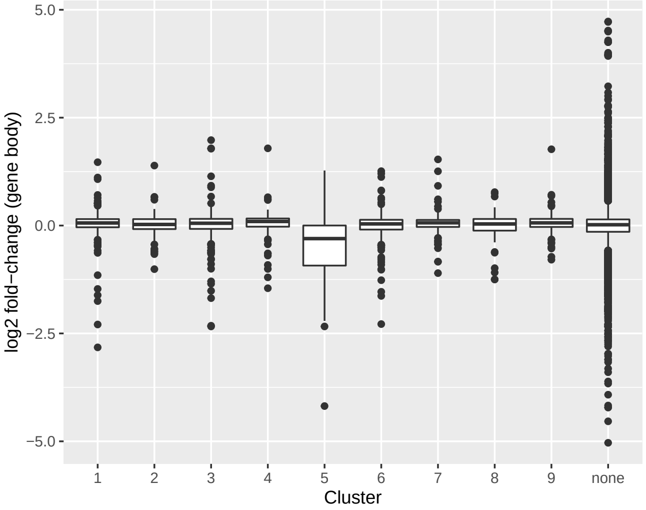

Cluster	1	2	3	4	5	6	7	8	9
p(Cl)	1	1	1	1	5e−26	1	1	1	0.2
p(All)	0.08	1	0.5	0.8	2e−21	1	0.2	1	0.003

k

H3K36me3 WT str. 17 + PAA vs. Mock + PAA

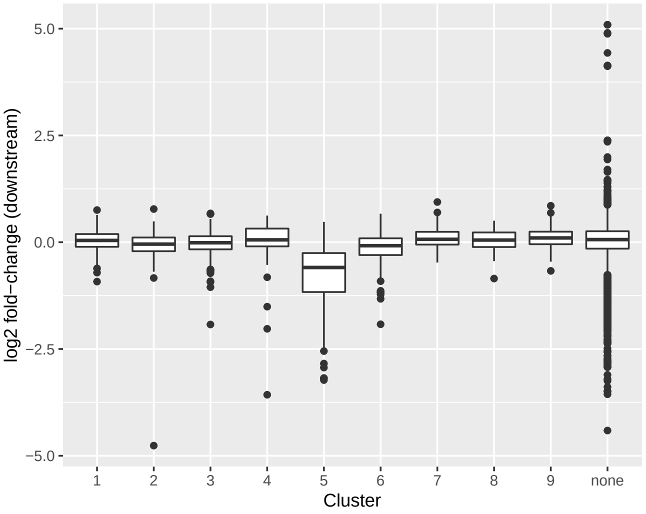

Cluster	1	2	3	4	5	6	7	8	9
p(Cl)	0.03	1	1	0.1	3e−48	2e−07	5e−06	0.3	3e−13
p(All)	1	0.005	0.002	1	3e−50	1e−14	1	1	0.01

l

H3K27me3 WT str. 17 + PAA vs. Mock + PAA

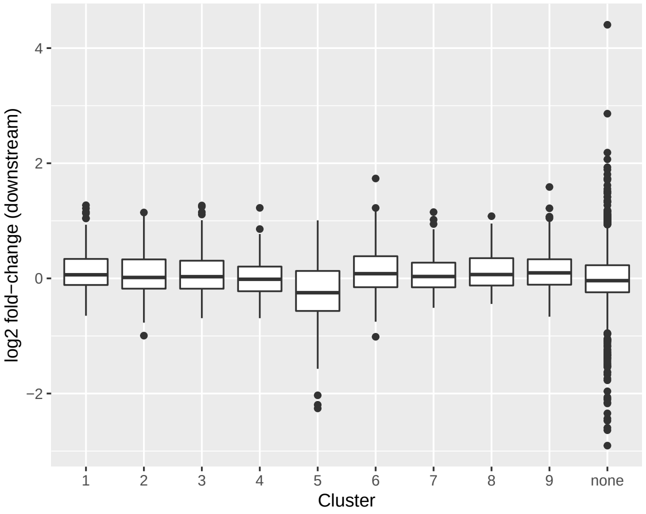

Cluster	1	2	3	4	5	6	7	8	9
p(Cl)	1	1	1	1	1e−15	1	1	1	0.4
p(All)	4e−06	1	0.01	1	1e−09	3e−05	0.2	0.04	3e−07

m

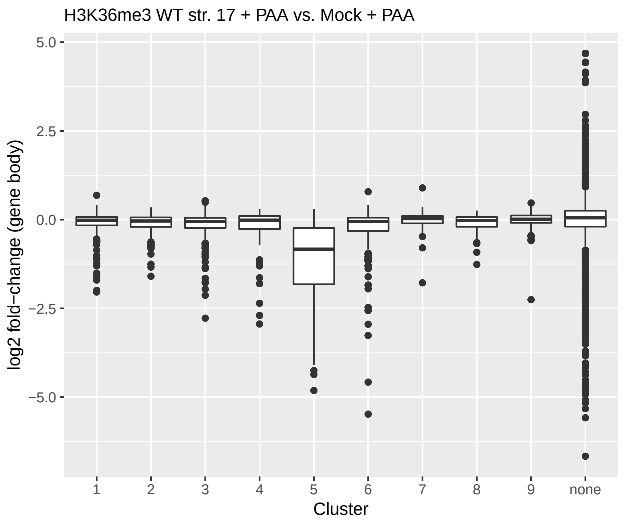

Cluster	1	2	3	4	5	6	7	8	9
p(Cl)	1	1	1	1	7e−41	1	5e−05	1	5e−10
p(All)	0.0003	0.008	6e−13	0.3	5e−47	2e−13	1	0.07	1

n

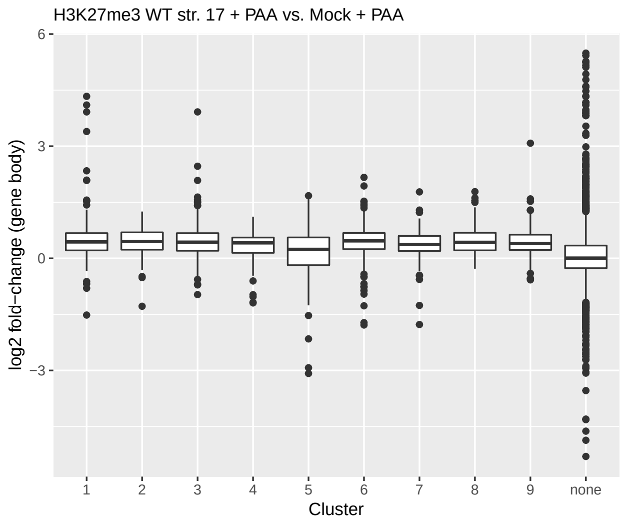

Cluster	1	2	3	4	5	6	7	8	9
p(Cl)	1	1	1	1	2e−06	1	1	1	1
p(All)	7e−36	5e−16	2e−49	3e−06	1	7e−44	1e−16	8e−17	7e−33

Sup. Fig. 11: (a) Metagene plot showing the occupancy profile of H1 for Cluster 5 genes, i.e. with strong induction of dOCRs, in mock and WT strain 17 infection with PAA treatment. **(b-f)** Metagene plots showing the occupancy profile of **(b)** H1, **(c)** H3, **(d)** H4, **(e)** H3K27me3 and **(f)** H3K36me3 for genes without induction of dOCRs, i.e. all analyzed genes in clusters without Clusters 2,5 and 6, in mock and WT strain 17 infection with PAA treatment. **(a-f)** The color track at the bottom of each subfigure indicates the significance of paired two-sided Wilcoxon tests comparing the normalized transcript coverages for each bin between mock and WT infection. P-values are adjusted for multiple testing with the Bonferroni method within each subfigure; color code: red = adj. p-value $\leq 10^{-5}$, orange = adj. p-value $\leq 10^{-3}$, yellow = adj. p-value ≤ 0.05. Exact p-values are included in the source data provided as a Source Data file. **(g-n)** Results of genome-wide differential analyses on histones and histone modifications. Log2 fold-changes for HSV-1 infection vs. mock were determined with edgeR for genomic regions around promoters (TSS $\pm$ 1.5kb), gene bodies (TSS + 1.5kb to TTS) and downstream regions (TTS to TTS + 25 kb) for all annotated protein-coding and lincRNA genes. Boxplots show the distribution of log2 fold-changes separately for genes in each cluster (n = 609, 290, 851, 176, 305, 701, 367, 289, and 574 genes for clusters 1-9, respectively) as well as the remaining genes (“none”, n= 7,915 genes) for the comparison of WT strain 17 + PAA vs. mock for downstream regions **(g,h,k,l)** and gene bodies **(i,j,m,n)** in **(g,i)** H1, **(h,j)** H3, **(k,m)** H3K36me3 and **(l,n)** H3K27me3. Bounds of boxes are the first and third quartiles for each condition. The center (median) is shown by the horizontal line in the box. Whiskers extend to 1.5 times the inter-quartile range. Outliers are shown as small circles and minimum and maximum values are lowest and highest circles, respectively. Numbers in the table at the bottom show p-values for two-sided Wilcoxon tests comparing log2 fold-changes in each cluster against log2 fold-changes for genes in all other clusters ($p(Cl)$) or against all other genes included in the differential analysis ($p(All)$). P-values were adjusted for multiple testing with the Bonferroni method separately for each histone/histone modification mark.

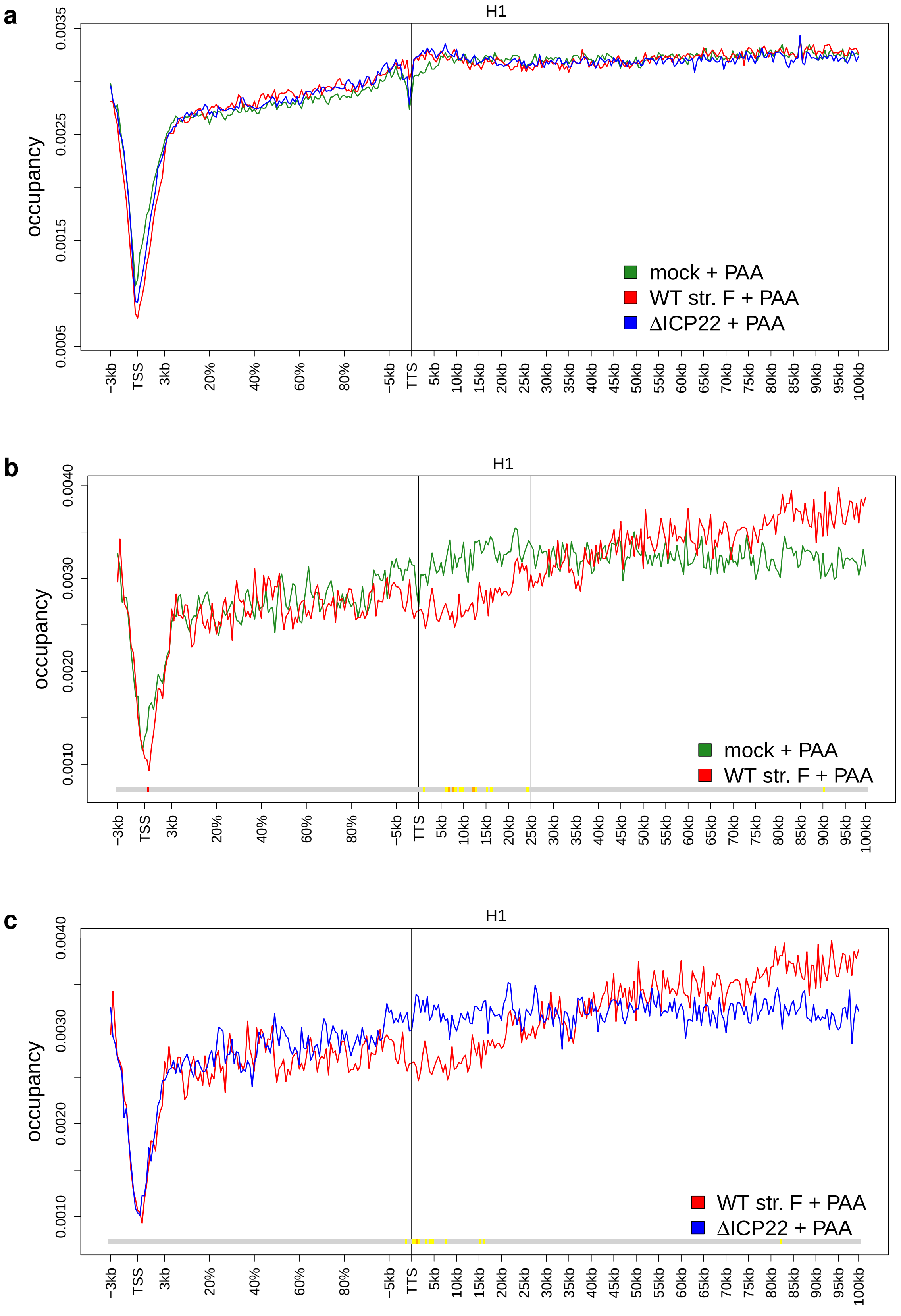
a
H1
occupancy
0.0035
0.0025
0.0015
0.0005
mock + PAA
WT str. F + PAA
ΔICP22 + PAA
−3kb
TSS
3kb
20%
40%
60%
80%
−5kb
TTS
5kb
10kb
15kb
20kb
25kb
30kb
35kb
40kb
45kb
50kb
55kb
60kb
65kb
70kb
75kb
80kb
85kb
90kb
95kb
100kb
b
H1
occupancy
0.0040
0.0030
0.0020
0.0010
mock + PAA
WT str. F + PAA
c
H1
occupancy
0.0040
0.0030
0.0020
0.0010
WT str. F + PAA
ΔICP22 + PAA

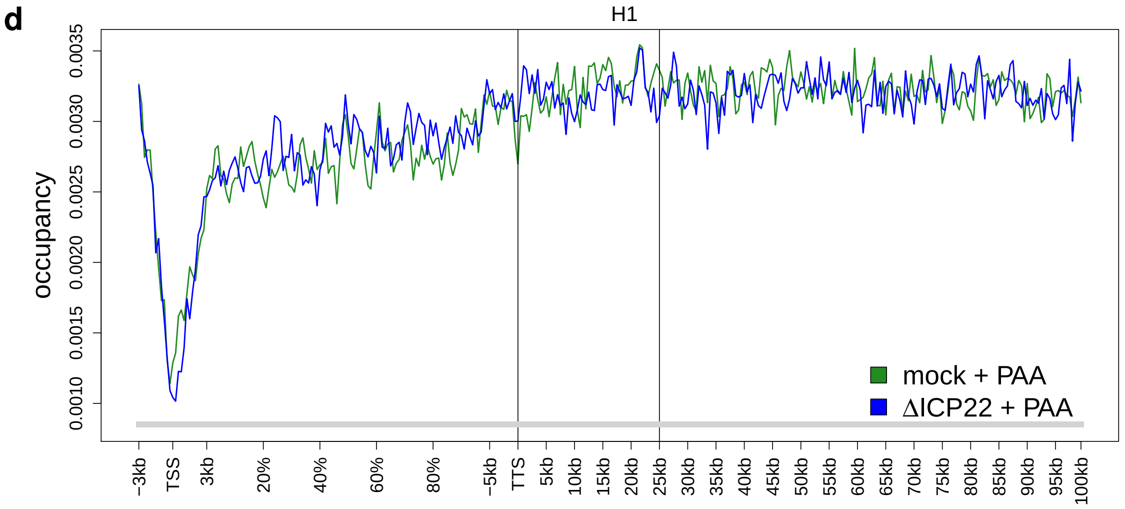

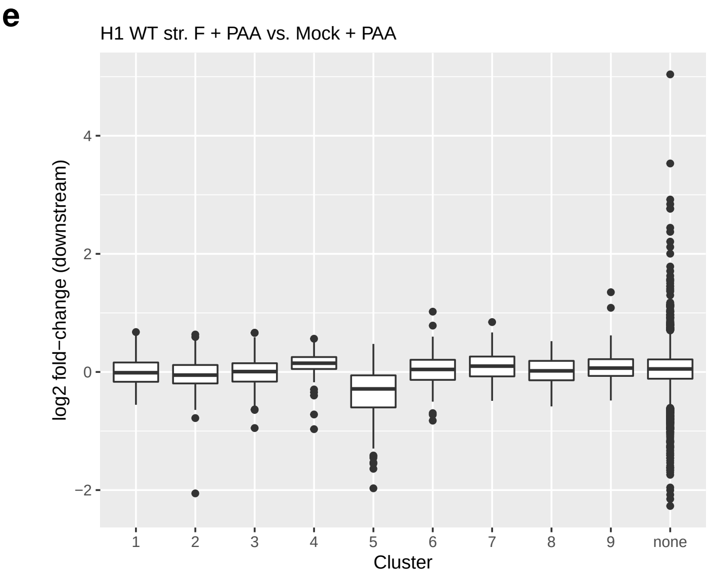

Cluster	1	2	3	4	5	6	7	8	9
p(Cl)	1	0.3	1	3e−05	4e−29	1	0.0001	1	0.0001
p(All)	0.02	0.003	0.005	0.006	7e−33	1	0.2	1	1

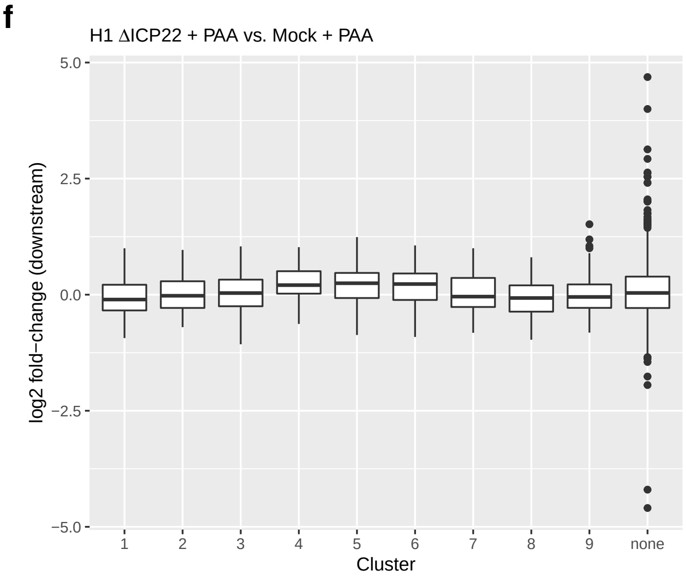

Cluster	1	2	3	4	5	6	7	8	9
p(Cl)	8e−05	1	1	0.0002	0.0001	4e−09	1	0.04	0.05
p(All)	0.003	1	1	0.0006	0.0005	3e−07	1	0.2	0.4

Sup. Fig. 12: (a) Metagene plot for histone H1 for genes without induction of dOCRs, i.e. genes from all clusters except Clusters 2,5, and 6, for mock, WT strain F and ΔICP22 + PAA infection. **(b-d)** Metagene plots for histone H1 for Cluster 5 genes for the pairwise comparison of **(b)** mock vs. WT strain F infection, **(c)** WT strain F vs. ΔICP22 infection and **(d)** mock vs. ΔICP22 infection (all with PAA treatment). The color track at the bottom of each panel indicates the significance of paired two-sided Wilcoxon tests comparing the normalized transcript coverages for each bin between mock and WT infection. P-values are adjusted for multiple testing with the Bonferroni method within each subfigure; color code: red = adj. p-value $\leq 10^{-5}$, orange = adj. p-value $\leq 10^{-3}$, yellow = adj. p-value ≤ 0.05. Exact p-values are included in the source data provided as a Source Data file. **(e,f)** Results of genome-wide differential analyses on histone H1 in mock, WT strain F, ΔICP22 + PAA infection. Log2 fold-changes for HSV-1 infection vs. mock were determined with edgeR for genomic regions around promoters (TSS $\pm$ 1.5kb), gene bodies (TSS + 1.5kb to TTS) and downstream regions (TTS to TTS + 25 kb) for all annotated protein-coding and lincRNA genes. Boxplots show the distribution of log2 fold-changes for downstream regions separately for genes in each cluster (n = 609, 290, 851, 176, 305, 701, 367, 289, and 574 genes for clusters 1-9, respectively) as well as remaining genes ("none", n= 7,915 genes) for the comparison between **(e)** WT strain F vs. mock and **(f)** ΔICP22 vs. mock. Bounds of boxes are the first and third quartiles for each condition. The center (median) is shown by the horizontal line in the box. Whiskers extend to 1.5 times the inter-quartile range. Outliers are shown as small circles and minimum and maximum values are lowest and highest

circles, respectively. Numbers in the table at the bottom of each panel show p-values for two-sided Wilcoxon tests comparing log2 fold-changes in each cluster against log2 fold-changes for genes in all other clusters ($p(Cl)$) or against all other genes included in the differential analysis ($p(All)$). P-values were adjusted for multiple testing with the Bonferroni method separately for each comparison.

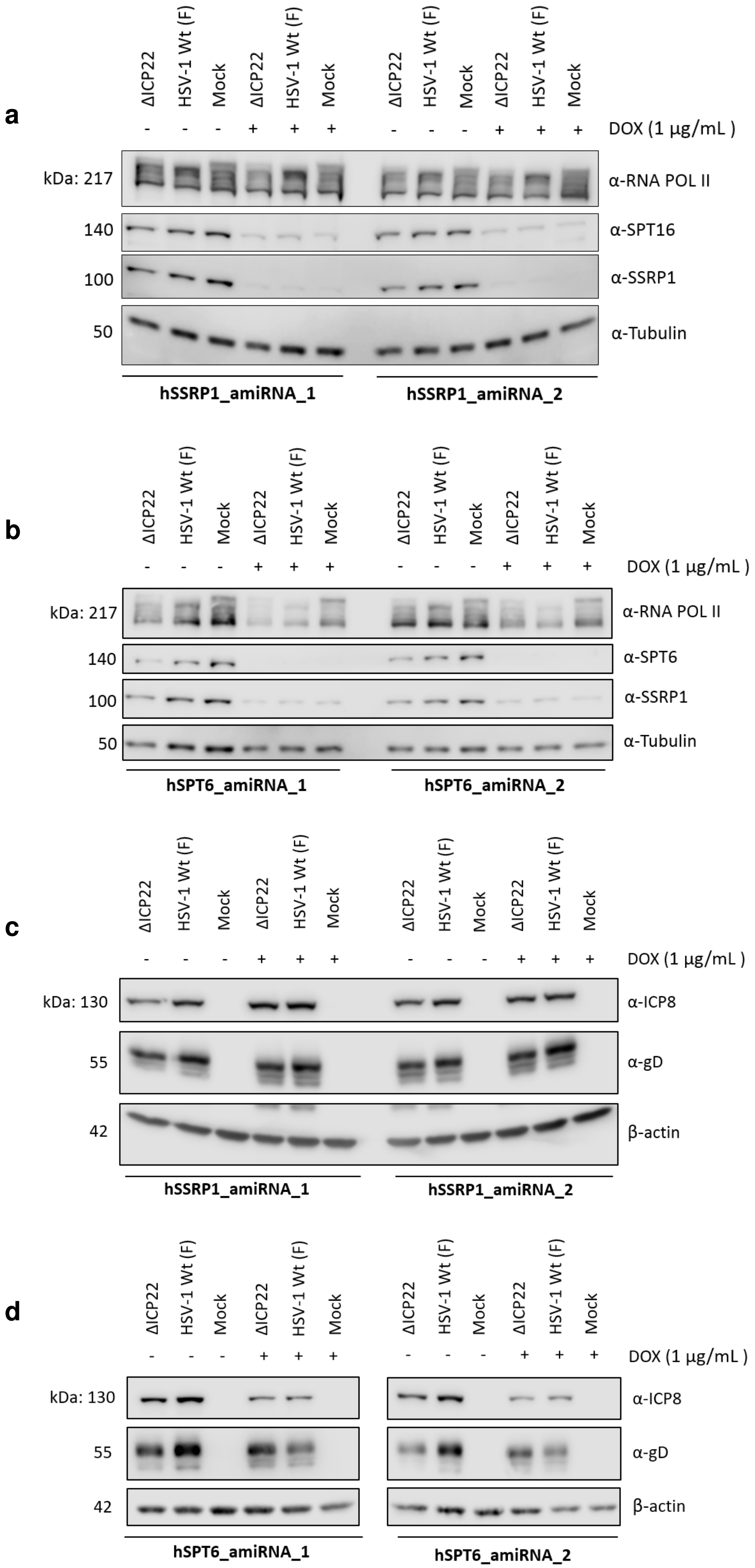
a
ΔICP22
HSV-1 Wt (F)
Mock
DOX (1 µg/mL)
kDa: 217
140
100
50
α-RNA POL II
α-SPT16
α-SSRP1
α-Tubulin
hSSRP1_amiRNA_1
hSSRP1_amiRNA_2
b
DOX (1 µg/mL)
kDa: 217
140
100
50
α-RNA POL II
α-SPT6
α-SSRP1
α-Tubulin
hSPT6_amiRNA_1
hSPT6_amiRNA_2
c
DOX (1 µg/mL)
kDa: 130
55
42
α-ICP8
α-gD
β-actin
hSSRP1_amiRNA_1
hSSRP1_amiRNA_2
d
DOX (1 µg/mL)
kDa: 130
55
42
α-ICP8
α-gD
β-actin
hSPT6_amiRNA_1
hSPT6_amiRNA_2

e

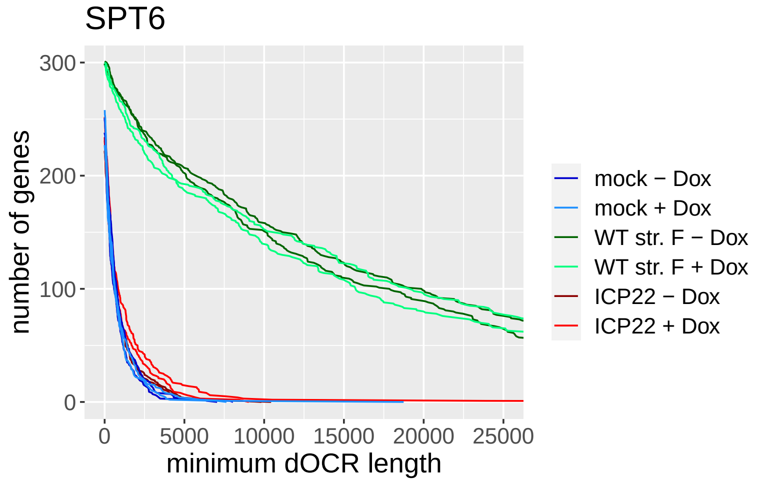

f

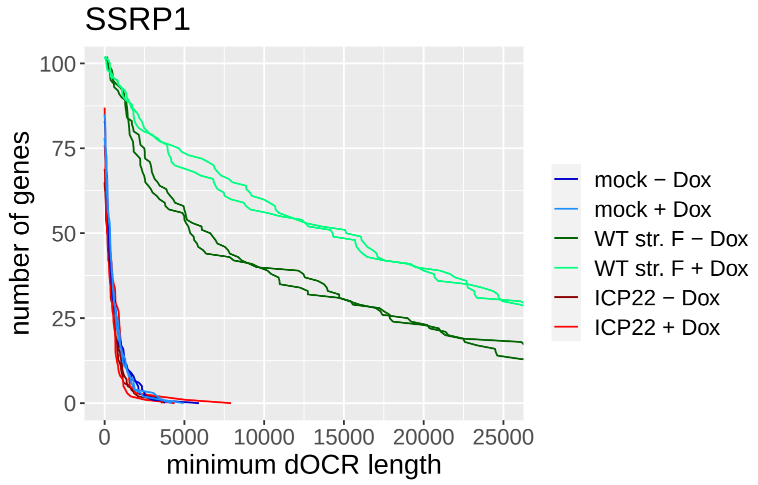

Sup. Fig. 13: (a-b) Knock-down of **(a)** SSRP1 and **(b)** SPT6 in T-HFs by an doxycycline (Dox)-inducible, lentiviral construct encoding an artificial miRNAs (amiRNAs). Each experiment was repeated independently twice with similar results. **(a)** Knock-down of SSRP1 was induced with 1 μg/mL Dox (hSSRP1_amiRNA_1 and hSSRP1_amiRNA_2) and compared to control cells. **(b)** Knock-down of SPT6 was induced with 1 μg/mL Dox (hSPT6_amiRNA_1 and hSPT6_amiRNA_2) and compared to control cells. In both cases, cells were infected with HSV-1 WT strain F or ΔICP22 at an MOI of 10 for 8 h. PAA was used at 350 μg/mL during the course of infection. Infections were started 72 h post-induction with Dox and total lysates were collected at 8 h p.i. Western blot was performed using antibodies, as indicated. α-Tubulin was used as a loading control. **(c-d)** Expression of HSV-1 proteins in **(c)** SSRP1 and **(d)** SPT6 knock-down cells. Cells were infected with HSV-1 WT strain F or ΔICP22 at an MOI of 10 or Mock infected for 8 hours. PAA was used at 350 μg/mL during the course of infection. Total cell lysates were collected at 72 hours post-induction with Dox. Western blots were probed for two HSV-1 viral proteins, ICP8 and glycoprotein D. β-actin was used as a loading control. **(e)** Number of genes in Cluster 5 for which dOCRs reach a length greater than the value indicated on the x-axis in mock, WT strain F and ΔICP22 without or with Dox-induced knockdown of SPT6 after down-sampling of reads to approximately the same number of reads mapping to the human genome. For each condition, this figure includes all Cluster 5 genes with a dOCR length > 0 for that particular condition. **(f)** Fig. 5b restricted to Cluster 5 genes with no known protein-coding or lincRNA gene within 50kb downstream of their gene 3'end (=103 genes).

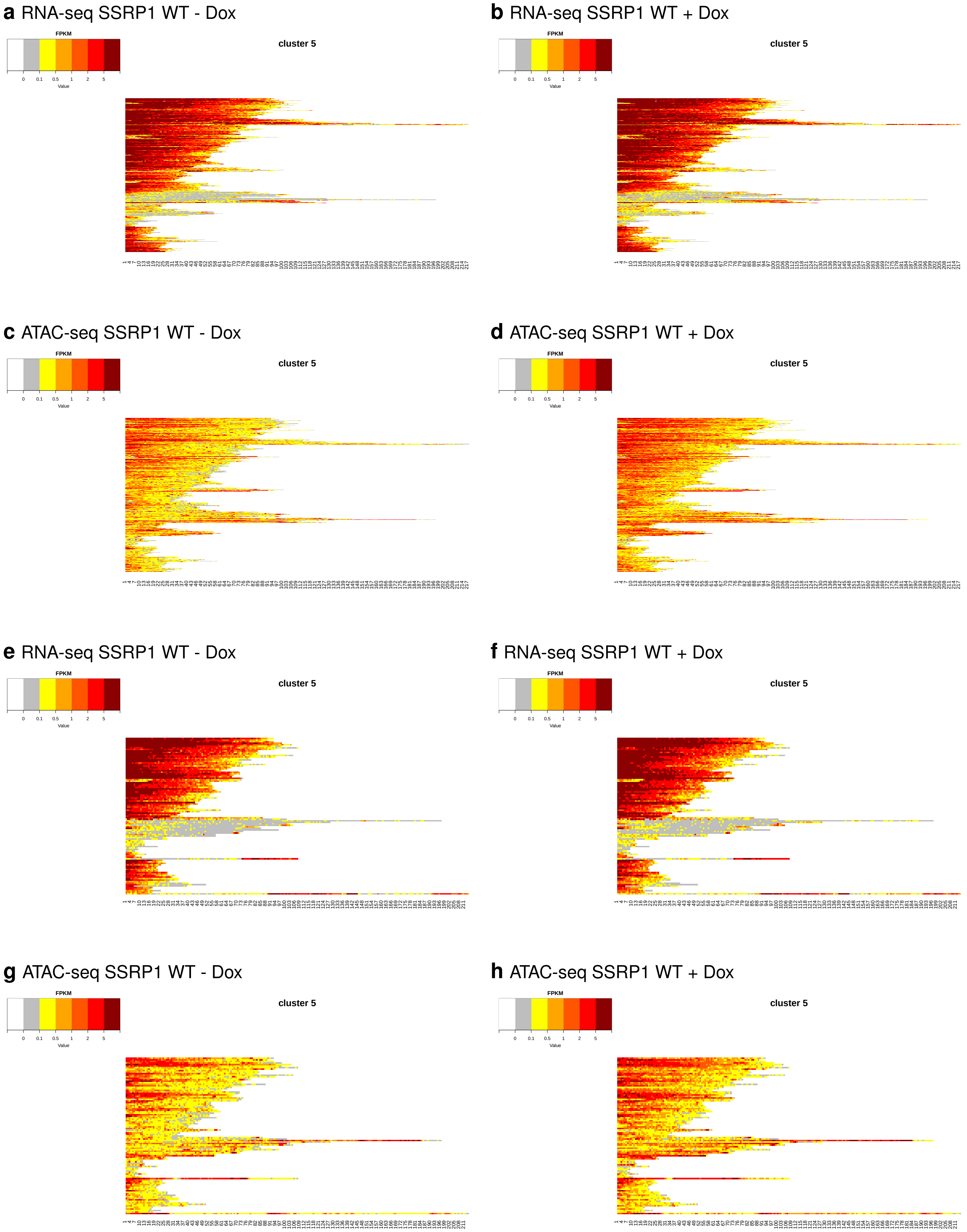

Sup. Fig. 14: Figures as in Sup. Fig. 4 showing **(a,b,e,f)** RNA-seq and **(c,d,g,h)** ATAC-seq read coverage in WT infection **(a,c,e,g)** without and **(b,d,g,h)** with Dox-induced SSRP1 knock-down on dOCR regions that were identified in WT infection with Dox-induced SSRP1 knock-down for **(a-d)** all

Cluster 5 genes and **(e-h)** Cluster 5 genes without known protein-coding and lincRNA genes within 50kb of their 3'end (=105 genes). Genes were clustered according to ATAC-seq read coverage on dOCR regions in WT infection with Dox-induced SSRP1 knock-down. For a description of this type of figure see caption to Sup. Fig. 4.

a

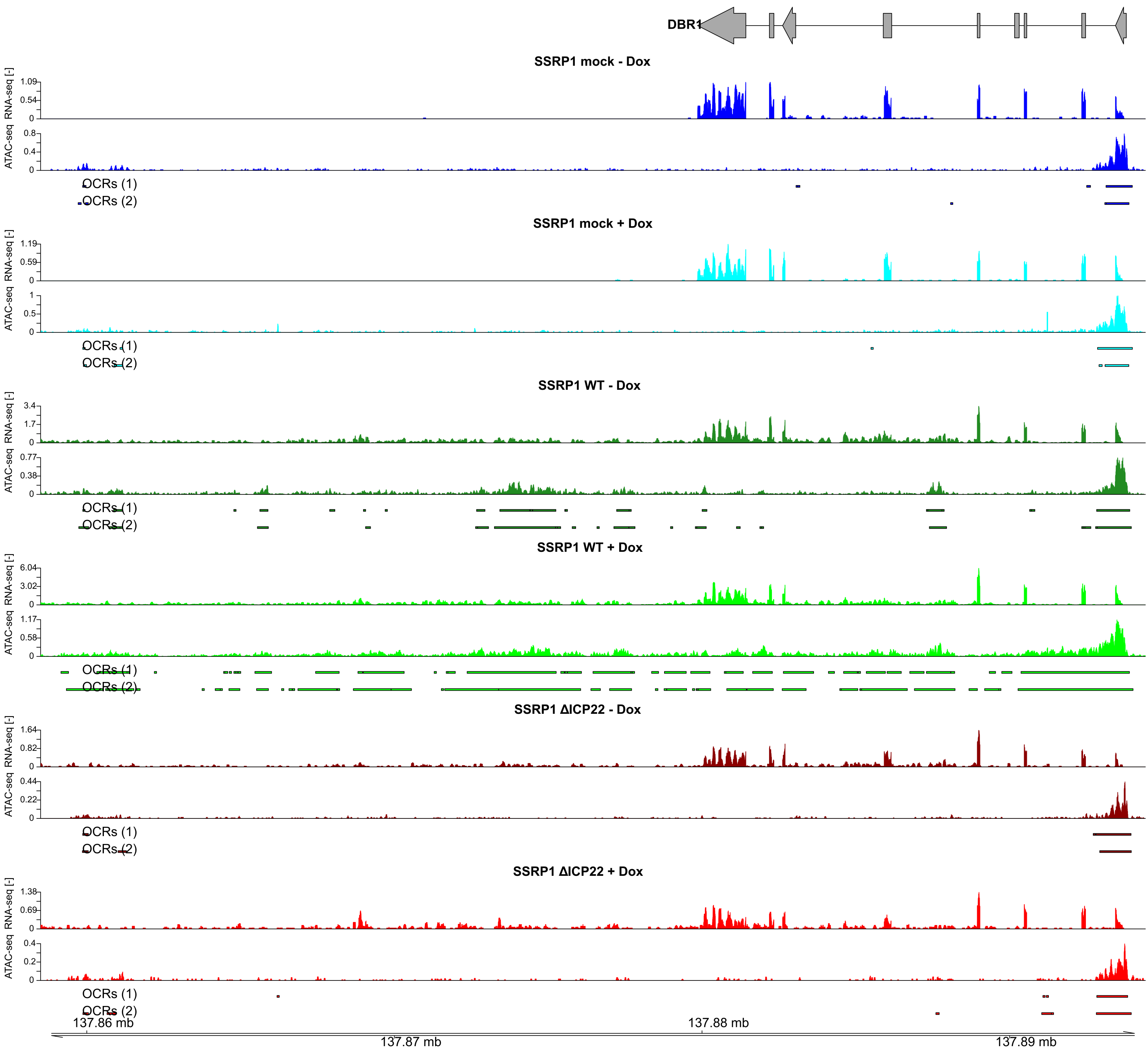
DBR1
SSRP1 mock - Dox
RNA-seq [-]
1.09
0.54
0
ATAC-seq
0.8
0.4
0
OCRs (1)
OCRs (2)
SSRP1 mock + Dox
1.19
0.59
1
0.5
SSRP1 WT - Dox
3.4
1.7
0.77
0.38
SSRP1 WT + Dox
6.04
3.02
1.17
0.58
SSRP1 ΔICP22 - Dox
1.64
0.82
0.44
0.22
SSRP1 ΔICP22 + Dox
1.38
0.69
0.4
0.2
137.86 mb
137.88 mb
137.87 mb
137.89 mb

b

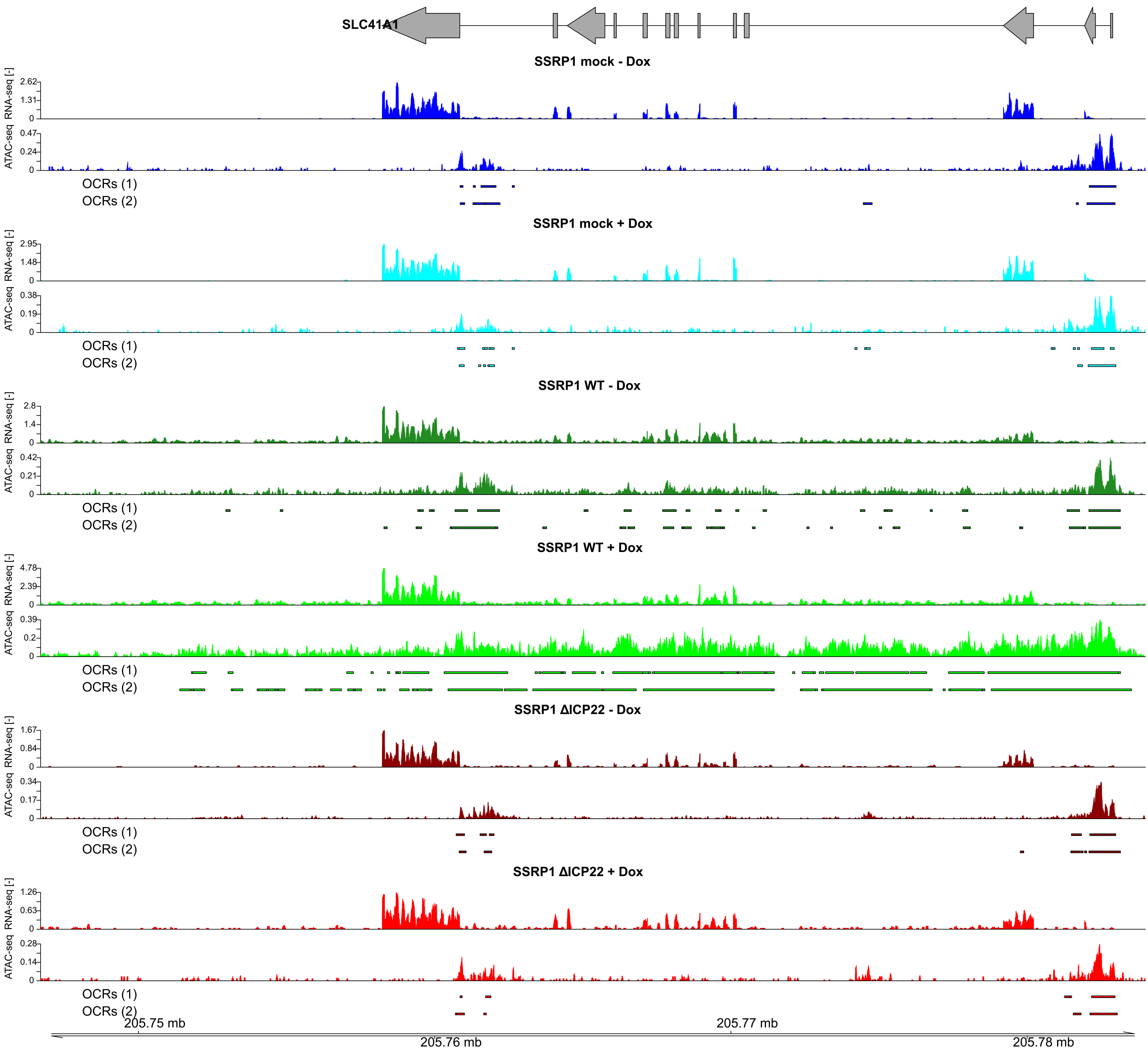
SLC41A1
SSRP1 mock - Dox
SSRP1 mock + Dox
SSRP1 WT - Dox
SSRP1 WT + Dox
SSRP1 ΔICP22 - Dox
SSRP1 ΔICP22 + Dox
RNA-seq [-]
ATAC-seq
OCRs (1)
OCRs (2)
2.62
1.31
0.47
0.24
2.95
1.48
0.38
0.19
2.8
1.4
0.42
0.21
4.78
2.39
0.39
0.2
1.67
0.84
0.34
0.17
1.26
0.63
0.28
0.14
0
205.75 mb
205.77 mb
205.76 mb
205.78 mb

c

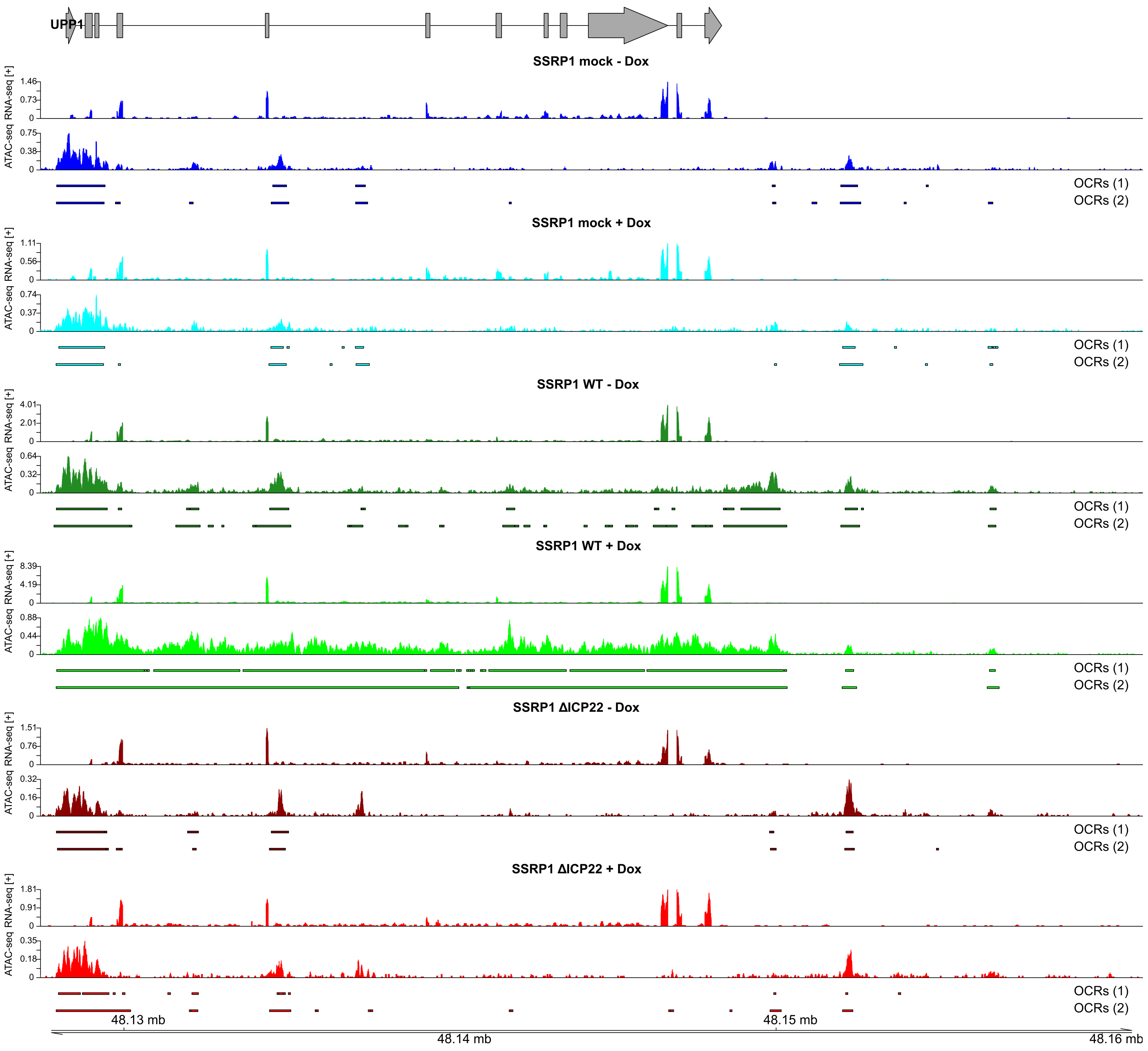

Sup. Fig. 15: Example genes (**(a)** DBR1, **(b)** SLC41A1, **(c)** UPP1) showing increased chromatin accessibility within the gene body in SSRP1-depleted cells in HSV-1 infection. DBR1 and SLC41A1, but not UPP1, exhibit read-through in HSV-1 infection. Tracks show total RNA-seq (strand-specific) and ATAC-seq (non-strand-specific) read coverage (normalized to total number of mapped human reads; averaged between replicates) in mock, WT and ΔICP22 infection without and with Dox-induced SSRP1 depletion. Identified OCRs for both replicates are shown separately below the ATAC-seq read coverage tracks. Gene annotation is indicated at the top. Boxes represent exons and lines introns and direction is indicated by arrowheads. Genomic coordinates are shown on the bottom.
