# Supplementary material for: The HSV-1 ICP22 protein selectively impairs histone repositioning upon Pol II transcription downstream of genes
Source: Nat Commun. 2023 Jul 31;14:4591. doi: 10.1038/s41467-023-40217-w (PMC10390501; doi:10.1038/s41467-023-40217-w)
Supplement: Supplementary file 2 — Description of Additional Supplementary Files [file 41467_2023_40217_MOESM2_ESM.pdf]

Supplementary Data 1

Statistics on all (Omni-)ATAC-seq samples obtained for this study. Peaks were identified using F-Seq. Peaks were annotated using the R/Bioconductor package ChIPseeker to determine the fraction of peaks in promoters (TSS +/- 3kb), gene bodies (5' UTR/3' UTR/Exon/Intron), immediate downstream of a gene (within 3kb) or in distal intergenic regions. Peaks either annotated as downstream according to ChIPseeker or included in dOCRs were classified as “downstream / dOCR”. PT Score was calculated the R/Bioconductor package ATACseqQC. FRiP (fraction of reads in peaks) was calculated using featureCounts with peaks as annotation.

Supplementary Data 2

Statistics on all ChIPmentation samples obtained for this study. Peaks were identified using F-Seq. Peaks were annotated using the R/Bioconductor package ChIPseeker to determine the fraction of peaks in promoters (TSS +/- 3kb), gene bodies (5' UTR/3' UTR/Exon/Intron), immediate downstream of a gene (within 3kb) or in distal intergenic regions. PT Score was calculated the R/Bioconductor package ATACseqQC. FRiP (fraction of reads in peaks) was calculated using featureCounts with peaks as annotation.

Supplementary Data 3

List of PCR primers and sequencing adaptors (i5, i7).
