# Supplementary material for: The HSV-1 ICP22 protein selectively impairs histone repositioning upon Pol II transcription downstream of genes
Source: Nat Commun. 2023 Jul 31;14:4591. doi: 10.1038/s41467-023-40217-w (PMC10390501; doi:10.1038/s41467-023-40217-w)
Supplement: Supplementary file 6 — Reporting Summary [file 41467_2023_40217_MOESM6_ESM.pdf]

nature portfolio

Corresponding author(s): Caroline C. Friedel and Lars Dölken

Last updated by author(s): Jul 8, 2023

Reporting Summary

Nature Portfolio wishes to improve the reproducibility of the work that we publish. This form provides structure for consistency and transparency in reporting. For further information on Nature Portfolio policies, see our Editorial Policies and the Editorial Policy Checklist.

Statistics

For all statistical analyses, confirm that the following items are present in the figure legend, table legend, main text, or Methods section.

n/a	Confirmed	
☐	☒	The exact sample size (*n*) for each experimental group/condition, given as a discrete number and unit of measurement
☐	☒	A statement on whether measurements were taken from distinct samples or whether the same sample was measured repeatedly
☐	☒	The statistical test(s) used AND whether they are one- or two-sided<br>*Only common tests should be described solely by name; describe more complex techniques in the Methods section.*
☒	☐	A description of all covariates tested
☐	☒	A description of any assumptions or corrections, such as tests of normality and adjustment for multiple comparisons
☐	☒	A full description of the statistical parameters including central tendency (e.g. means) or other basic estimates (e.g. regression coefficient) AND variation (e.g. standard deviation) or associated estimates of uncertainty (e.g. confidence intervals)
☐	☒	For null hypothesis testing, the test statistic (e.g. *F*, *t*, *r*) with confidence intervals, effect sizes, degrees of freedom and *P* value noted<br>*Give P values as exact values whenever suitable.*
☒	☐	For Bayesian analysis, information on the choice of priors and Markov chain Monte Carlo settings
☒	☐	For hierarchical and complex designs, identification of the appropriate level for tests and full reporting of outcomes
☐	☒	Estimates of effect sizes (e.g. Cohen's *d*, Pearson's *r*), indicating how they were calculated

Our web collection on statistics for biologists contains articles on many of the points above.

Software and code

Policy information about availability of computer code

Data collection	Agilent Bioanalyzer 2100 with Expert Software Version B.02.11.SI824 (SR1), Firmware C.01.069, SN: DEDAE03245; DNBSEQ G400 sequencer built-in software
Data analysis	fastQC (v0.11.9), ContextMap (v2.7.9), BWA (0.7.10), samtools (v1.3), featureCounts (v1.4.6), R (v4.0.3), F-seq, BEDTools (v2.29.1), ATACseqQC (v1.14.4), ChIPseeker (v1.26.2)<br>Custom code and workflows for dOCR calculation and calculation of downstream transcription activity available at: https://github.com/watchdog-wms/watchdog-wms-modules and https://github.com/watchdog-wms/watchdog-wms-workflows<br>R scripts for figure creation available at https://doi.org/10.5281/zenodo.7853167

For manuscripts utilizing custom algorithms or software that are central to the research but not yet described in published literature, software must be made available to editors and reviewers. We strongly encourage code deposition in a community repository (e.g. GitHub). See the Nature Portfolio guidelines for submitting code & software for further information.

Data

Policy information about availability of data

All manuscripts must include a data availability statement. This statement should provide the following information, where applicable:

- Accession codes, unique identifiers, or web links for publicly available datasets
- A description of any restrictions on data availability
- For clinical datasets or third party data, please ensure that the statement adheres to our policy

All sequencing data have been deposited at Gene Expression Omnibus (GEO) under accession code GSE185241 and GSE185239. Source data are provided with this paper.

Research involving human participants, their data, or biological material

Policy information about studies with human participants or human data. See also policy information about sex, gender (identity/presentation), and sexual orientation and race, ethnicity and racism.

Reporting on sex and gender	Not applicable.
Reporting on race, ethnicity, or other socially relevant groupings	Not applicable.
Population characteristics	Not applicable.
Recruitment	Not applicable.
Ethics oversight	Not applicable.

Note that full information on the approval of the study protocol must also be provided in the manuscript.

Field-specific reporting

Please select the one below that is the best fit for your research. If you are not sure, read the appropriate sections before making your selection.

☒ Life sciences ☐ Behavioural & social sciences ☐ Ecological, evolutionary & environmental sciences

For a reference copy of the document with all sections, see nature.com/documents/nr-reporting-summary-flat.pdf

Life sciences study design

All studies must disclose on these points even when the disclosure is negative.

Sample size	We performed ChIP-seq, ATAC-seq and OMNI-ATAC-seq generally using two biological replicates unless otherwise stated in the figure legends. A sample size of two was chosen to ensure reproducibility while keeping the costs at bay. Two replicates are usually sufficient for these kind of analysis as many different genomic loci can be analyzed as well as meta-analysis performed across multiple genomic loci.
Data exclusions	No data were excluded from analyses
Replication	All experimental biological replicates for ATAC-seq and OMNI-ATAC-seq were performed with the same virus stocks and with cells that were split and seeded separately. We reproduced ATAC-seq findings with multiple strains and ICP22 mutants, and without the context of viral replication. No samples failed or were excluded. ChIP-seq biological replicates were performed on separate days with the same batch of cells but the same virus stocks. No samples failed or were excluded. Findings for histone positioning were reproduced as two biological replicates for H1 histone using isogenic WT and delta-ICP22 strains. Knock-down experiments were performed using two different miRNAs (expressed in transduced cells). All knockdown experiments showed the expected knockdowns of the genes of interest. No samples failed or were excluded from the analysis.
Randomization	Not applicable as computational analysis was performed using the same established analysis pipelines without subsequent adjustments for the obtained outputs.
Blinding	Not applicable as computational analysis was performed using the same established analysis pipelines without subsequent adjustments for the obtained outputs.

Reporting for specific materials, systems and methods

We require information from authors about some types of materials, experimental systems and methods used in many studies. Here, indicate whether each material, system or method listed is relevant to your study. If you are not sure if a list item applies to your research, read the appropriate section before selecting a response.

Materials & experimental systems

n/a	Involved in the study	
☐	☒	Antibodies
☐	☒	Eukaryotic cell lines
☒	☐	Palaeontology and archaeology
☒	☐	Animals and other organisms
☒	☐	Clinical data
☒	☐	Dual use research of concern
☒	☐	Plants

Methods

n/a	Involved in the study	
☐	☒	ChIP-seq
☒	☐	Flow cytometry
☒	☐	MRI-based neuroimaging

Antibodies

Antibodies used

For common tags:
anti-V5 (Cell Signaling #13202, lot 7, 1:500), anti-HA clone 11 (Biolegend #16B12, lot B269834, 1:1000), anti-FLAG (Sigma-Aldrich # F3165, 1:1000)

For human house keeping genes:
anti-β-Actin clone C-4 (Santa Cruz Biotechnology #sc-47778, 1:1000), anti-GAPDH (Cell Signaling #2118, lot 8, 1:1000), anti-α-Tubulin (Cell Signaling #2144, 1:1000)

For human genes of interest:
anti-SPT6 (Novus Biologicals #NB100-2582, 1:500); anti-SSRP1 (Biolegend #609710, 1:350), anti-Spt16 clone 8D2 (BioLegend #607008, 1:500), anti-RNA Pol II 1F4B6 (Active Motif #2687513, lot 17316002, 1:1000)

For HSV-1 proteins:
anti-ICP4 10F1 (Santa Cruz, sc-56986, lot H0217, 1:1000), anti-ICP8 clone 11E2 (Santa Cruz Biotechnology #sc-53330, lot A0914, 1:1000), anti-gD clone DL6 (Santa Cruz Biotechnology #sc-21719, lot K2515, 1:1000)

For histone proteins and histone marks:
anti-H1 antibody (Invitrogen #PA5-30055, lot VF3008931B, 1:100/ChIP), anti-H3 antibody (Invitrogen, PA5-16183, lot 1923404, 1:50/ChIP), anti-H4 antibody (Cell Signaling, #14149S, lot 1, 1:50/ChIP), anti-H3K27me3 (Diagenode, #C15410195, lot A1811-001P, 1 µg/ChIP), anti-H3K36me3 (Diagenode, #C15410192, 1 µg/ChIP)

Isotype control antibodies: normal rabbit IgG (Cell Signaling, #2729S, lot 8), mouse IgG1 isotype (Cell Signaling, #5415S , lot 10), mouse IgG2a isotype (Biolegend, #401501, lot B243565), mouse IgG2b isotype (Cell Signaling, #53484S, lot 1). These isotype control antibodies were used at the highest amount of the actual antibody (e.g., if 1 µg/ChIP of a IgG1 antibody was used this will also used for the isotype control).

Secondary antibodies:
anti-rabbit–horseradish peroxidase (HRP, Sigma-Aldrich #A0545) at a 1:10,000 dilution, anti-mouse–HRP (Sigma-Aldrich #A9044) at a 1:10,000 dilution, anti-rat-HRP (Sigma-Aldrich #SAB3700539) at a 1:5,000 diluation, or IRDye 680RD goat-anti-rabbit IgG (Licor #926-68071) and IRDye 800CW donkey anti-mouse IgG secondary antibody (Licor #926-32212) at 1:5,000 dilution each.

Validation

Antibodies to common tags were validated by immunoblot and/or immunofluorescence analysis after transfection of a respective expression plasmid into HEK293 cells.

Antibodies to house-keeping genes were validated by immunoblot based on the size of the obtained major band.

The anti-Spt6, anti-SSRP1 and anti-Pol-II antibodies were validated with immunoblotting including the respective shRNA knock-downs for two different shRNAs.

The anti-ICP4, anti-ICP8 and anti-gD antibodies are well established in our lab and were initially validated by us comparing cells infected with mock, wild-type and KO viruses by immunoblot and immunofluorescence analysis.

All primary antibodies for histones or histone modifications are well established in the field for ChIP-seq. They were validated by immunoblotting and subsequently by comparing the obtained ChIP-seq data to textbook knowledge on the expected ChIP-seq profiles from uninfected human cells (for peaks or lack of reads in certain areas). No discrepancies were noted.

Eukaryotic cell lines

Policy information about cell lines and Sex and Gender in Research

Cell line source(s)

Human Fetal Foreskin Fibroblasts (obtained directly from ECACC), Human Fetal Foreskin Fibroblasts immortalized with telomerase (obtained from Prof. Micheal Weekes; PMID: 30122656); Baby Hamster Kidney Cells (BHK) for production of HSV-1 stocks were obtained from Dr. Colin Crump, Cambridge.

Authentication	HFFF were purchased from ECACC by Lars Dölken; HFF-tert cells were obtained from Prof. Michael Weekes and authenticated in PMID:30122656. Cells were continuously monitored for morphology, their slow growth kinetics and their susceptibility to HSV-1.<br>BHK cells were continuously monitored based on their ability to produce large amounts of HSV-1 for virus stocks.<br>All cells were used at low passage for all assays described. Stable cells were transduced at low passage and used as polyclonal cell lines at low passage. Large batches of cells were frozen down in early passages either upon receipt or once transduced. Cells were thawed at least 3 passages prior to an experiment and not maintained longer than 20 passages.
Mycoplasma contamination	Tissue culture plates were only touched with gloves. Cell lines were tested upon receipt and tested periodically. All virus stocks were tested for mycoplasma prior to use. All results were negative.
Commonly misidentified lines<br>(See ICLAC register)	No commonly misidentified cell lines were used in this study.

Plants

Seed stocks	Not applicable.
Novel plant genotypes	Not applicable.
Authentication	Not applicable.

ChIP-seq

Data deposition

☒ Confirm that both raw and final processed data have been deposited in a public database such as GEO.

☒ Confirm that you have deposited or provided access to graph files (e.g. BED files) for the called peaks.

Data access links<br>*May remain private before publication.*	https://www.ncbi.nlm.nih.gov/geo/query/acc.cgi?acc=GSE185241 (secure token while it remains in private status: cfileamkphijvqj)
Files in database submission	fastq files:<br>H3K27me3_mock_1:MOCK_H3K27me3_R1.fq.gz,MOCK_H3K27me3_R2.fq.gz<br>H3K27me3_mock_2:M24_H3K27me3_R1.fq.gz,M24_H3K27me3_R2.fq.gz<br>H3K27me3_wt_1:HSV_1_H3K27me3_R1.fq.gz,HSV_1_H3K27me3_R2.fq.gz<br>H3K27me3_wt_2:HSV29_H3K27me3_R1.fq.gz,HSV29_H3K27me3_R2.fq.gz<br>H3K36me3_mock_1:MOCK_H3K36me3_R1.fq.gz,MOCK_H3K36me3_R2.fq.gz<br>H3K36me3_mock_2:M23_H3K36me3_R1.fq.gz,M23_H3K36me3_R2.fq.gz<br>H3K36me3_mock_3:M43_H3K36me3_R1.fq.gz,M43_H3K36me3_R2.fq.gz<br>H3K36me3_wt_1:HSV_1_H3K36me3_R1.fq.gz,HSV_1_H3K36me3_R2.fq.gz<br>H3K36me3_wt_2:HSV28_H3K36me3_R1.fq.gz,HSV28_H3K36me3_R2.fq.gz<br>H3K36me3_wt_3:HSV48_H3K36me3_R1.fq.gz,HSV48_H3K36me3_R2.fq.gz<br>H3_total_WT17_1:HSV_1_H3_total_R1.fq.gz,HSV_1_H3_total_R2.fq.gz<br>H3_total_WT17_2:HSV27_Total_H3_R1.fq.gz,HSV27_Total_H3_R2.fq.gz<br>H3_total_mock_1:MOCK_H3_total_R1.fq.gz,MOCK_H3_total_R2.fq.gz<br>H3_total_mock_2:M22_Total_H3_R1.fq.gz,M22_Total_H3_R2.fq.gz<br>HSV-1_Wt_H1_1:200823_I66_V300060876_L4_HSV-1_Wt_H1_new_Invitrogen_1.fq.gz,200823_I66_V300060876_L4_HSV-1_Wt_H1_new_Invitrogen_2.fq.gz,200913_I66_V300063186_L2_HSV-1_Wt_H1_new_Invitrogen_1.fq.gz,200913_I66_V300063186_L2_HSV-1_Wt_H1_new_Invitrogen_2.fq.gz<br>HSV-1_Wt_str_F_H1_2:428_S1_L002_R1_001.fastq.gz,428_S1_L002_R2_001.fastq.gz<br>HSV1_17_WT_8h_PAA_H1_1:210730_M075_V350022578_L02_580_1.fq.gz,210730_M075_V350022578_L02_580_2.fq.gz,210730_M075_V350022578_L03_580_1.fq.gz,210730_M075_V350022578_L03_580_2.fq.gz<br>HSV1_17_WT_8h_PAA_H1_2:210730_M075_V350022578_L02_592_1.fq.gz,210730_M075_V350022578_L02_592_2.fq.gz,210730_M075_V350022578_L03_592_1.fq.gz,210730_M075_V350022578_L03_592_2.fq.gz<br>HSV1_17_WT_8h_PAA_H3_1:210730_M075_V350022578_L02_576_1.fq.gz,210730_M075_V350022578_L02_576_2.fq.gz,210730_M075_V350022578_L03_576_1.fq.gz,210730_M075_V350022578_L03_576_2.fq.gz<br>HSV1_17_WT_8h_PAA_H3_2:210730_M075_V350022578_L02_588_1.fq.gz,210730_M075_V350022578_L02_588_2.fq.gz,210730_M075_V350022578_L03_588_1.fq.gz,210730_M075_V350022578_L03_588_2.fq.gz<br>HSV1_17_WT_8h_PAA_H4_1:210730_M075_V350022578_L02_577_1.fq.gz,210730_M075_V350022578_L02_577_2.fq.gz,210730_M075_V350022578_L03_577_1.fq.gz,210730_M075_V350022578_L03_577_2.fq.gz<br>HSV1_17_WT_8h_PAA_H4_2:210730_M075_V350022578_L02_589_1.fq.gz,210730_M075_V350022578_L02_589_2.fq.gz,210730_M075_V350022578_L03_589_1.fq.gz,210730_M075_V350022578_L03_589_2.fq.gz<br>HSV1_17_WT_8h_PAA_IGG_1:210730_M075_V350022578_L02_594_1.fq.gz,210730_M075_V350022578_L02_594_2.fq.gz,210730_M075_V350022578_L03_594_1.fq.gz,210730_M075_V350022578_L03_594_2.fq.gz<br>HSV1_17_WT_8h_PAA_K27_1:210730_M075_V350022578_L02_579_1.fq.gz,210730_M075_V350022578_L02_579_2.fq.gz,210730_M075_V350022578_L03_579_1.fq.gz,210730_M075_V350022578_L03_579_2.fq.gz<br>HSV1_17_WT_8h_PAA_K27_2:210730_M075_V350022578_L02_591_1.fq.gz,210730_M075_V350022578_L02_591_2.fq.gz,210730_M075_V350022578_L03_591_1.fq.gz,210730_M075_V350022578_L03_591_2.fq.gz

HSV1_17_WT_8h_PAA_K36_1:210730_M075_V350022578_L02_578_1.fq.gz,210730_M075_V350022578_L02_578_2.fq.gz,210730_M075_V350022578_L03_578_1.fq.gz,210730_M075_V350022578_L03_578_2.fq.gz
HSV1_17_WT_8h_PAA_K36_2:210730_M075_V350022578_L02_590_1.fq.gz,210730_M075_V350022578_L02_590_2.fq.gz,210730_M075_V350022578_L03_590_1.fq.gz,210730_M075_V350022578_L03_590_2.fq.gz
ICP22_H1_1:200823_I66_V300060876_L4_ICP22_H1_new_Invitrogen_1.fq.gz,200823_I66_V300060876_L4_ICP22_H1_new_Invitrogen_2.fq.gz,200913_I66_V300063186_L2_ICP22_H1_new_Invitrogen_1.fq.gz,200913_I66_V300063186_L2_ICP22_H1_new_Invitrogen_2.fq.gz
ICP22_H1_2:432_S1_L002_R1_001.fastq.gz,432_S1_L002_R2_001.fastq.gz
MOCK_H1_1:200823_I66_V300060876_L4_MOCK_H1_new_Invitrogen_1.fq.gz,200823_I66_V300060876_L4_MOCK_H1_new_Invitrogen_2.fq.gz,200913_I66_V300063186_L2_MOCK_H1_new_Invitrogen_1.fq.gz,200913_I66_V300063186_L2_MOCK_H1_new_Invitrogen_2.fq.gz
MOCK_H1_2:424_S1_L002_R1_001.fastq.gz,424_S1_L002_R2_001.fastq.gz
Mock_8h_PAA_H1_1:210730_M075_V350022578_L02_573_1.fq.gz,210730_M075_V350022578_L02_573_2.fq.gz,210730_M075_V350022578_L03_573_1.fq.gz,210730_M075_V350022578_L03_573_2.fq.gz
Mock_8h_PAA_H1_2:210730_M075_V350022578_L02_586_1.fq.gz,210730_M075_V350022578_L02_586_2.fq.gz,210730_M075_V350022578_L03_586_1.fq.gz,210730_M075_V350022578_L03_586_2.fq.gz
Mock_8h_PAA_H3_1:210730_M075_V350022578_L02_569_1.fq.gz,210730_M075_V350022578_L02_569_2.fq.gz,210730_M075_V350022578_L03_569_1.fq.gz,210730_M075_V350022578_L03_569_2.fq.gz
Mock_8h_PAA_H3_2:210730_M075_V350022578_L02_582_1.fq.gz,210730_M075_V350022578_L02_582_2.fq.gz,210730_M075_V350022578_L03_582_1.fq.gz,210730_M075_V350022578_L03_582_2.fq.gz
Mock_8h_PAA_H4_1:210730_M075_V350022578_L02_570_1.fq.gz,210730_M075_V350022578_L02_570_2.fq.gz,210730_M075_V350022578_L03_570_1.fq.gz,210730_M075_V350022578_L03_570_2.fq.gz
Mock_8h_PAA_H4_2:210730_M075_V350022578_L02_583_1.fq.gz,210730_M075_V350022578_L02_583_2.fq.gz,210730_M075_V350022578_L03_583_1.fq.gz,210730_M075_V350022578_L03_583_2.fq.gz
Mock_8h_PAA_IGG_R_1:210730_M075_V350022578_L02_575_1.fq.gz,210730_M075_V350022578_L02_575_2.fq.gz,210730_M075_V350022578_L03_575_1.fq.gz,210730_M075_V350022578_L03_575_2.fq.gz
Mock_8h_PAA_K27_1:210730_M075_V350022578_L02_572_1.fq.gz,210730_M075_V350022578_L02_572_2.fq.gz,210730_M075_V350022578_L03_572_1.fq.gz,210730_M075_V350022578_L03_572_2.fq.gz
Mock_8h_PAA_K27_2:210730_M075_V350022578_L02_585_1.fq.gz,210730_M075_V350022578_L02_585_2.fq.gz,210730_M075_V350022578_L03_585_1.fq.gz,210730_M075_V350022578_L03_585_2.fq.gz
Mock_8h_PAA_K36_1:210730_M075_V350022578_L02_571_1.fq.gz,210730_M075_V350022578_L02_571_2.fq.gz,210730_M075_V350022578_L03_571_1.fq.gz,210730_M075_V350022578_L03_571_2.fq.gz
Mock_8h_PAA_K36_2:210730_M075_V350022578_L02_584_1.fq.gz,210730_M075_V350022578_L02_584_2.fq.gz,210730_M075_V350022578_L03_584_1.fq.gz,210730_M075_V350022578_L03_584_2.fq.gz

bedgraph files:
Mock_8h_PAA_H3_1.bedgraph
Mock_8h_PAA_H4_1.bedgraph
Mock_8h_PAA_K36_1.bedgraph
Mock_8h_PAA_K27_1.bedgraph
Mock_8h_PAA_H1_1.bedgraph
HSV1_17_WT_8h_PAA_H3_1.bedgraph
HSV1_17_WT_8h_PAA_H4_1.bedgraph
HSV1_17_WT_8h_PAA_K36_1.bedgraph
HSV1_17_WT_8h_PAA_K27_1.bedgraph
HSV1_17_WT_8h_PAA_H1_1.bedgraph
Mock_8h_PAA_H3_2.bedgraph
Mock_8h_PAA_H4_2.bedgraph
Mock_8h_PAA_K36_2.bedgraph
Mock_8h_PAA_K27_2.bedgraph
Mock_8h_PAA_H1_2.bedgraph
HSV1_17_WT_8h_PAA_H3_2.bedgraph
HSV1_17_WT_8h_PAA_H4_2.bedgraph
HSV1_17_WT_8h_PAA_K36_2.bedgraph
HSV1_17_WT_8h_PAA_K27_2.bedgraph
HSV1_17_WT_8h_PAA_H1_2.bedgraph
MOCK_H1_201106_2.bedgraph
HSV-1_Wt_str_F_H1_201106_2.bedgraph
ICP22_H1_201106_2.bedgraph
HSV-1_Wt_H1_new_Invitrogen_merged_200827.bedgraph
ICP22_H1_new_Invitrogen_merged_200827.bedgraph
MOCK_H1_new_Invitrogen_merged_200827.bedgraph
H3_total_WT17_180628.bedgraph
H3_total_mock_180628.bedgraph
H3_total_WT17_180424.bedgraph
H3_total_mock_180424.bedgraph
H3K36me3_wt_2.bedgraph
H3K27me3_wt_2.bedgraph
H3K36me3_mock_2.bedgraph
H3K27me3_mock_2.bedgraph

H3K27me3_wt_1.bedgraph
H3K36me3_wt_1.bedgraph
H3K27me3_mock_1.bedgraph
H3K36me3_mock_1.bedgraph
H3K36me3_wt_4.bedgraph
H3K36me3_mock_4.bedgraph

fastq files:
H3K27me3_mock_1:MOCK_H3K27me3_R1.fq.gz,MOCK_H3K27me3_R2.fq.gz
H3K27me3_mock_2:M24_H3K27me3_R1.fq.gz,M24_H3K27me3_R2.fq.gz
H3K27me3_wt_1:HSV_1_H3K27me3_R1.fq.gz,HSV_1_H3K27me3_R2.fq.gz
H3K27me3_wt_2:HSV29_H3K27me3_R1.fq.gz,HSV29_H3K27me3_R2.fq.gz
H3K36me3_mock_1:MOCK_H3K36me3_R1.fq.gz,MOCK_H3K36me3_R2.fq.gz
H3K36me3_mock_2:M23_H3K36me3_R1.fq.gz,M23_H3K36me3_R2.fq.gz
H3K36me3_mock_3:M43_H3K36me3_R1.fq.gz,M43_H3K36me3_R2.fq.gz
H3K36me3_wt_1:HSV_1_H3K36me3_R1.fq.gz,HSV_1_H3K36me3_R2.fq.gz
H3K36me3_wt_2:HSV28_H3K36me3_R1.fq.gz,HSV28_H3K36me3_R2.fq.gz
H3K36me3_wt_3:HSV48_H3K36me3_R1.fq.gz,HSV48_H3K36me3_R2.fq.gz
H3_total_WT17_1:HSV_1_H3_total_R1.fq.gz,HSV_1_H3_total_R2.fq.gz
H3_total_WT17_2:HSV27_Total_H3_R1.fq.gz,HSV27_Total_H3_R2.fq.gz
H3_total_mock_1:MOCK_H3_total_R1.fq.gz,MOCK_H3_total_R2.fq.gz
H3_total_mock_2:M22_Total_H3_R1.fq.gz,M22_Total_H3_R2.fq.gz
HSV-1_Wt_H1_1:200823_I66_V300060876_L4_HSV-1_Wt_H1_new_Invitrogen_1.fq.gz,200823_I66_V300060876_L4_HSV-1_Wt_H1_new_Invitrogen_2.fq.gz,200913_I66_V300063186_L2_HSV-1_Wt_H1_new_Invitrogen_1.fq.gz,200913_I66_V300063186_L2_HSV-1_Wt_H1_new_Invitrogen_2.fq.gz
HSV-1_Wt_str_F_H1_2:428_S1_L002_R1_001.fastq.gz,428_S1_L002_R2_001.fastq.gz
HSV1_17_WT_8h_PAA_H1_1:210730_M075_V350022578_L02_580_1.fq.gz,210730_M075_V350022578_L02_580_2.fq.gz,210730_M075_V350022578_L03_580_1.fq.gz,210730_M075_V350022578_L03_580_2.fq.gz
HSV1_17_WT_8h_PAA_H1_2:210730_M075_V350022578_L02_592_1.fq.gz,210730_M075_V350022578_L02_592_2.fq.gz,210730_M075_V350022578_L03_592_1.fq.gz,210730_M075_V350022578_L03_592_2.fq.gz
HSV1_17_WT_8h_PAA_H3_1:210730_M075_V350022578_L02_576_1.fq.gz,210730_M075_V350022578_L02_576_2.fq.gz,210730_M075_V350022578_L03_576_1.fq.gz,210730_M075_V350022578_L03_576_2.fq.gz
HSV1_17_WT_8h_PAA_H3_2:210730_M075_V350022578_L02_588_1.fq.gz,210730_M075_V350022578_L02_588_2.fq.gz,210730_M075_V350022578_L03_588_1.fq.gz,210730_M075_V350022578_L03_588_2.fq.gz
HSV1_17_WT_8h_PAA_H4_1:210730_M075_V350022578_L02_577_1.fq.gz,210730_M075_V350022578_L02_577_2.fq.gz,210730_M075_V350022578_L03_577_1.fq.gz,210730_M075_V350022578_L03_577_2.fq.gz
HSV1_17_WT_8h_PAA_H4_2:210730_M075_V350022578_L02_589_1.fq.gz,210730_M075_V350022578_L02_589_2.fq.gz,210730_M075_V350022578_L03_589_1.fq.gz,210730_M075_V350022578_L03_589_2.fq.gz
HSV1_17_WT_8h_PAA_IGG_1:210730_M075_V350022578_L02_594_1.fq.gz,210730_M075_V350022578_L02_594_2.fq.gz,210730_M075_V350022578_L03_594_1.fq.gz,210730_M075_V350022578_L03_594_2.fq.gz
HSV1_17_WT_8h_PAA_K27_1:210730_M075_V350022578_L02_579_1.fq.gz,210730_M075_V350022578_L02_579_2.fq.gz,210730_M075_V350022578_L03_579_1.fq.gz,210730_M075_V350022578_L03_579_2.fq.gz
HSV1_17_WT_8h_PAA_K27_2:210730_M075_V350022578_L02_591_1.fq.gz,210730_M075_V350022578_L02_591_2.fq.gz,210730_M075_V350022578_L03_591_1.fq.gz,210730_M075_V350022578_L03_591_2.fq.gz
HSV1_17_WT_8h_PAA_K36_1:210730_M075_V350022578_L02_578_1.fq.gz,210730_M075_V350022578_L02_578_2.fq.gz,210730_M075_V350022578_L03_578_1.fq.gz,210730_M075_V350022578_L03_578_2.fq.gz
HSV1_17_WT_8h_PAA_K36_2:210730_M075_V350022578_L02_590_1.fq.gz,210730_M075_V350022578_L02_590_2.fq.gz,210730_M075_V350022578_L03_590_1.fq.gz,210730_M075_V350022578_L03_590_2.fq.gz
ICP22_H1_1:200823_I66_V300060876_L4_ICP22_H1_new_Invitrogen_1.fq.gz,200823_I66_V300060876_L4_ICP22_H1_new_Invitrogen_2.fq.gz,200913_I66_V300063186_L2_ICP22_H1_new_Invitrogen_1.fq.gz,200913_I66_V300063186_L2_ICP22_H1_new_Invitrogen_2.fq.gz
ICP22_H1_2:432_S1_L002_R1_001.fastq.gz,432_S1_L002_R2_001.fastq.gz
MOCK_H1_1:200823_I66_V300060876_L4_MOCK_H1_new_Invitrogen_1.fq.gz,200823_I66_V300060876_L4_MOCK_H1_new_Invitrogen_2.fq.gz,200913_I66_V300063186_L2_MOCK_H1_new_Invitrogen_1.fq.gz,200913_I66_V300063186_L2_MOCK_H1_new_Invitrogen_2.fq.gz
MOCK_H1_2:424_S1_L002_R1_001.fastq.gz,424_S1_L002_R2_001.fastq.gz
Mock_8h_PAA_H1_1:210730_M075_V350022578_L02_573_1.fq.gz,210730_M075_V350022578_L02_573_2.fq.gz,210730_M075_V350022578_L03_573_1.fq.gz,210730_M075_V350022578_L03_573_2.fq.gz
Mock_8h_PAA_H1_2:210730_M075_V350022578_L02_586_1.fq.gz,210730_M075_V350022578_L02_586_2.fq.gz,210730_M075_V350022578_L03_586_1.fq.gz,210730_M075_V350022578_L03_586_2.fq.gz
Mock_8h_PAA_H3_1:210730_M075_V350022578_L02_569_1.fq.gz,210730_M075_V350022578_L02_569_2.fq.gz,210730_M075_V350022578_L03_569_1.fq.gz,210730_M075_V350022578_L03_569_2.fq.gz
Mock_8h_PAA_H3_2:210730_M075_V350022578_L02_582_1.fq.gz,210730_M075_V350022578_L02_582_2.fq.gz,210730_M075_V350022578_L03_582_1.fq.gz,210730_M075_V350022578_L03_582_2.fq.gz
Mock_8h_PAA_H4_1:210730_M075_V350022578_L02_570_1.fq.gz,210730_M075_V350022578_L02_570_2.fq.gz,210730_M075_V350022578_L03_570_1.fq.gz,210730_M075_V350022578_L03_570_2.fq.gz
Mock_8h_PAA_H4_2:210730_M075_V350022578_L02_583_1.fq.gz,210730_M075_V350022578_L02_583_2.fq.gz,210730_M075_V350022578_L03_583_1.fq.gz,210730_M075_V350022578_L03_583_2.fq.gz
Mock_8h_PAA_IGG_R_1:210730_M075_V350022578_L02_575_1.fq.gz,210730_M075_V350022578_L02_575_2.fq.gz,210730_M075_V350022578_L03_575_1.fq.gz,210730_M075_V350022578_L03_575_2.fq.gz
Mock_8h_PAA_K27_1:210730_M075_V350022578_L02_572_1.fq.gz,210730_M075_V350022578_L02_572_2.fq.gz,210730

_M075_V350022578_L03_572_1.fq.gz,210730_M075_V350022578_L03_572_2.fq.gz
Mock_8h_PAA_K27_2:210730_M075_V350022578_L02_585_1.fq.gz,210730_M075_V350022578_L02_585_2.fq.gz,210730_M075_V350022578_L03_585_1.fq.gz,210730_M075_V350022578_L03_585_2.fq.gz
Mock_8h_PAA_K36_1:210730_M075_V350022578_L02_571_1.fq.gz,210730_M075_V350022578_L02_571_2.fq.gz,210730_M075_V350022578_L03_571_1.fq.gz,210730_M075_V350022578_L03_571_2.fq.gz
Mock_8h_PAA_K36_2:210730_M075_V350022578_L02_584_1.fq.gz,210730_M075_V350022578_L02_584_2.fq.gz,210730_M075_V350022578_L03_584_1.fq.gz,210730_M075_V350022578_L03_584_2.fq.gz

bedgraph files:
Mock_8h_PAA_H3_1.bedgraph
Mock_8h_PAA_H4_1.bedgraph
Mock_8h_PAA_K36_1.bedgraph
Mock_8h_PAA_K27_1.bedgraph
Mock_8h_PAA_H1_1.bedgraph
HSV1_17_WT_8h_PAA_H3_1.bedgraph
HSV1_17_WT_8h_PAA_H4_1.bedgraph
HSV1_17_WT_8h_PAA_K36_1.bedgraph
HSV1_17_WT_8h_PAA_K27_1.bedgraph
HSV1_17_WT_8h_PAA_H1_1.bedgraph
Mock_8h_PAA_H3_2.bedgraph
Mock_8h_PAA_H4_2.bedgraph
Mock_8h_PAA_K36_2.bedgraph
Mock_8h_PAA_K27_2.bedgraph
Mock_8h_PAA_H1_2.bedgraph
HSV1_17_WT_8h_PAA_H3_2.bedgraph
HSV1_17_WT_8h_PAA_H4_2.bedgraph
HSV1_17_WT_8h_PAA_K36_2.bedgraph
HSV1_17_WT_8h_PAA_K27_2.bedgraph
HSV1_17_WT_8h_PAA_H1_2.bedgraph
MOCK_H1_201106_2.bedgraph
HSV-1_Wt_str_F_H1_201106_2.bedgraph
ICP22_H1_201106_2.bedgraph
HSV-1_Wt_H1_new_Invitrogen_merged_200827.bedgraph
ICP22_H1_new_Invitrogen_merged_200827.bedgraph
MOCK_H1_new_Invitrogen_merged_200827.bedgraph
H3_total_WT17_180628.bedgraph
H3_total_mock_180628.bedgraph
H3_total_WT17_180424.bedgraph
H3_total_mock_180424.bedgraph
H3K36me3_wt_2.bedgraph
H3K27me3_wt_2.bedgraph
H3K36me3_mock_2.bedgraph
H3K27me3_mock_2.bedgraph
H3K27me3_wt_1.bedgraph
H3K36me3_wt_1.bedgraph
H3K27me3_mock_1.bedgraph
H3K36me3_mock_1.bedgraph
H3K36me3_wt_4.bedgraph
H3K36me3_mock_4.bedgraph

Genome browser session
(e.g. UCSC)

https://genome.ucsc.edu/s/Caroline%20Friedel/dOCRs

Methodology

Replicates

n=2 for all conditions except for H3K36me3 in WT strain 17 without PAA (n=3)

Sequencing depth

Number of paired-end reads:
Mock_8h_PAA_H3_1:10245372
Mock_8h_PAA_H4_1:20775501
Mock_8h_PAA_K36_1:12059371
Mock_8h_PAA_K27_1:18019379
Mock_8h_PAA_H1_1:10581878
Mock_8h_PAA_IGG_R_1:40537501
HSV1_17_WT_8h_PAA_H3_1:18649680
HSV1_17_WT_8h_PAA_H4_1:22006736
HSV1_17_WT_8h_PAA_K36_1:11480085
HSV1_17_WT_8h_PAA_K27_1:28089060
HSV1_17_WT_8h_PAA_H1_1:12179488

Mock_8h_PAA_H3_2:16607763
Mock_8h_PAA_H4_2:21092241
Mock_8h_PAA_K36_2:12913679
Mock_8h_PAA_K27_2:18631952
Mock_8h_PAA_H1_2:13314357
HSV1_17_WT_8h_PAA_H3_2:15671094
HSV1_17_WT_8h_PAA_H4_2:21362414
HSV1_17_WT_8h_PAA_K36_2:13307380
HSV1_17_WT_8h_PAA_K27_2:19723269
HSV1_17_WT_8h_PAA_H1_2:10034588
HSV1_17_WT_8h_PAA_IGG_1:17841618
MOCK_H1_2:17199718
HSV-1_Wt_str_F_H1_2:12799762
ICP22_H1_2:14562527
HSV-1_Wt_H1_1:30132250
ICP22_H1_1:24566731
MOCK_H1_1:36952227
H3_total_WT17_2:22522286
H3_total_mock_2:17392552
H3_total_WT17_1:21250480
H3_total_mock_1:21039690
H3K36me3_wt_2:26643317
H3K27me3_wt_2:30005444
H3K36me3_mock_2:20669572
H3K27me3_mock_2:23530560
H3K27me3_wt_1:24115630
H3K36me3_wt_1:24767416
H3K27me3_mock_1:23698721
H3K36me3_mock_1:24602478
H3K36me3_wt_3:49913939
H3K36me3_mock_3:42786958

Number of mapped paired-end reads:
Mock_8h_PAA_H3_1:8118539
Mock_8h_PAA_H4_1:15742251
Mock_8h_PAA_K36_1:9758700
Mock_8h_PAA_K27_1:14792195
Mock_8h_PAA_H1_1:8456590
HSV1_17_WT_8h_PAA_H3_1:14216938
HSV1_17_WT_8h_PAA_H4_1:16851562
HSV1_17_WT_8h_PAA_K36_1:9236466
HSV1_17_WT_8h_PAA_K27_1:22117332
HSV1_17_WT_8h_PAA_H1_1:9654053
Mock_8h_PAA_H3_2:13142655
Mock_8h_PAA_H4_2:16515072
Mock_8h_PAA_K36_2:10585691
Mock_8h_PAA_K27_2:15433634
Mock_8h_PAA_H1_2:10593130
HSV1_17_WT_8h_PAA_H3_2:12362049
HSV1_17_WT_8h_PAA_H4_2:16728723
HSV1_17_WT_8h_PAA_K36_2:10656278
HSV1_17_WT_8h_PAA_K27_2:16380870
HSV1_17_WT_8h_PAA_H1_2:8025217
MOCK_H1_2:11283582
HSV-1_Wt_str_F_H1_2:9207526
ICP22_H1_2:10291893
HSV-1_Wt_H1_1:25506285
ICP22_H1_1:20552960
MOCK_H1_1:31251568
H3_total_WT17_2:18348168
H3_total_mock_2:14206360
H3_total_WT17_1:17251775
H3_total_mock_1:16935350
H3K36me3_wt_2:22324120
H3K27me3_wt_2:25318171
H3K36me3_mock_2:17322007
H3K27me3_mock_2:19803418
H3K27me3_wt_1:19867102

	H3K36me3_wt_1:20627165<br>H3K27me3_mock_1:19416607<br>H3K36me3_mock_1:20466756<br>H3K36me3_wt_3:41290248<br>H3K36me3_mock_3:35605265
Antibodies	1:100/IP of anti-H1 antibody (Invitrogen #PA5-30055), 1:50/IP of anti-H3 antibody (Invitrogen, PA5-16183), 1:50/IP of anti-H4 antibody (Cell Signaling, #14149S), 1 µg/IP of anti-H3K27me3 (Diagenode, #C15410195) and 1 µg/IP of anti-H3K36me3 (Diagenode, #C15410192)
Peak calling parameters	F-Seq with default parameters
Data quality	Data quality was evaluated using FastQC (v0.11.9), ATACseqQC (v1.14.4) and ChIPseeker (v1.26.2)
Software	fastQC (v0.11.9), ContextMap (v2.7.9), BWA (v0.7.10), samtools (v1.3), R (v4.0.3), F-seq, BEDTools (v2.29.1), ATACseqQC (v1.14.4), ChIPseeker (v1.26.2)
